# Modular Design of Artificial Tissue Homeostasis: Robust Control through Synthetic Cellular Heterogeneity

**DOI:** 10.1371/journal.pcbi.1002579

**Published:** 2012-07-19

**Authors:** Miles Miller, Marc Hafner, Eduardo Sontag, Noah Davidsohn, Sairam Subramanian, Priscilla E. M. Purnick, Douglas Lauffenburger, Ron Weiss

**Affiliations:** 1 Department of Biological Engineering, Massachusetts Institute of Technology, Cambridge, Massachusetts, United States of America; 2 School of Computer and Communication Sciences, Ecole Polytechnique Fédérale de Lausanne (EPFL), Lausanne, Switzerland; 3 Department of Biochemistry, University of Zurich, Zurich, Switzerland; 4 Swiss Institute of Bioinformatics, Lausanne, Switzerland; 5 Department of Mathematics, Rutgers University, New Brunswick, New Jersey, United States of America; 6 Department of Electrical Engineering, Princeton University, Princeton, New Jersey, United States of America; 7 Department of Gene and Cell Medicine, Black Family Stem Cell Institute, Mount Sinai School of Medicine, New York, New York, United States of America; National University of Singapore, Singapore

## Abstract

Synthetic biology efforts have largely focused on small engineered gene networks, yet understanding how to integrate multiple synthetic modules and interface them with endogenous pathways remains a challenge. Here we present the design, system integration, and analysis of several large scale synthetic gene circuits for artificial tissue homeostasis. Diabetes therapy represents a possible application for engineered homeostasis, where genetically programmed stem cells maintain a steady population of *β*-cells despite continuous turnover. We develop a new iterative process that incorporates modular design principles with hierarchical performance optimization targeted for environments with uncertainty and incomplete information. We employ theoretical analysis and computational simulations of multicellular reaction/diffusion models to design and understand system behavior, and find that certain features often associated with robustness (e.g., multicellular synchronization and noise attenuation) are actually detrimental for tissue homeostasis. We overcome these problems by engineering a new class of genetic modules for ‘synthetic cellular heterogeneity’ that function to generate beneficial population diversity. We design two such modules (an asynchronous genetic oscillator and a signaling throttle mechanism), demonstrate their capacity for enhancing robust control, and provide guidance for experimental implementation with various computational techniques. We found that designing modules for synthetic heterogeneity can be complex, and in general requires a framework for non-linear and multifactorial analysis. Consequently, we adapt a ‘phenotypic sensitivity analysis’ method to determine how functional module behaviors combine to achieve optimal system performance. We ultimately combine this analysis with Bayesian network inference to extract critical, causal relationships between a module's biochemical rate-constants, its high level functional behavior in isolation, and its impact on overall system performance once integrated.

## Author Summary

Over the last decade several relatively small synthetic gene networks have been successfully implemented and characterized, including oscillators, toggle switches, and intercellular communication systems. However, the ability to engineer large-scale synthetic gene networks for controlling multicellular systems with predictable and robust behavior remains a challenge. Here we present a novel combination of computational methods to aid the iterative design and optimization of such synthetic biological systems. We apply these methods to the design and analysis of an artificial tissue homeostasis system that exhibits coordinated control of cellular proliferation, differentiation, and cell-death. Achieving artificial tissue homeostasis would be therapeutically relevant for diseases such as Type I diabetes, for instance by transplanting genetically engineered stem cells that stably maintain populations of insulin-producing beta-cells despite normal cell death and autoimmune attacks. To manage complexity in the design process, we employ principles of logic abstraction and modularity and investigate their limits in biological networks. In this work, we find factors often associated with robustness (e.g., multicellular synchronization and noise attenuation) to be actually detrimental, and overcome these problems by engineering genetic modules that generate beneficial population heterogeneity. A combination of computational methods elucidates how these modules function to enhance robust control, and provides guidance for experimental implementation.

## Introduction

One of the key challenges facing synthetic biology today is the ability to engineer large-scale, multicellular systems with sophisticated yet predictable and robust behaviors. Previous work in synthetic biology has successfully implemented and characterized a variety of relatively small synthetic gene networks including oscillators [1]–[4], toggle switches [5], [6], and intercellular sender/receiver or quorum sensing (QS) communication systems [7]–[9]. Computational tools have encouragingly demonstrated an ability to guide experimental optimization of several of such modules [10], [11], and some recent projects have successfully integrated a few of these ‘standard modules’ and interfaced them with endogenous pathways to program more sophisticated behaviors [12]–[18]. Ultimately, however, the path to success will require bridging the gap between specifying sophisticated systems-level objectives and a list of molecular parts and interactions that can be properly assembled to accomplish these objectives [19]. To address this challenge, here we present and apply a novel combination of computational methods to aid the iterative design and optimization of synthetic biological systems. Importantly, these tools address issues stemming from the incomplete and imprecise knowledge of rate constants and cellular context.

As a case study, we design a system to control tissue homeostasis, broadly defined as the property of balancing growth, death, and differentiation of multiple cell-types within a multicellular community. Tissue homeostasis represents an important class of problems in biology, and the ability to control it is fundamental to the success of a wide range of tissue engineering goals. At the same time the ability to create and analyze such a system may provide insight into mechanisms of endogenous tissue homeostasis and its misregulation in diseases such as cancer and diabetes. For example, misregulation of tissue homeostasis plays a central role in Type I diabetes, in which natural populations of insulin-producing 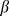-cells are destroyed due to autoimmune defects. Automated mechanical systems have been proposed for insulin control in diabetes but still face significant challenges including long-term efficacy [20]. Stem cell and 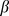-cell transplantations have also been studied as possible solutions [21], [22], but the last decade of results suggest that the transplanted cells fail to maintain homeostasis and become either tumorigenic or depleted within months [23].

### Approach

**Figure 1:**
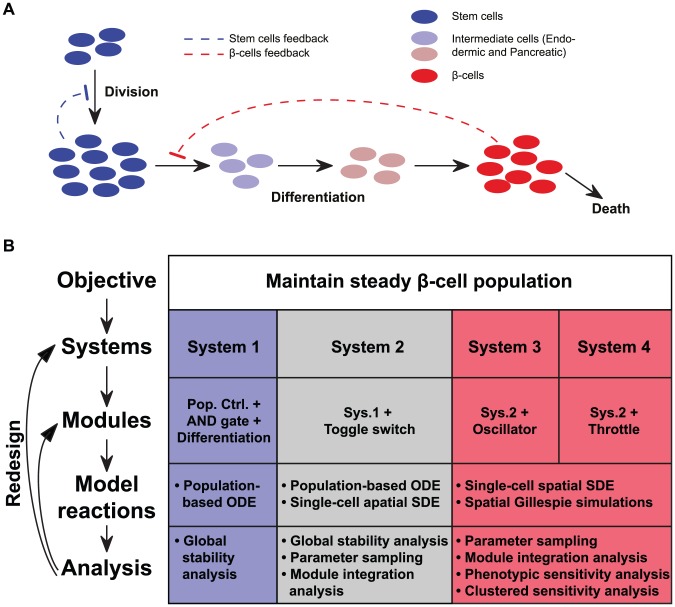
Overview of system design. (A) The general tissue homeostasis design. Proliferation of stem cells (blue) is regulated by their population size through negative feedback (dashed blue line). Sequential differentiation into endodermic, pancreatic, and finally 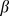-cells (red) occurs when the stem cell population has sufficient size, and is governed through negative feedback from differentiated cells (dashed red line). (B) Design workflow. Starting with a high-level objective, iterative design proceeds through a top-down decomposition into modules and then basic reactions of the system, followed by analysis and redesign (left). The table columns (right) show the four iterations of system designs presented in this work. Table rows describe the top-down decomposition for each system, and correspond to the workflow at left.

As potential solutions for this problem, we propose several increasingly robust variants of a synthetic gene network that are designed to maintain a steady level of 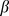-cells despite normal cell death and constant destruction of the 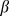-cells by the immune system. The synthetic gene networks continuously direct proliferation, quiescence, and stem cell differentiation into insulin producing 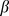-cells as needed (Figure 1A). The resulting engineered circuits may be employed to regulate tissue homeostasis both *in vitro* where the cell culture is removed from natural cues, and *in vivo* when natural systems fail or tissue is ectopically transplanted (for example, the Edmonton protocol involves implanting pancreatic islets including 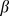-cells to the liver [24]).

The efforts described here are based on encouraging genetic engineering accomplishments that have demonstrated population control of bacteria and yeast [12], [25], mammalian cell proliferation [26], and stem cell differentiation [27], [28]. To mitigate some of the uncertainties involved in system construction, we restricted our designs to use only genetic parts and modules that have already been demonstrated experimentally. These include engineered cell-cell communication to determine population densities, a toggle switch, an oscillator, and a multi-input AND gate.

To gain a detailed understanding of our proposed synthetic gene networks, we carried out theoretical analysis and computational simulations using Ordinary Differential Equations (ODE's), Langevin, and Gillespie algorithms. The analysis revealed that while simple modular composition was useful for initial system design, various factors such as stochastic effects, feedback control, and module interdependence significantly impacted system function and hence had to be taken into account when evaluating system designs. Strikingly, we observed that system features typically associated with robustness, including cell-synchronization, noise attenuation, and rapid signal processing destabilized our systems. To overcome these problems, we propose and analyze mechanisms that generate population diversity, and through this symmetry breaking facilitate proportionate and homeostatic system response to population-wide cues. Endogenous mechanisms of cellular heterogeneity have been previously observed in many physiological processes, including differentiation [29]. In the synthetic biology context, however, these mechanisms may be either unavailable for integration into the synthetic genetic circuit or too poorly understood to fully utilize. As a result, we forward engineer modules to generate synthetic cellular heterogeneity. For example, we incorporate an asynchronous oscillator module into the design as an engineered generator of intrinsic variability. Ultimately, our analysis indicates that such modules greatly improve homeostatic robustness among an isogenic population of cells, and we identify several examples of natural analogs.

### Key results

We found that the design and optimization of modules for synthetic heterogeneity is both non-intuitive and multifactorial, and in general requires a framework for non-linear and multivariate analysis. For example, with the asynchronous oscillator, we could not *a priori* define a simple objective or ideal ‘phenotype’ since oscillator properties such as period, dynamic range, and asynchronicity affected overall system performance in complex and interdependent manners. Furthermore, even if ideal module phenotypes are known, understanding the physical parameters required to achieve such phenotypes also represents a challenge. To address these issues, we developed a new framework using a hierarchy of computational tools to understand the optimal phenotypic and physical characteristics of the synthetic heterogeneity modules with respect to overall system behavior. We developed a ‘phenotypic sensitivity analysis’ method to determine how functional module behaviors combine to achieve optimal system performance. Parametric sensitivity analysis then captures the dependency of a module's phenotypes on its underlying physical rate constants. Ultimately, we integrated both analyses using Bayesian network inference to extract critical, causal relationships between a module's biochemical rate constants, its high level functional behavior in isolation, and its impact on overall system performance once integrated. Importantly, we anticipate that our hierarchical optimization strategy prescribes directions for system design that readily apply to experimental systems facing high degrees of uncertainty in rate constants and cellular environment.

### Outline

We designed and modeled an artificial tissue homeostasis system where a population of self-renewing stem cells grow and differentiate in a regulated manner to sustain a steady population of adult cells which, in this case, are insulin-producing 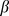-cells (Figure 1A). Here we present four iterations of system design, analysis, and redesign with increased sophistication for improved robustness in controlling tissue homeostasis (Figure 1B). The initial model for artificial tissue homeostasis (System 1) comprises four integrated modules, and is analyzed using ODE simulation and global stability analysis. We incorporate a toggle switch in System 2 to minimize undesired 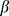-cell population fluctuations observed in System 1, and analyze the improved design using stochastic differential equations (SDEs). Although System 2 represents an improvement, its homogeneous response to commitment cues results in poor performance, thereby motivating the incorporation of an oscillator module and a throttle module for Systems 3 and 4, respectively. Using SDE simulations, we optimize these modules and their integration into the full system. Throughout the discussion, we focus on several aspects of system design, including module integration, optimization of rate constants for individual modules, and optimization of module phenotypic behaviors.

## Results

### Iterative system design and analysis

**Figure 2:**
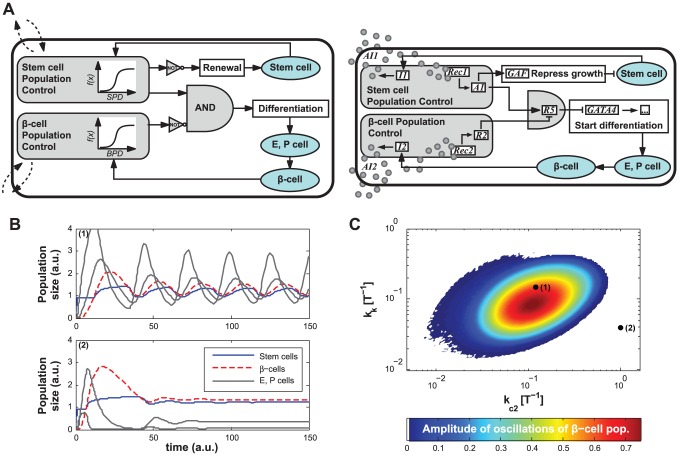
System 1. (A) Circuit diagram: two Population Control modules (in gray) sense the density of stem- and 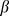-cells. The AND gate integrates the output of the modules to induce differentiation. Circles represent intercellular signaling molecules. (B) Two examples of population evolution showing sustained oscillations (point 1 in *C*) and a stable steady state (point 2 in *C*), with other parameters fixed (SI Sec. 2 and Figure S2). (C) A planar slice of the parameter space where population oscillations occur for System 1.

Simple mathematical analysis suggested that feedback regulation between the two populations of stem cells and adult cells was necessary for robust homeostatic control, and recent work has explored the essential role of feedback control in stem cell biology (Text S1, Sec. 2.1, [30]). In all alternative system designs presented in this manuscript, we implemented feedback control through artificial cell-cell communication pathways. Our first design, System 1, allows differentiation only with a high density of stem cells and a low density of 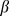-cells (Figure 2A). The “Stem Cell Population Control” (SPC) module allows for differentiation only when the population density of self-renewing cells lies above some threshold. We also designed the SPC to suppress proliferation through the expression of a growth arrest factor (GAF), currently under development in the Weiss lab. The “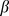-Cell Population Control” (BPC) module produces high output and inhibits differentiation when the density of 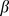-cells reaches a threshold (Figure 2A). We based the cell-cell communication systems in the SPC and BPC modules on previously described communication systems [7]–[9]. As a proof of concept, Supplementary Figure S1 A–B presents results for a signal-receiver circuit based on the LuxR protein that responds to 3-oxo-hexanoyl-homoserine lactone (3OC6HSL), that has been experimentally implemented in human embryonic kidney (HEK293) cells.

We model stem cell differentiation as a multistage process that can take several weeks to complete [31]. For example, directed *in vitro* differentiation of hES cells into insulin-producing cells involves stepwise administration of growth factors to first induce endodermal cell fate, followed by pancreatic specialization, expansion, and maturation [32]. This general process is modeled by four cell types: stem cells (population size 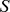) grow with a constant division rate 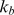. Upon maturation, they proceed through two intermediate populations of endodermic (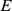) and pancreatic (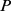) cells before becoming 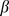-cells (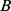), which die at a constant rate 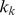. We describe the sequential maturation of 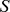 into 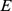, 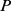, and 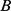 as first-order reactions with rates 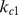, 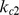, and 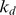. Feedback terms are modeled as Hill functions, where 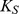 and 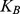 represent the SPC and BPC module thresholds, respectively.
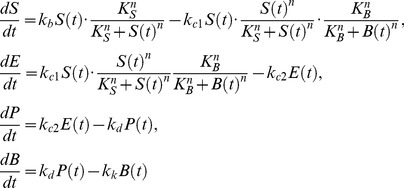
(1)


The differentiation process is generally long *in vivo* (e.g., 20 days [32]. For System 1, such delay in the feedback could induce undesirable oscillations (Figure 2B–C). As a result, System 1 failed to maintain homeostasis for a large range of parameter values (Text S1, Sec. 2).

#### Toggle switch facilitates rapid feedback but neglects the heterogeneity requirement

**Figure 3:**
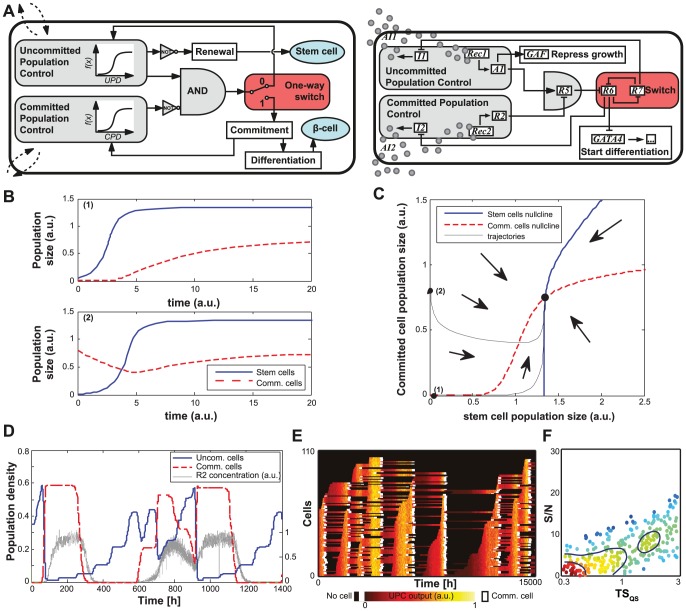
System 2. (A) Circuit diagram: two Population Control modules sense the density of stem and committed cells. The AND gate integrates the output of the modules to induce commitment through the switch state (red module). (B) Deterministic time trajectories for System 2 with two different initial conditions: both converge to the same equilibrium populations. (C) Phase space diagram: all trajectories converge to a unique equilibrium point. Black lines correspond to trajectories plotted in *B*. See Text S1, Sec. 2 and Figure S3 for other phase space diagrams. (D) Stochastic trajectories for a simulation starting with a small stem cell population, showing the output of the Committed Population Control module (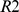) in representative uncommitted cells (right axis, a.u.). (E) Individual rows track the single-cell UPC module output (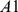, shown as a heat map) in uncommitted cells within a population. White signifies single-cell commitment, followed by black “null space” that is filled by newly divided uncommitted cells. As soon as UPC output is high (yellow), stem cells commit *en masse*. (F) Overall system performance, S/N, as a function of the module time-scale for cell communication, 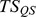. Several hundred different sets of time-scales were tested, with all time-scale parameters simultaneously varied. Each point represents an individual set of time-scales. Color and contour lines indicate point density.

System 2 minimizes feedback delay by using a ‘commitment’ module to decouple the BPC module from the slow differentiation process (Figure 3A). Commitment occurs through a one-way toggle switch, which we designed to reflect earlier computational models [33] and an *E. coli* implementation [34]. As a first step and proof of concept, Supplementary Figure S1 C–D presents an experimental implementation of the proposed toggle switch in human cells. In System 2, the toggle activates both differentiation and population feedback, such that the feedback control is immediately downstream of the toggle switch rather than following the full differentiation process (Figure 3A). The state of the one-way switch defines whether or not the cell has irreversibly committed to differentiate, and this status feeds back into what we now term the “Uncommitted Population Control” (UPC) and “Committed Population Control” (CPC) modules. The density of cells in any stage of the differentiation process determines CPC module output. Consequently, we gained a faster feedback response in exchange for assuming that a relatively constant fraction of cells successfully differentiate upon commitment. Accordingly, in our model for System 2, the rate of the first stage of differentiation (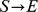 in Eq. (1)) is now (other equations remain the same):
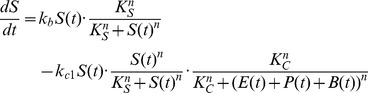
(2)


Compared to System 1, the population sizes quickly equilibrated in System 2 (Supplementary Figure S2). We further tested different initial conditions and parameter vectors, and found the System 2 equilibrium point to be independent of the initial conditions (Text S1, Sec. 2.2).

For subsequent analyses, we simplified our model to a two-population system. Given that 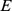, 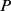 and 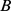 populations are identical with respect to feedback, we merged them into the committed population 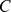, resulting in the following equations for System 2:
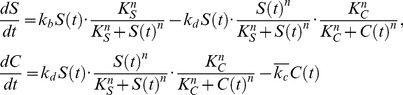
(3)In this ODE model, the actual 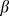-cell population 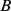 is a fraction of this committed population 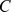 (Text S1, Sec. 2.3). We note that a two population system may not fully restore the complexity of a four population system, for example by precluding chaotic behavior. Nonetheless, the two population model showed a qualitatively similar behavior in the working range of our system. Henceforth, we focused on maintaining a constant population of committed rather than differentiated cells. Indeed, this system demonstrated a stable equilibrium point in a large range of conditions (Figure 3B–C, S3 and proof in Text S1, Sec. 2.4).

Our deterministic model of continuous population dynamics suggested that System 2 stabilized homeostasis sufficiently. However, low molecular count, small population size, and localized reaction/diffusion may constitute critical determinants of system dynamics [35], [36]. To obtain an improved understanding of how these factors affect system behavior, we performed spatiotemporally-resolved simulations of multicellular populations using stochastic differential equations (Text S1, Sec. 3), assuming Hill functions for inhibition and activation relations.

These simulations revealed that phenotypic homogeneity within the isogenic stem cell population impedes system performance. More specifically, strong population-wide cues to commit may cause massive simultaneous commitment, thereby depleting the stem-cell pool and leading to homeostasis failure (Figure 3D–E). To quantify system performance, we employed a signal to noise ratio (S/N) metric (inverse of the coefficient of variation, see Text S1, Sec. 3) that denotes how steady the committed population density is maintained. As an initial analysis of overall system robustness, we explored how S/N was affected by variations in the time-scales with which individual modules operate. We lumped system parameters according to their module (Text S1, Sec. 5.2.1, and Table S2) and adjusted them in a coordinated manner to change only how fast a module processed incoming signals and produced the appropriate output, while keeping steady-state behavior of individual modules constant. Perturbing time-scales for modules such as the toggle switch and cell-cell communication randomly and simultaneously allowed us to observe how robust S/N was across the range of time-scales. For System 2, S/N was very sensitive to module time-scales, and most combinations of time-scales resulted in a poorly functional system (Figure 3F). Relative to other processes in the system, rapid feedback kinetics described by the ‘quorum sensing’ cell-cell communication time-scale (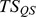) could decrease the simultaneous commitment observed in Figure 3D–E, but it may not be possible to implement such a fast response in practice. Moreover, significant environmental perturbations to the system, for example resulting from injury or elevated autoimmune response, could still provoke situations where System 2 fails to maintain homeostasis. We therefore implemented synthetic modules that generate phenotypic heterogeneity in an isogenic population. These modules desynchronize single-cell responses to population-wide signaling cues, thereby facilitating a proportionate and homeostatic system response and balancing the necessity for a fast quorum sensing.

#### Oscillator stabilizes through asynchronicity

**Figure 4:**
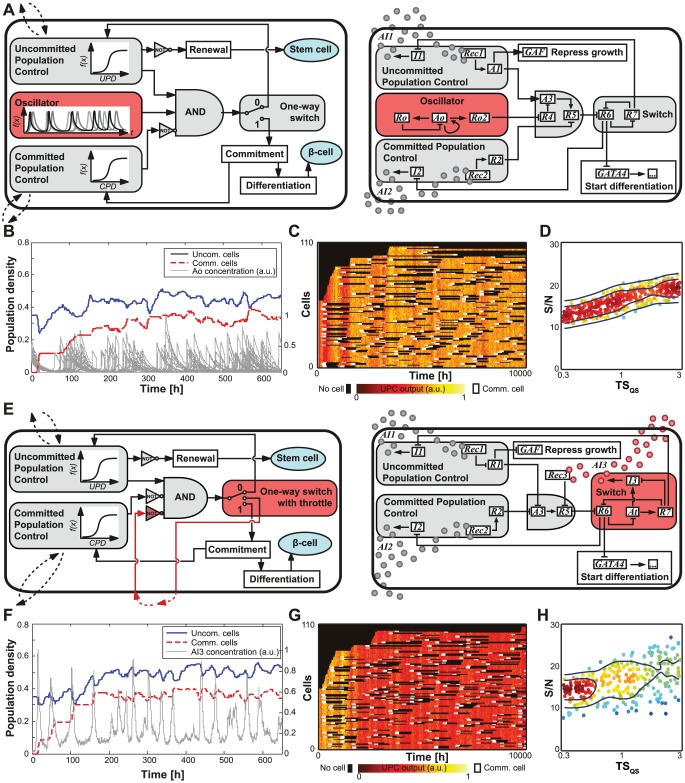
Systems 3 and 4. (A) Circuit diagram for System 3: in addition to System 2 modules, the AND gate integrates the output of the oscillator (red module) that allows commitment only when peaking. (B) Time trajectories for a simulation starting with a small stem cell population. The oscillator activator (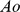) is plotted for some representative stem cells (right axis, a.u.). (C) Individual rows track the single-cell UPC module output (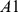, shown as a heat map) in uncommitted cells within a population. White signifies single-cell commitment, followed by black “null space” that is filled by newly divided uncommitted cells. Due to the oscillator, only a fraction of the cells commit when the 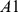 concentration is high. (D) Overall system performance, S/N, as a function of the module time-scale for cell communication, 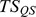. Several hundred different sets of time-scales were tested, with all time-scale parameters simultaneously varied. Each point represents an individual set of time-scales. Color and contour lines indicate point density. (E) Circuit diagram for System 4: a throttle mechanism (red module) activates during a cell's commitment and represses commitment in its neighbors. (F–G) Time trajectories for a simulation starting with a small stem cell population, where *B* shows the average throttle signaling component (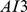) in the external medium (right axis, a.u.) over time. (H) S/N as a function of the module time-scale for cell communication, 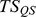.

In System 3, we incorporated an asynchronous oscillator (e.g. [3], [4]) into the design as a generator of intrinsic heterogeneity (Figure 4 A). In this system, a cell's commitment to differentiation can only occur when its oscillator peaks (and 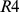 concentration is low). Stochasticity drives individual oscillators out of phase, and coupling the oscillator to cell-fate decisions prevents cells in a population from all simultaneously responding to homogeneous commitment signals. Simulations indicated that with the oscillator, our system maintained tissue homeostasis robustly despite the fact that feedback signaling cues to commit remained synchronized even after homeostasis was established (Figure 4B–C). Compared to System 2, System 3 behaved much more robustly to variations in module time-scales, with more than double the S/N of System 2 when averaged across all tested time-scales (Figure 4D). Although our analysis suggested that the oscillator would be a powerful addition to the system design, unforeseen experimental factors may hamper its successful implementation. Unaccounted for drivers of oscillator synchronization across a population, for example, could negatively impact system performance. To address this issue, we developed an alternate strategy for generating population diversity (System 4). Subsequent analysis of these systems then allows us to compare their specific advantages and disadvantages.

#### Commitment throttle stabilizes through local inhibition

System 4 achieves population heterogeneity through rapid lateral inhibition acting as a throttle on the commitment process during toggle switching (Figure 4E). Through this mechanism, a cell starting to commit blocks the commitment process of adjacent cells. The throttle approach requires a third intercellular signaling molecule that is synthesized transiently while the toggle switches and temporary inhibits neighboring cells from committing likewise. The rest of the circuit remains similar to previous systems. Simulations indicated that when populations reached their steady state values, the throttle mechanism prevented simultaneous commitment of too many cells and therefore maintained homeostasis (Figure 4F–G). Consequently, System 4, like System 3, behaved more robustly to variations in module time-scales compared to System 2 (Figure 4H). Although Systems 3 and 4 clearly outperformed System 2 in these simulations, appreciable differences in time-scale robustness among the three systems warrant further analysis, and the following section explores this from a multivariate perspective.

### Robustness analysis and optimization

The integration of several network modules presents a challenge on multiple levels, especially in the context of uncertain biological environments and complex module dynamics. In the following sections, we introduce a framework composed of computational modeling and analysis techniques that addresses these issues in optimizing Systems 2, 3 and 4. We first study overall system robustness to external parameters such as cell survival dynamics, and introduce time-scale analysis as a method for guiding module integration. We then optimize the population control module using a novel ‘clustered sensitivity analysis’ to comprehend global patterns of parametric sensitivity in the context of a detailed biochemical model. Finally, we analyze the synthetic heterogeneity modules with an approach that focuses on module phenotype rather than rate constants alone. Comparisons among the different system architectures ultimately provide guidance for experimental optimization.

#### Synthetic heterogeneity enhances robustness to noise and cell survival times

**Figure 5:**
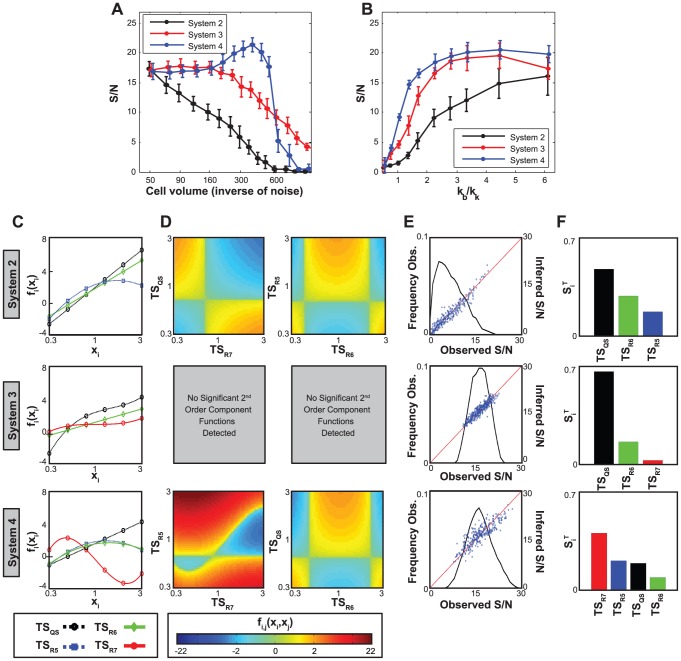
Robustness analyses and time-scale optimization for Systems 2–4. (A) S/N for different cell volume 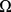, which corresponds to the number of molecules in each cell. (B) S/N for different ratios of stem cell division rate (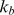) and 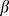-cell killing rate (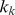). (C–D) RS-HDMR analysis of Systems 2–4 to changes in the reaction time-scales of module components. (C) First- and (D) second-order RS-HDMR component functions describe the relationship between reaction time-scales (normalized to [0,1]) and the corresponding S/N observed in the overall system. (E) Distribution of S/N observed in response to time-scale parameter sampling (black) and RS-HDMR inference accuracy of that variation (blue). (F) Total sensitivity indices (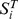) of the module time-scales observed for each system.

We first explored the impact of stochasticity on homeostasis by adjusting the simulated cell volume, 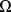, which is related to the number of molecules in each cell (Text S1, Sec. 3). Increasing noise, by decreasing 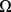, impacted homeostasis performance both positively and negatively, depending on several factors. Without either the oscillator or throttle, System 2's S/N value decreased monotonically with decreased noise (Figure 5A). In contrast, S/N values for Systems 3 and 4 displayed biphasic dependency on 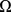. For small 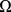, Systems 3 and 4 showed the same performance as System 2, largely because high noise obscured feedback signals. Intermediate values of 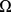 allowed the oscillator and throttle to generate optimally heterogeneous population responses. The S/N observed for large 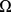 was low for all systems due to more synchronous cellular commitment during the dynamic establishment of homeostasis, emphasizing the importance of stochasticity for generating heterogeneity in homeostasis regulation.

We analyzed the robustness of the systems to another external parameter, the average committed-cell survival time (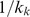), which may fluctuate *in vivo*, by simulating system behavior with different ratios of uncommitted-cell division rate to committed-cell killing rate (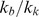). In general, Systems 3 and 4 exhibited greater robustness to decreasing (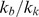) compared to System 2 (Figure 5B and Text S1, Sec. 2.5). We also analyzed the effect of the parameter 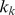 on the homeostatic population size. Equilibrium populations remained near the desired homeostatic levels for high (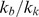), but could decrease at lower ratios (Figure 5B). Ultimately, the robustness to noise and cell survival times underscores the need for heterogeneity within the population, and provides further evidence that the synthetic heterogeneity generated from the oscillator and throttle improves system performance over a range of parameters.

#### Intermodular time-scale matching reveals system dependent module coupling

In our system, accurate cell decision processing requires the appropriate integration of modules that generally have well defined behaviors in isolation. Even if we assume input-output behavior that meets our design specifications for each module (see Text S1, Sec. 3), integrating these modules together still presents a challenge. As introduced in Figures 3F and 4D,H, we explored system robustness to variations in the time-scales with which individual modules operate.

We used the Random-Sampling High Dimensional Model Representation (RS-HDMR) algorithm [11], [37] (Text S1, Sec. 5.1) to understand both the individual and cooperative nonlinear effects of time-scale modulation on S/N (Figure 5C–F and S5). RS-HDMR describes the independent and cooperative effects of inputs, which in this analysis are module time-scales, on an output, the S/N value, in terms of a hierarchy of interpretable RS-HDMR component functions. Importantly, RS-HDMR supports global parametric sensitivity analysis, which is appropriate in this work where precise parameter values (time-scales in this case) may be highly uncertain. The first-order component function 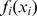 describes the generally non-linear independent contribution of the 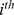 input variable to the output. For System 2, first-order RS-HDMR component functions showed that fast diffusion and a rapid toggle switch (through 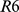 dynamics) contribute to good system performance. Second-order RS-HDMR component functions indicated cooperative interactions among parameters. Here, parameters correspond to individual modules; therefore, we interpreted cooperative relationships as ‘intermodular coupling’ (Figure 5D). For example, having a fast toggle switch (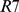) dynamics in System 2 offset the detrimental impact of slow diffusion. For System 3, the only significant correlations between performance and time scales were found for diffusion and, to a lesser extent, the toggle switch (Figure 5C). Interestingly, RS-HDMR detected no significant second-order component functions in System 3. These results indicated that the oscillator, in effect, decoupled the modules from each other, minimizing cooperative interactions between diffusion and the toggle switch by creating a buffer between the two. Compared to Systems 2 and 3, System 4 performance exhibited a more complex dependency on time-scale parameters, indicated by its significant second-order functions (Figure 5C–D). In particular, the cooperative interaction of slow 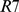 dynamics combined with fast 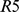 dynamics produced a strong synergistic improvement in S/N. This combined effect facilitates effective 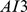-mediated lateral inhibition while the toggle switches. Total sensitivity indices represent the summed weight of first- and second-order RS-HDMR component functions for each parameter (Figure 5F). For Systems 2 and 3, observed S/N was most sensitive to changes in diffusion (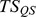). In contrast, toggle-switch dynamics (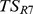) most significantly affected performance in System 4. Of note, optimal time-scale matching yielded an improvement for all systems in robustness to molecular noise and cell survival dynamics, particularly under conditions of relatively fast cell death (Supplementary Figure S6). Overall, analysis of intermodular time-scale matching prescribes strategies for integrating modules and suggests ways in which module dynamics can be coordinately manipulated to yield improved system performance *in vitro*.

#### Clustered sensitivity analysis for targeted optimization

**Figure 6:**
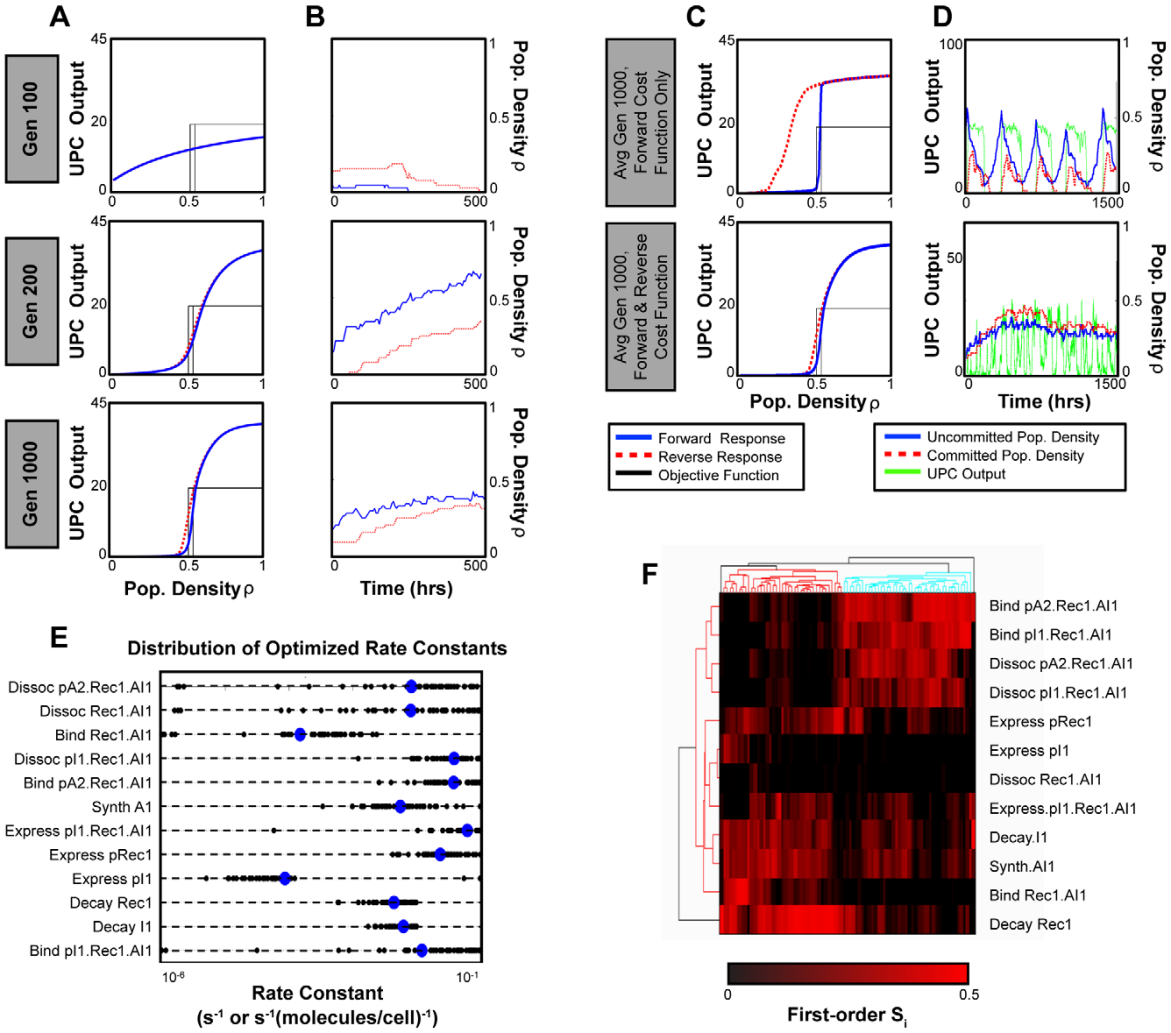
Parametric optimization of the UPC module. (A) GA optimization progress for three representative generations, using an ODE model of the UPC module. The GA objective function is a three-component step-function, with zero UPC activity below a defined threshold, an ignored transition region, and high activity above the transition region. (B) Gillespie simulations of System 3, corresponding to optimization progress in *A*. (C) Average UPC module transfer curves when the reverse response is either excluded or included in the subnetwork GA optimization. (D) Full system behavior corresponding by row to the module optimization results in *C*. (E) Distribution of rate constants for the optimized parameter vectors determined by 75 independent GA runs of 1000 generations each, using both forward and reverse response objective functions. (F) Clustered sensitivity analysis of the UPC Module. Each column corresponds to a “parameter sensitivity signature” for each of the 75 local parameter neighborhoods that we sampled; rows correspond to the analyzed parameters of the UPC module. First-order sensitivity values shown in the heat map range from 0.0 (black) to 0.5 (red).

We also modeled System 3 using the Gillespie algorithm to explicitly account for binding and transcription events (for example, the binding of the receiver protein Rec1 to its inducer AI1, *Bind Rec1.AI1*, Text S1, Sec. 4). Results presented in the previous section were based on Langevin models that assume Hill functions for all inhibition and activation interactions, but our initial results with the Gillespie model suggested that achieving useful sigmoidal responses in the UPC module may be particularly challenging. Note that Systems 2 and 4 share the same UPC module as System 3 and the following results are valid for all systems. Figure 6A–B demonstrates how excess UPC output below the threshold (first row) or insufficient output above the threshold (second row) in suboptimal systems can lead to overactive commitment or proliferation, respectively. Consequently, we focused on optimizing the UPC module to obtain a step-like response to population density, 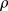. We incorporated positive feedback in the UPC module, and then employed a genetic algorithm (GA) to optimize module parameters. The GA allowed us to efficiently navigate the high-dimensional parameter space and avoid local minima in the optimization process [38]. However, initial optimization of the module's rate constants only considered scenarios where the population densities increased (“forward response”). Unfortunately, this generated hysteresis, where high UPC output is maintained as 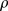 decreases below the threshold level (similar to [39]). Such hysteresis can lead to sub-optimal or even non-functional tissue homeostasis performance (Figure 6C–D, first row). Consequently, we also took into account the “reverse response” in the optimization process, which describes UPC output under conditions of decreasing cell density. Our GA optimization then successfully generated a diverse ensemble of rate constants, each yielding UPC networks with positive feedback that exhibited both step-like and non-hysteretic behavior (Figure 6C, second row). These optimized subnetworks produced stable homeostasis when integrated in the full-system Gillespie model (Figure 6D, second row).

We performed RS-HDMR analysis of the UPC subnetwork to understand how rate constants affect hysteresis, which would help guide the experimental construction of the system. We examined local parameter “neighborhoods” around each GA-generated vector of optimized parameters from Figure 6E (Text S1, Sec. 5.3). Our sensitivity analysis suggested that systems displaying similar UPC behavior can have drastically different responses to similar changes in rate constants: each parameter neighborhood that we analyzed had a distinct signature of parametric sensitivity (Figure 6F). We clustered parametric neighborhoods based on these signatures. Despite differences in individual sensitivities, the clustered sensitivity analysis revealed that the majority of signatures fell into two main clusters, each with distinctive features. For example, in one cluster (red on the dendrogram) the decay rate of the receptor protein 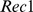 (rate *Decay Rec1*) significantly affected hysteresis, while the binding and dissociation rates of 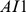-bound 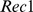 complex (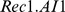) had little influence. The opposite was true for the other cluster (cyan on the dendrogram).

When building genetic networks experimentally, precise parameter values and their influence on system behavior may be unknown, presenting a challenge for optimization. Logistical constraints limit the number of parameters that can be reasonably manipulated, but clustered sensitivity analysis can act as a guide for iteratively prioritizing which parameters to mutate. In our system, for example, results suggest that we manipulate the most sensitive parameters from each of the two main clusters (*Decay Rec1* and the binding of the Rec1-AI1 complex to its promoter, *Bind pA2.Rec1.AI1*). At least one of these two parameter manipulations is likely to reduce hysteresis. Depending on which parameter is more sensitive, we may be able to deduce in which cluster the system lies, predict the sensitivity signature, and use this information for further optimization.

#### Parametric sensitivity analysis of synthetic heterogeneity modules

**Figure 7:**
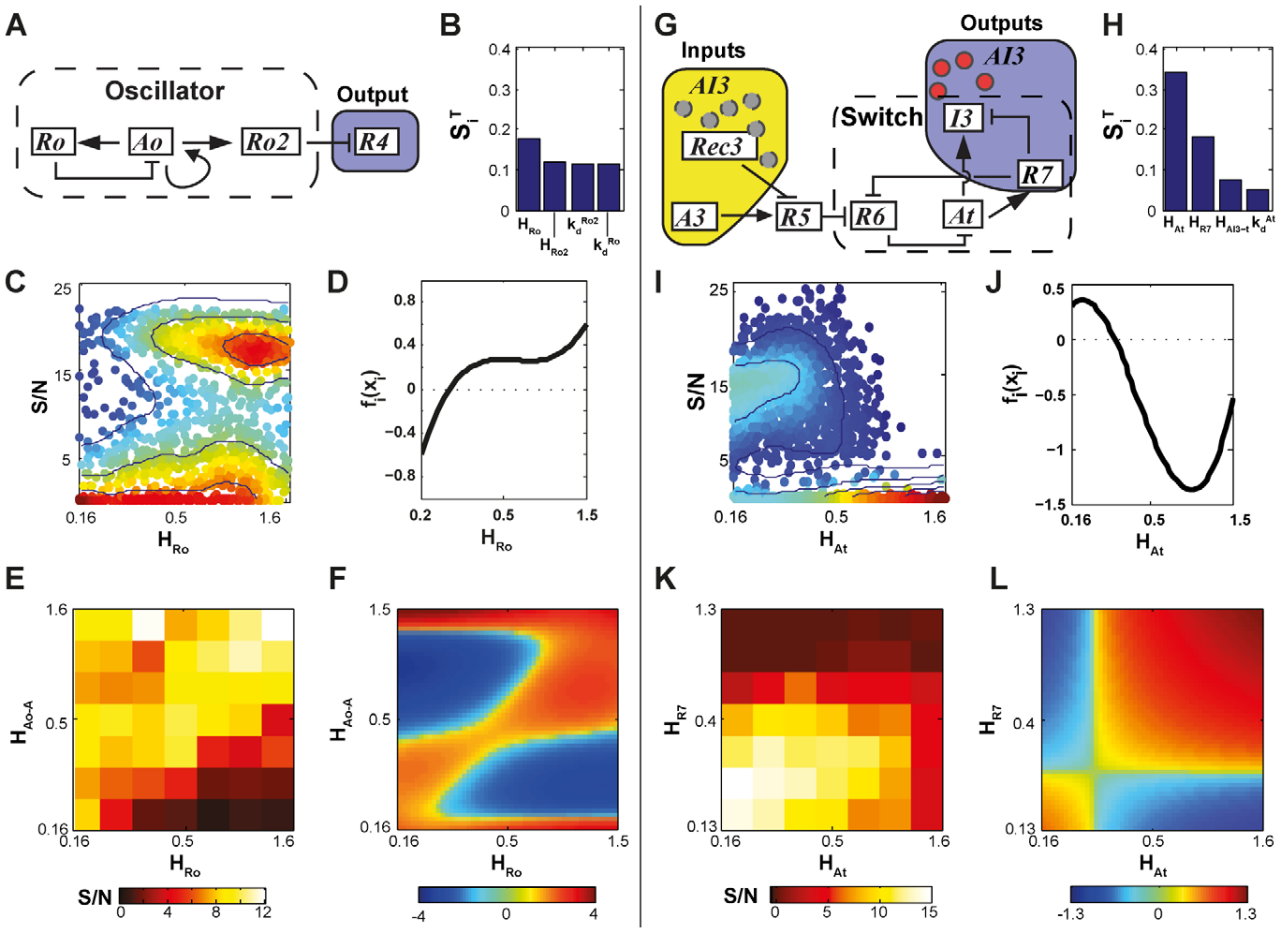
Parametric sensitivity analysis. (A,G) Circuit diagrams of the genetic components considered in (A) oscillator and (G) throttle optimization. (B,H) The most significant RS-HDMR sensitivity indices, 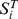, for parametric variations of the oscillator and throttle, respectively. (C,I) Observed S/N values as a function of randomly sampled rate constant values. Around 2000 different parameter sets were tested, with all oscillator or throttle parameters simultaneously varied. Each point represents an individual parameter set. Warmer colors and contour lines indicate higher point density. (D,J) Inferred first-order RS-HDMR functions describing S/N as a function of the parameters sampled in *C* and *I*. (E,K) Heat map of the S/N values against the parameters resulting from the 2000 parameter sets tested in *C* and *I*. (F,L) RS-HDMR second-order functions describing the cooperative effects between rate constants, corresponding to *E* and *K*. Second-order RS-HDMR functions capture remaining variance after the first-order functions (see *D* and *J*) have been subtracted from the data.

The impact of the oscillator and throttle modules on the performance of Systems 3 and 4 presents a particular challenge to understand and analyze (Figure 7A,G). As the two principle modules for generating synthetic heterogeneity, their ideal operating characteristics are complex and non-intuitive. Additionally, their non-trivial dynamics imply highly sensitive dependence on intramodular rate constants. As a first step to understand how to optimize these modules, we used RS-HDMR to investigate the sensitivity of S/N to random perturbations of the oscillator's and throttle's individual rate constants (Text S1, Sec. 5.2.4). As expected, results suggested highly complex and cooperative interactions among intramodular parameters, and no single parameter wholly determined system performance for either module (Figures 7, S7 and S8). Nonetheless, several parameters stood out as particularly important in governing performance.

For the oscillator, RS-HDMR indicated that the threshold at which 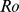 expression is activated by 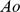 (parameter 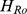) had the largest impact on system behavior (Figure 7B–D). RS-HDMR also identified cooperative relationships among oscillator rates, the most significant being between 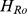 and 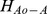 (threshold for 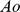 activation by itself), as shown in Figure 7E–F and S9. Although such correlations can classify ‘good’ performers (S/N 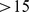) from ‘bad’ (S/N 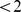) with accuracy of roughly 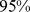 (see Text S1, Sec. 6.2), analysis of the rate constants alone insufficiently described system behavior in a quantitative manner (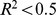, Supplementary Figure S10).

For the throttle, results indicated that the thresholds for 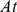 repression by 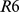 (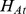) and 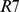 activation by 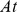 (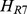) had the largest impact on system performance, and both interacted cooperatively to affect overall system performance such that low values of both parameters yielded the best S/N (Figure 7H–L and S11). As with System 3, our analysis of the rate constants alone failed to fully capture system performance in a quantitative manner (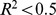, Supplementary Figure S10).

#### Phenotypic sensitivity analysis quantitatively informs system performance

Although a good first step, analysis of the module rate constants alone demonstrated two main drawbacks in this application. First, the statistical relationships between S/N and rate constants are highly convoluted and poorly captured by RS-HDMR. Second, focusing on rate constants can limit the analysis to a particularly defined network structure. To address these issues, we instead turned to analysis of high-level properties, or ‘phenotypes’, of the oscillator and throttle modules.

**Figure 8:**
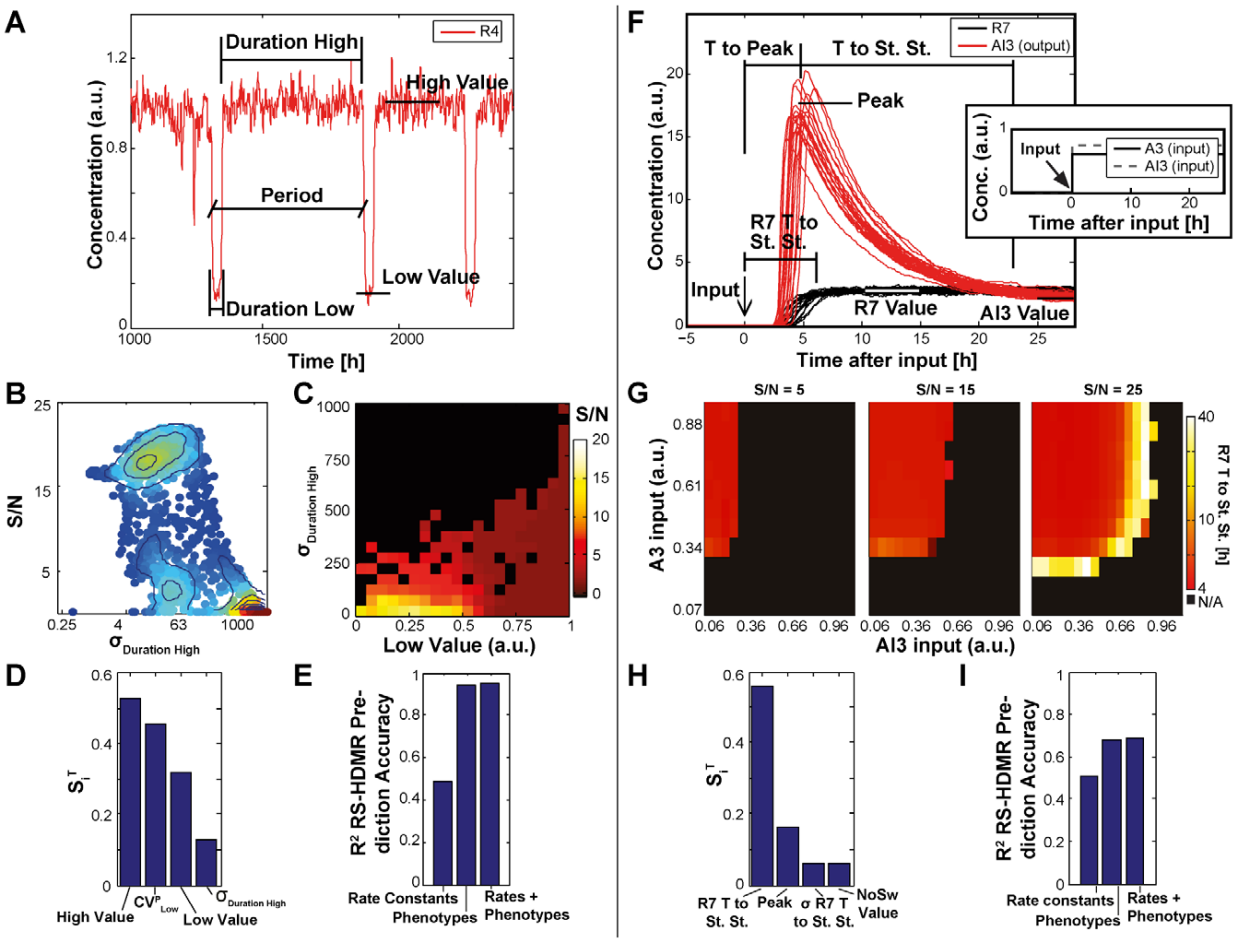
Phenotypic sensitivity analysis. (A,F) Phenotypic behavior of the oscillator (A) and throttle (F), when isolated from the full system. Roughly 2000 different sets of rate constants were tested, with all oscillator or throttle rate constants simultaneously varied. Module phenotypes were recorded for each set of rate constants. (B) Observed S/N values as a function of variance in the “duration high” of the oscillator. (C) Heat map of the S/N values against the phenotypes resulting from the random parameter sets. (G) Average ‘images’ for the phenotype *R7 T to St. St.*, observed from the random parameter sets yielding an S/N value of either 5, 15 or 25. Black represents regions where no switch occurs and no value for *R7 T to St. St.* is recorded. (D,H) The most significant RS-HDMR sensitivity indices, 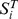, for phenotypic variations of the oscillator and throttle, respectively (see also Supplementary Table S8). (E,I) For the oscillator and throttle, respectively, RS-HDMR cross-validation predication accuracy using rate constants, phenotypes, or both.

With the oscillator, examples of phenotypes include the average period of 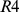 oscillations and 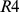 dynamic range (Figure 8A and Supplementary Table S4). As with the rate constants, correlations between oscillator phenotype and system performance are multivariate by nature (Supplementary Figure S12): for example, the relationship between 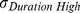 and S/N suggested a biphasic relationship, where optimal performance occurred with an intermediate level of variability (Figure 8B,C). We therefore used RS-HDMR to identify key phenotypic determinants of system performance. Interestingly, results indicated that metrics of oscillator heterogeneity (e.g., the coefficient of variation for the duration that the 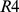 concentration is low, 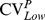, and the standard deviation for the duration that the 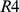 concentration is high, 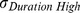), are nearly as important as the concentrations within which the modules operate (i.e., the high and low oscillator values, Figure 8D). Ultimately, RS-HDMR results suggested that module phenotypes are far more predictive of S/N than the rate constants alone (Figure 8E and S10).

For the throttle, we defined phenotypes (Supplementary Table S5) of 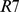 and 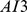 behavior as a function of the randomly-perturbed throttle rate constants. These phenotypes are more complex than those of the oscillator because the throttle module responds to two inputs, 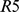 and 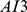 (Figure 8F). Consequently, we evaluated each throttle phenotype across combinations of both inputs, thereby producing an ‘image’ of throttle behavior over the two-input sampling space (Figure 8G and Supplementary Figure S13). For example, average images of *R7 T to St. St.* corresponding to different S/N values (Figure 8G) revealed that the best performing throttles show a clear pattern of activity: at low 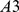 or high 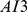, no toggle switch occurs; at high 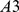 and low 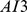, 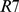 stabilizes relatively quickly; lastly, inputs lying between these two regions cause toggle switching but with much slower (and heterogeneous) 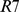 dynamics. For a more systematic approach, we used feature extraction methods from image processing along with RS-HDMR to identify key phenotypic determinants of system performance (Text S1, Sec. 5.2.4). As with the oscillator analysis, results indicated that module phenotypes predict overall system performance significantly better than rate constants alone, with the most significant phenotype being the time for 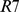 to reach steady state after receiving cues to commit (*R7 T to St. St.*, Figure 8H–I). Strikingly, RS-HDMR identified the variance with which 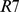 reaches steady state within this region to also be critically important for overall system performance (Supplementary Figure S8). Ultimately, phenotypic sensitivity analysis allowed for a more direct and accurate assessment of module performance compared to the analysis of rate constants alone, and did so while obviating concerns regarding the determination of rate constants that are tied to a particular system architecture (Figure 8I and S10).

#### Bayesian network analysis integrates rate constants and module phenotypes with overall system behavior

**Figure 9:**
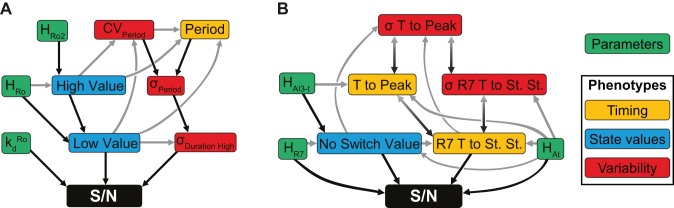
Bayesian networks of the impact of synthetic heterogeneity module phenotypes and rate constants on system performance value (S/N). (A) Bayesian network inference using oscillator rate constants and phenotypes. (B) Bayesian network inference using throttle rate constants and phenotypes. Black arrows indicate the most direct connections between a node and S/N. The Bayesian inference describes phenotype groupings relevant to state values (blue), timing (yellow), and variability (red), along with the rate constants that control these phenotypes (green).

We applied Bayesian network inference to graphically represent the strong interdependencies of the module phenotypes and their relations with the rate constants that govern them and the S/N value (Figures 9, S15, S16 and Text S1, Sec. 5.2.4). Consistent with trends seen in Figures 8D and 8H, Bayesian network inference revealed that in general, module phenotypes more directly relate to overall system performance, and the effect of rate constants on overall S/N can be described in terms of their influence on the module phenotypic behavior. Nonetheless, in some cases the module phenotypes failed to adequately capture a rate constant's influence. For example, in the oscillator this led to a direct connection between the decay rate of the oscillator's repressor, 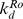, and overall S/N. Remarkably Bayesian inference identified significant upstream effectors of S/N similar to those identified by RS-HDMR, while also suggesting a hierarchy of conditional dependencies (Figure 9). Multi-parent interactions identified by Bayesian networks supported RS-HDMR results; for example, the standard deviation of the time during which the oscillator is high (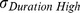) and the oscillator's *Low Value* showed significant cooperative interaction in both analyses (Figures 8C and 9A). Bayesian inference of the throttle relationships also agreed with RS-HDMR results, for example confirming relaxation kinetics (e.g., *R7 T to St. St.*) to be a significant influence on S/N, along with descriptors of its variability (Figures 8G–H and 9 B).

The integration of module phenotypes with the underlying rate constants ultimately allowed for efficient experimental optimization. Modules are likely to be experimentally implemented and phenotypically characterized in isolation before being integrated with each other. At this stage of optimization, Bayesian analysis can predict behavioral features of the individual module that will most directly influence performance in the fully integrated system, and such analysis may guide fine-tune adjustments of those module behaviors. In System 3, for example, Bayesian inference suggested that the oscillator's low value critically determined S/N, and that the threshold at which 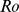 expression is activated by 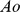 (parameter 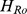) was the most direct parameter for modulating that phenotype. Although many module features and rate constants displayed covariation with overall S/N, Bayesian analysis distilled the most direct, causal influences on overall system behavior.

## Discussion

### System design and analogues from natural systems

In this work, we engineer mechanisms of robust control using synthetic generators of heterogeneity, and use a multi-faceted computational framework for design and optimization in the context of a relatively large-scale synthetic gene network. As a case study we chose tissue homeostasis control where individual cell decisions need to be coordinated to obtain desired multi-cellular behavior. To tackle this complex problem, we used top-down decomposition, achieving the overall task through the creation of interconnected modules, where each module has its own specific objective. Throughout this hierarchical optimization process we used different modeling approaches (population-based, Langevin and Gillespie simulations, see Figure 1B), while ensuring that the population-based results are consistent between the models (Supplementary Figure S17).

We designed System 1 by coupling four modules together, and simulated this system using a simplified ODE model. Computational analysis elucidated properties of global stability and demarcated regimes of steady vs. oscillatory homeostatic behavior in general tissue homeostasis systems. Analogous oscillatory homeostatic behavior from delayed feedback has been observed in natural mammalian systems, for example with hematopoiesis [40] and bacterial biofilms [41]. To mitigate the problem of population level oscillations, we created System 2 which includes a toggle switch module to implement faster feedback (Supplementary Table S9). Of note, various natural cell types regulate proliferation and differentiation by a switch similar in principle to that used in our system [42]. Analysis of System 2 using a stochastic Langevin model revealed how population-wide communication signals can be highly destabilizing to homeostasis, leading us to two new system designs. For Systems 3 and 4, the addition of the oscillator or the throttle module, respectively, provides more robust performance compared to System 2 (Figure 5) because these systems are less dependent on precise parameter values and are able to maintain sufficient population heterogeneity at lower levels of intrinsic molecular noise (Supplementary Table S9). Alternative mechanisms for generating population heterogeneity may exist. For example, the AND gate in System 2 could have been coupled with endogenously heterogeneous biological behavior such as Nanog expression (discussed below) [43]. Nonetheless, we chose to focus on the oscillator and throttle because they do not rely on potentially unpredictable endogenous mechanisms that would complicate computational modeling, and they represent two substantially distinct mechanisms for generating heterogeneity.

The design and analysis methods developed in this work attempt to identify relationships between rate constants, module phenotypes, and overall system performance, while maintaining an appreciation for the high degree of uncertainty and incomplete system knowledge in the experimental setting. For example, relating overall system performance directly to phenomenological definitions of module behavior frees the analysis from constraints to a particular module architecture or set of rate constants. Nonetheless, when more detailed information is desired we can apply global optimization strategies to capture patterns of parametric sensitivity that remain consistent across a broad range of rate constant values. For example, our analysis of the cell-cell communication module used a detailed biochemical reaction model with a large number of unknown rate constants. This level of granularity allowed us to analyze hysteretic response, which is not possible in the more abstract models. Ultimately, we addressed uncertainty by employing a novel technique, clustered sensitivity analysis, that revealed distinct patterns of relative parametric sensitivity for hysteresis that persisted across a wide range of rate constants. Previous reports have shown that bistability and hysteretic responses exist for both natural and engineered bacterial QS systems [44], [45], and in this work such bistability drives undesired oscillations. Accordingly, we designed the population control module to avoid hysteretic response and identified specific properties affecting hysteresis in our system.

### Synthetic and natural population heterogeneity

The synthetic heterogeneity modules in our systems display complex and multivariate behaviors that depend on the cooperative influence of multiple rate constants. Since existing experimental and computational biological circuit optimization methods do not scale well with system complexity, we decomposed the analysis and optimization processes for Systems 3 and 4 by characterizing modules first in isolation and then by relating their phenotypes to the performance of the overall system. We correlated module phenotypic behaviors with overall system performance, and found several significant correlations that were non-intuitive. Similarly, we identified dependencies between particular rate constants and the ability to maintain homeostasis. While Systems 3 and 4 exhibited comparable overall performances, further analyses revealed several distinguishing strengths and weaknesses (Supplementary Table S9). For example, the oscillator in System 3 appears to insulate modules from each other, while the throttle mechanism in System 4 amplifies their coupling strength (Figure 5 C–F). Our results suggest that the oscillator may mitigate problems associated with module integration, at least with respect to matching dynamics. However, the throttle mechanism is likely to be better suited for toggle switches with slow switching times (similar to the one we report on experimentally in Text S1, Sec. 1).

At a high level, our work describes strategies to exploit stochastic effects for enhancing stability of tissue homeostasis. This concept has been recently explored in a number of reports emphasizing the role probabilistic strategies play in natural mechanisms of cell-decision processing, including differentiation [29], [46], [47]. Furthermore, attempts have been made at engineering inherently stochastic processes for functions such as enhanced cellular reprogramming into induced pluripotent stem (iPS) cells [48]. Nonetheless, to our knowledge no efforts have yet been made that combine advances in synthetic biology with an appreciation of stochastic processes to engineer homeostatic tissue from isogenic cellular populations. The asynchronous oscillator stabilizes our system by generating population heterogeneity during conditions of environmental homogeneity and exogenous perturbation. Among natural systems, recent work has highlighted the role multistable feedback systems and stochastic switching play in appropriately priming cells for differentiation [49]. For example, evidence indicates the Nanog-Sox2-Oct4 network functions in part to generate population diversity by stochastically interrupting differentiation signals. Oscillators have been described as mediating cell-decisions in other biological systems, for example with p53 and NF-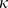
B oscillations in response to DNA damage or other stimulation. These oscillations are hypothesized to enable discrete single-cell decisions to achieve a proportionate population-wide response [50]. Intrinsic noise generated by the oscillator also affects spatiotemporal clustering in our system (Supplementary Figures S18 B,E and S19) and natural analogues of this phenomenon exist. For example, non-genetic sources of cell-cell variability can cause recently divided cells to react more similarly to pharmacological treatment [51]. Similarly, lateral inhibition as proposed in the throttle mechanism of System 4 has also been observed in biological systems, for example in pattern formation [52], segmentation [53] or in the Notch signaling pathway [54]. Consistent with these studies, our spatial simulations show strong bias towards closely spaced alternate cell types in System 4 (Supplementary Figure S18 C).

Our optimization process, as well as the different biological examples described above, aim at seemingly contradictory objectives: information has to be processed faithfully from the population control modules to a commitment signal while, at the same time, stochasticity has to be amplified to generate heterogeneity. To achieve the first objective, several of our modules exhibit digital-like behavior, allowing us to effectively match components such that downstream modules react appropriately and with relative certainty to changes in upstream module output, attenuating the effects of noise. At the same time, to generate population heterogeneity, we exploit stochasticity by amplifying its effects in nonlinear modules operating in a transient regime. As a consequence, our modules are optimized to exhibit nonlinear responses to their inputs and, depending on the objective of the module, are tuned to work far from the transition regions for robust processing of information, or near the transition region where the response is highly sensitive to stochastic effects and hence efficiently generates heterogeneity.

### Conclusions and future directions

We present here an integrated framework for forward-engineering large scale synthetic genetic circuits that combines several distinct computational approaches, and demonstrate its application to the design, analysis, and optimization of systems for controlling artificial tissue homeostasis. This framework represents a conceptual advancement for guiding experimental implementation by introducing hierarchical strategies that coordinate detailed biochemical models with modular phenotypes and optimization of module integration, all while considering parametric uncertainty and incomplete knowledge of the underlying biological context. With regard to methods development, future work may consider how to incorporate iterations of computational design with stepwise experimental implementation. Experiments could be designed to determine rate constants or high-level properties such as module phenotypes that most critically impact system performance, according to the computational modeling. Future work may also explore the limits of design automation. Network-level modeling could benefit from an integration with molecular modeling for directed optimization of molecular rate constants. Importantly, the modular design principles described in this work have been developed in part to facilitate redesign for improved performance or alternative applications. Artificial homeostasis systems have a range of potential applications in lower organisms, including co-culture systems for biosynthetic chemical production [55], controlled microbial homeostasis for environmental applications [56], and maintenance of microbial bio-sensors [57]. Medical applications may include a range of stem cell therapies currently being researched for treatment of degenerative diseases and traumatic injuries [58], [59]. Forward-engineering efforts such as those presented here may elucidate roles of heterogeneity and homeostasis in diseases such as cancer, where tumor diversity potentially contributes to chemoresistance and metastasis [60]. Beyond guiding experimental implementation of the systems described herein, we believe the design principles and control motifs revealed by our analyses may offer more general insights into the role of population heterogeneity for robust behavior, with implications for both synthetic and systems biology.

## Methods

Experimental implementations of the toggle switch and the cell-cell communication receiver were performed using immortalized human embryonic kidney cells (HEK293FT; Invitrogen), further discussed in the Text S1, Sec. 1. Computational methods and models utilized a variety of software platforms. We examined Systems 1–2 using ODE stability analyses and simulations (described in Text S1, Sec. 2), performed in Maple (Maplesoft; Waterloo, ON, Canada) and Matlab (MathWorks; Natick, MA). Systems 2–4 were analyzed using stochastic simulations. Langevin chemical simulations [35] (Text S1, Sec. 3) were performed using custom C++ code based on the 2-stage stochastic Runge-Kutta integration method with optimized parameters as described in [61]. All equations and parameters are reported in the Text S1, Sec. 3 and Table S1, respectively. In addition to Langevin simulations, Gillespie simulations (Figure 6, Text S1, Sec. 4) were implemented for Systems 2–3 using a standard rate-equation approach and the Gibson-modified Gillespie algorithm [62]. Transition rates were chosen to match the dynamics of the Langevin implementations (Table S3). For both the Langevin and Gillespie simulations, systems were described using a previously reported multicellular spatiotemporal simulation environment [15], [63]. The simulation platform (written in C++) tracks the temporal evolution of intracellular reactions within individual cells that grow and die on a 2D grid. Furthermore, the platform monitors the spatiotemporal evolution of the cells themselves and extracellular signaling molecules that diffuse among them (Text S1, Sec. 2 and 4). We utilized a two-compartment ODE model of the UPC module for the GA optimizations (Text S1, Sec. 5.3 and Table S7), and implemented the GA in C++ using a distributed computing cluster (n = 40 processor nodes). RS-HDMR (Text S1, Sec. 5.1) was implemented as reported elsewhere [37], [64]. A version of RS-HDMR [64] can be found online at http://www.aerodyne.com (free for academic users). Partial least squares regression and support vector machine classification (Text S1, Sec. 6.2) were implemented using standard Matlab functions, and Bayesian network inference (Text S1, Sec. 5.2.4) was performed in Matlab using previously described software [65].

## Supporting Information

Figure S1
**Experimental design and implementation for the signaling receiver and the toggle switch in mammalian cells.** (A) 3OC6HSL mammalian receiver circuit design: Lux activator is co-expressed with a red fluorescent protein. Addition of 3OC6HSL induces EGFP expression. (B) Dose-response of 293FT cells infected with receiver circuit to 3OC6HSL, as measured by FACS. (C) Toggle switch design: Tet inhibits lac, which is expressed along GFP. Lac inhibits tet expression, which is coupled to mCherry. (D) Bistability of the toggle switch for both activation and deactivation. The shaded gray areas denote incubation with 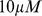 aTc. Yellow shading denotes incubation with 0.1 mM IPTG.(PDF)

Figure S2
**Simulations with feedback from all committed cells on the four-population system.** At top, heatmap shows 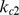 & 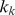 influence on 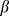-cell oscillations for System 1, with 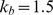, 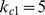, 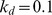 and 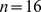. Below the heatmap are trajectories with feedback from the 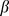-cells (left column) and all committed cells (middle column), corresponding to parameter vectors 1–3 in the heatmap. The right column shows an equivalent two-population system with stem cells (blue line) and committed cells (red lines). The approximate 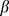-cell population was extrapolated according to Eq. S5 (see Text S1).(PDF)

Figure S3
**Nullclines of the reduced model.** (A) Nontrivial component of nullcline 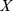 in the reduced two-population model. (B) Nullcline 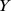 in the reduced two-population model. (C) Complete phase-plane in the reduced two-population model. (D) Nullclines for an example with three nonzero steady states in the reduced two-population model. (E) Nullcline 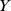 for large Hill exponents in the reduced two-population model.(PDF)

Figure S4 **Gillespie implementation of System 3.** Gillespie implementation of System 2 is identical, but without the oscillator module. Although similar, design details in the population control modules differ slightly from the Langevin implementation. Arrowed and barred connections represent transcriptional activation and repression, respectively. The dashed connection in the differentiation module represents indirect transcriptional activation.
(PDF)

Figure S5
**Parametric sampling distribution for modular time-scale analysis.** Time scale parameters were randomly and uniformly varied across one order of magnitude for the time-scale of each module or component to produce roughly 360 parameter sets for each System (2, 3, and 4). Simulations of each parameter set yielded a corresponding S/N value, which is plotted here as a function of the individual time-scale parameters. Each point represents an individual parameter set. Warmer colors indicate higher point density; contour lines also indicate point density. 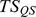 describes the time-scale of the quorum signaling molecules (including diffusion), 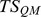 denotes the time-scale of the quorum sensing module (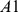, 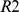, …), and other time-scales are specific to the components 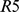, 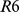, 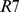 and 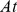.(PDF)

Figure S6
**Population level properties of time-scale optimized Systems 2, 3 and 4.** (A) Signal to noise value (S/N) for different cell volume 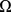. (B) Signal to noise value (S/N) for different ratio of stem cell division rate (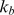) and 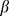-cell killing rate (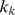). With the time-scale optimization, all systems show an increase by 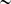
5 units of their S/N value.(PDF)

Figure S7 **Oscillator rate constants (see Table S1) were randomly varied across one order of magnitude around initial values (uniform distribution in the log space) to produce roughly 2000 parameter sets.** Simulations of each parameter set yielded a corresponding S/N value, which is plotted here as a function of the individual parameters. Each point represents an individual parameter set. Warmer colors and contour lines indicate higher point density.
(PDF)

Figure S8 **Throttle rate constants (see Table S1) were randomly varied across one order of magnitude around initial values (uniform distribution in the log space) to produce roughly 6000 parameter sets.** Simulations of each parameter set yielded a corresponding S/N value, which is plotted here as a function of the individual parameters. Each point represents an individual parameter set. Warmer colors indicate higher point density; contour lines also indicate point density.
(PDF)

Figure S9
**RS-HDMR global parametric sensitivity analysis of oscillator module rate constants (see Figure S7), describing the influence of parameter variation on observed S/N.** (A) RS-HDMR first-order component functions, in order of decreasing global sensitivity index 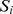. (B) Second-order RS-HDMR component functions in order of decreasing global sensitivity index 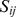.(PDF)

Figure S10
**Inference of the S/N values for Systems 3 and 4.** (A) RS-HDMR inference of System 3 S/N value using oscillator rate constants (A) or oscillator phenotypes (B), and RS-HDMR inference of System 4 S/N value using either throttle rate constants (C) or throttle phenotypes (D). The red curve indicates the distribution of S/N observed in response to parameter variation in either the oscillator or throttle. Black dots indicate observed vs. inferred S/N value for individual sets of oscillator or throttle parameter vectors. Inference accuracy corresponds to 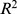 values reported in Figure 8E and 8I.(PDF)

Figure S11
**RS-HDMR parametric sensitivity analysis of the throttle module rate constants (see Figure S8), describing the influence of parameter variation on observed S/N.** (A) RS-HDMR first-order component functions, in order of decreasing sensitivity index 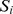. (B) Second-order RS-HDMR component functions in order of decreasing sensitivity index 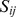.(PDF)

Figure S12 **S/N values plotted against the different oscillator phenotypes (as described in Table S4) corresponding to the parameter sets of Figure S7.** Multiple simulations of each parameter set yielded a phenotype in the isolated System (see Figure 8A) corresponding to the S/N value evaluated with the whole System 3. Each point represents an individual parameter set. Warmer colors indicate higher point density; contour lines also indicate point density.
(PDF)

Figure S13
**Standard deviation of the time for** 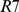 **to reach steady state in the throttle module.** (A) The standard deviation of the time for 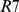 to reach its steady state is measured for given levels of 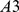 and external 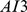; the colorbar denotes the standard deviation for 100 independent simulations. (B) Time trajectories for different combinations of 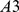 and 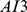: (1) the intermediate case exhibits high variability with switching behavior; (2) high 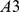 and low 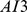 results in rapid and simultaneous toggle switching; (3) high 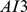 and 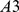 results in no toggle switching (notice the different scale on the y-axis). Input 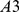 and 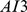 doses are introduced into the system at time 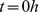 as marked by the arrow.(PDF)

Figure S14 **Average heat map for different values of S/N for the throttle phenotypes (as described in Table S5).** These maps are obtained as the average of the maps resulting from simulations of parameter sets having similar S/N values.
(PDF)

Figure S15 **Scores for the edges of the Bayesian network of the oscillator module including module parameters and phenotypes (see Text S1, Sec. 5.2.4).** Only the most significant phenotypes are taken as nodes of the network. For the Figure 9 A, only edges with scores above 0.8 are shown.
(PDF)

Figure S16 **Scores for the edges of the Bayesian network of the throttle module including module parameters and phenotypes (see Text S1, Sec. 5.2.4).** Only the most significant phenotypes are taken as nodes of the network. For the Figure 9 B, only edges with scores above 0.3 are shown.
(PDF)

Figure S17
**Population density for different ratios of division and killing rate.** Deterministic simulation with a two-population model (A,C) and stochastic simulations of the Systems 2, 3 and 4 (B–D) show qualitatively similar results. (A–B) The population of uncommitted cells remains constant with a small decrease for low rate ratio. (C–D) The population of committed cells follows a power law with an exponent near 1 for low ratio and close to 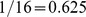 for large ratio. Power laws in (D) are fitted on the results of System 2, the closest to the ODE model.(PDF)

Figure S18
**Spatial patterning and impact of molecular noise on the patterning.** For a given uncommitted (blue) or committed (red) reference cell, the Z-score (see Text S1, Sec. 5.4) indicates the distribution bias of committed neighbors at a given distance (dashed lines, p
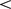
0.01). We performed simulations using the Langevin models with 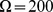 (A–C) or 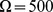 (D–F). For Systems 2 and 4, committed cells are not likely to have committed neighbors (A,C), whereas System 3 has no significant bias for short distances. With lower noise (D,E), committed cells in Systems 2 and 3 tend to cluster, such that committed cells bias to have committed cell neighbors. (F) System 4 demonstrates enhanced lateral inhibition, and committed cells bias to not have committed cell neighbors.(PDF)

Figure S19
**Spatiotemporal analysis of System 3 using the Gillespie model.** We define activity for the “Population Control” (PC) module as the level of Rec2.AI2 complex-bound promoter for the R5 repressor (*pR5.Rec2.AI2*). (A) The thick lines represent the PC activity for uncommitted cells as a function of distance from uncommitted (blue) and committed (red) neighboring cells, averaged over all cells and all time points for a given simulation. Thin lines represent PC activity +/− the standard error of the mean at each distance. (B) Average PC activity for all uncommitted cells over all time points for a given simulation are shown as a function of the number of committed neighbors at one (ordinate) and two (abscissa) grid units away. (C) We measured the time difference between nearest oscillation peaks of dimerized R1 (R1D) for all pairs of coexistent uncommitted cells throughout a given simulation. For example, if four uncommitted cells are alive at a given time point, we would calculate the phase difference among all of the pairs of cells (six in this case). Average phase difference increases as the distance between neighboring cells increases (blue line). The lower and upper black dashed lines represent the first and third quartiles of the phase difference, respectively. Phase difference increases as a function of distance because cells closer together are more likely to have originated from the same parent cell. (D) For a given uncommitted or committed reference cell, the Z-score (see Text S1, Sec. 5.4) indicates the distribution bias of committed and uncommitted neighbors at a given distance (dashed lines, p
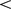
0.01). Patterning was examined for Systems 2 and 3.(PDF)

Table S1 **Parameters for the Langevin models of Systems 2 to 4.**
(PDF)

Table S2
**Scaled parameters for the time-scale analysis.** The kinetics parameters (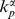 and 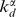) from Table S1 are scaled by the time-scale parameters 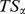 according to their module. For each combination of time-scale parameters, the 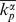 and 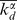 parameters are used for the Langevin simulations.(PDF)

Table S3
**List of reactions for the full multicellular model of System 3.** Depending on whether the reactions are associative or dissociative, reaction rates are in units of (molecules per 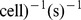 or 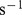.(PDF)

Table S4 **Phenotypes for the oscillator module (see** **Figure 8A** **).**
(PDF)

Table S5 **Phenotypes for the throttle module (see** **Figure 8F** **).**
(PDF)

Table S6
**Features used to analyze throttle behavior.** These features were measured for each throttle phenotype (see Table S5), where “image” refers to the observed phenotype as a response to the two inputs, 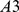 and 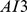 (see Figure S14).(PDF)

Table S7 **Rate constants for two-compartment model of the UPC module.**
(PDF)

Table S8
**Top RS-HDMR identified throttle features and their corresponding RS-HDMR sensitivity indices,** 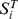 **(see** **Figure 8 H** **).**
(PDF)

Table S9 **Summary of the advantages and disadvantages of Systems 1–4.**
(PDF)

Text S1 **Supporting text.** Subsections include the following: (1) experimental proof of concept, (2) methods for the ODE modeling of Systems 1–2 and related analytical proofs, (3) methods for the Langevin modeling of Systems 1–2, (4) methods for the Gillespie modeling of Systems 2–3, (5) methods for results analyses of Systems 2–4, (6) additional results.
(PDF)
