# Supplementary figures and images for: Modular Design of Artificial Tissue Homeostasis: Robust Control through Synthetic Cellular Heterogeneity
Source: PLoS Comput Biol. 2012 Jul 19;8(7):e1002579. doi: 10.1371/journal.pcbi.1002579 (PMC3400602; doi:10.1371/journal.pcbi.1002579)

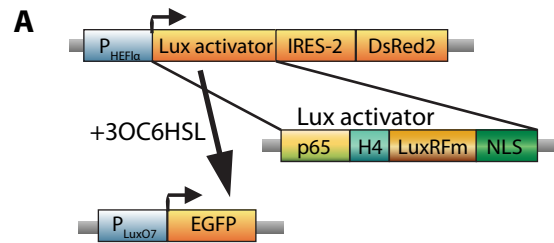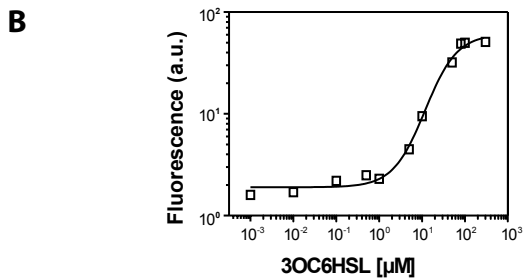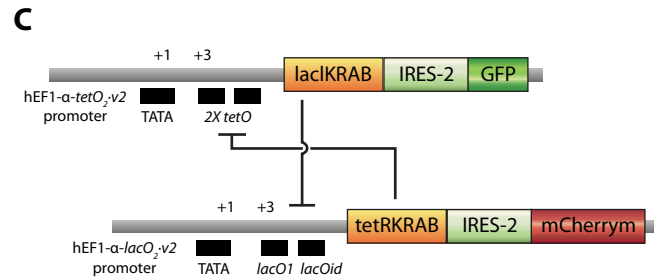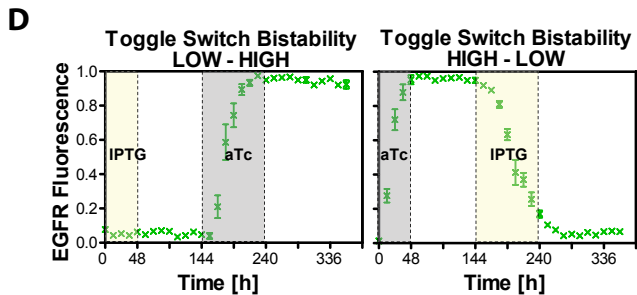

Supplement: Figure S1 — Experimental design and implementation for the signaling receiver and the toggle switch in mammalian cells. (A) 3OC6HSL mammalian receiver circuit design: Lux activator is co-expressed with a red fluorescent protein. Addition of 3OC6HSL induces EGFP expression. (B) Dose-response of 293FT cells infected with receiver circuit to 3OC6HSL, as measured by FACS. (C) Toggle switch design: Tet inhibits lac, which is expressed along GFP. Lac inhibits tet expression, which is coupled to mCherry. (D) Bistability of the toggle switch for both activation and deactivation. The shaded gray areas denote incubation with aTc. Yellow shading denotes incubation with 0.1 mM IPTG. (PDF) [file pcbi.1002579.s001.pdf]

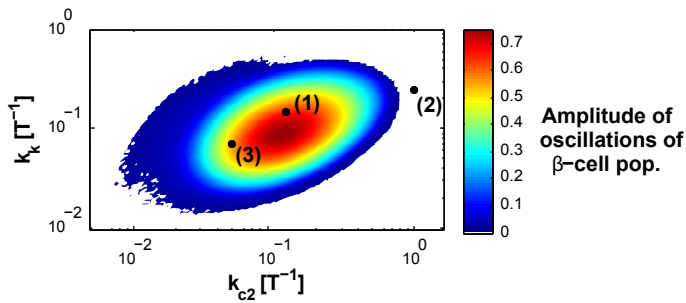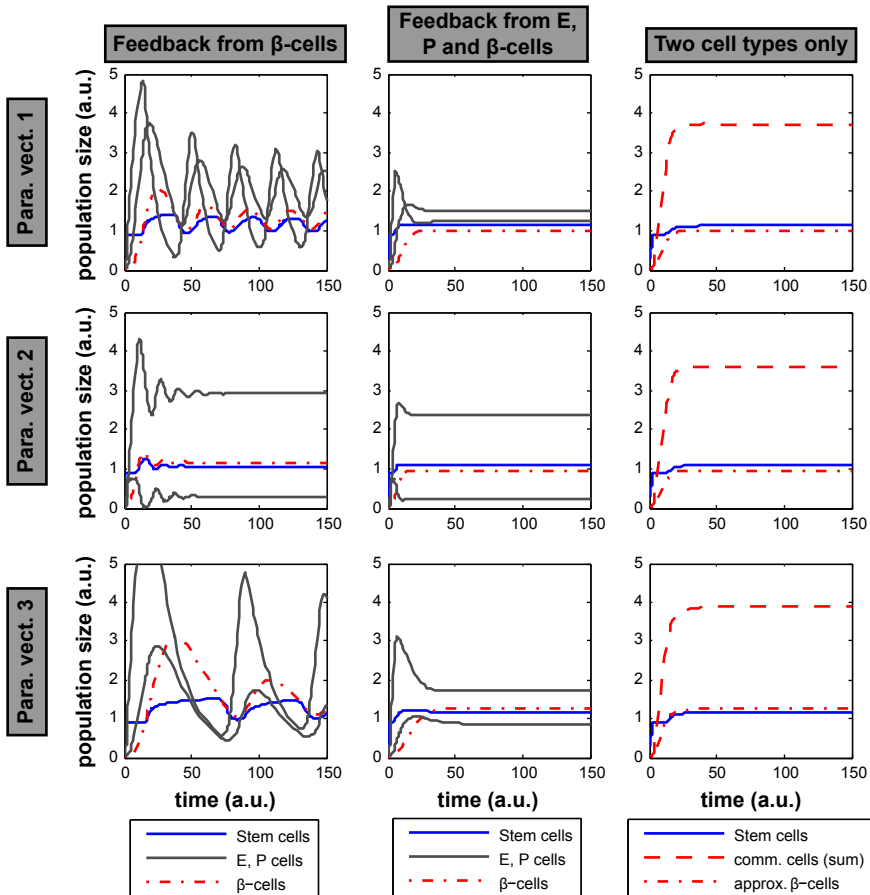

Supplement: Figure S2 — Simulations with feedback from all committed cells on the four-population system. At top, heatmap shows & influence on -cell oscillations for System 1, with , , and . Below the heatmap are trajectories with feedback from the -cells (left column) and all committed cells (middle column), corresponding to parameter vectors 1–3 in the heatmap. The right column shows an equivalent two-population system with stem cells (blue line) and committed cells (red lines). The approximate -cell population was extrapolated according to Eq. S5 (see Text S1). (PDF) [file pcbi.1002579.s002.pdf]

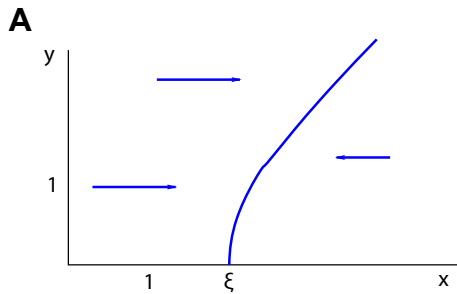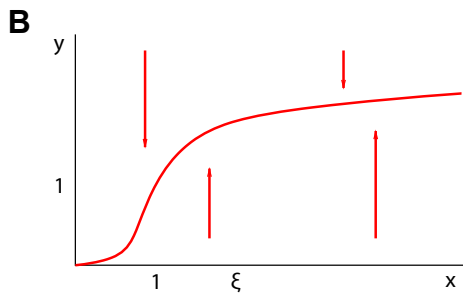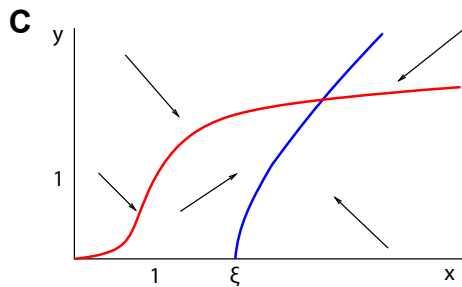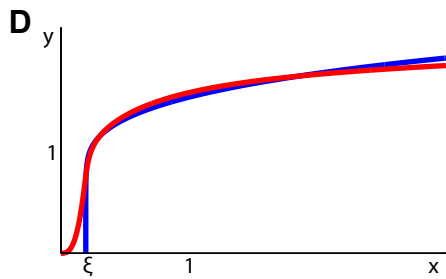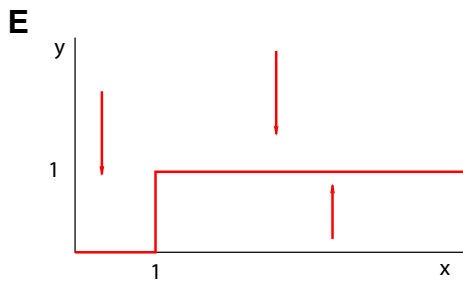

Supplement: Figure S3 — Nullclines of the reduced model. (A) Nontrivial component of nullcline in the reduced two-population model. (B) Nullcline in the reduced two-population model. (C) Complete phase-plane in the reduced two-population model. (D) Nullclines for an example with three nonzero steady states in the reduced two-population model. (E) Nullcline for large Hill exponents in the reduced two-population model. (PDF) [file pcbi.1002579.s003.pdf]

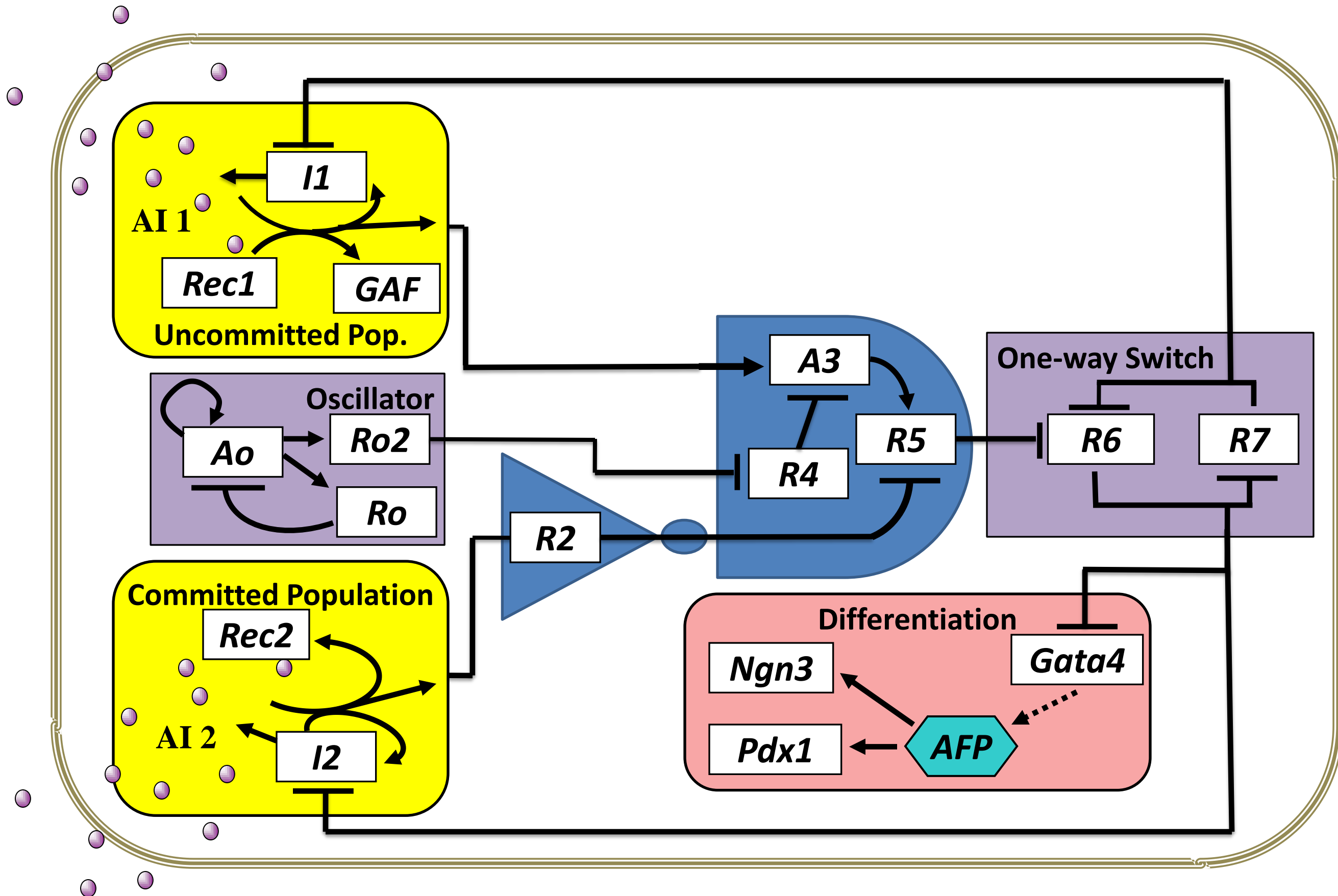

Supplement: Figure S4 — Gillespie implementation of System 3. Gillespie implementation of System 2 is identical, but without the oscillator module. Although similar, design details in the population control modules differ slightly from the Langevin implementation. Arrowed and barred connections represent transcriptional activation and repression, respectively. The dashed connection in the differentiation module represents indirect transcriptional activation. (PDF) [file pcbi.1002579.s004.pdf]

System 2

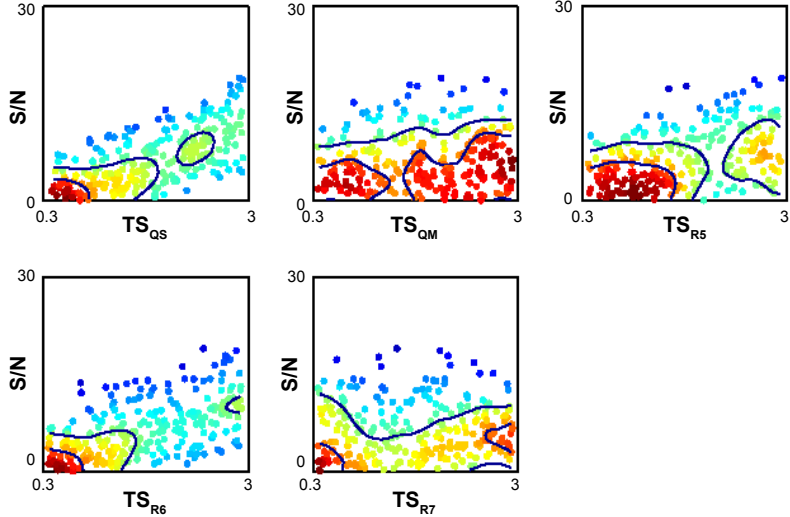

System 3

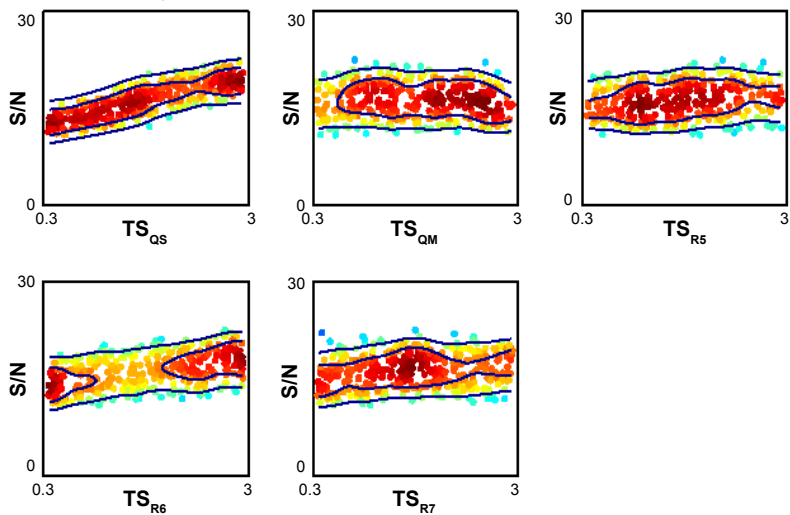

System 4

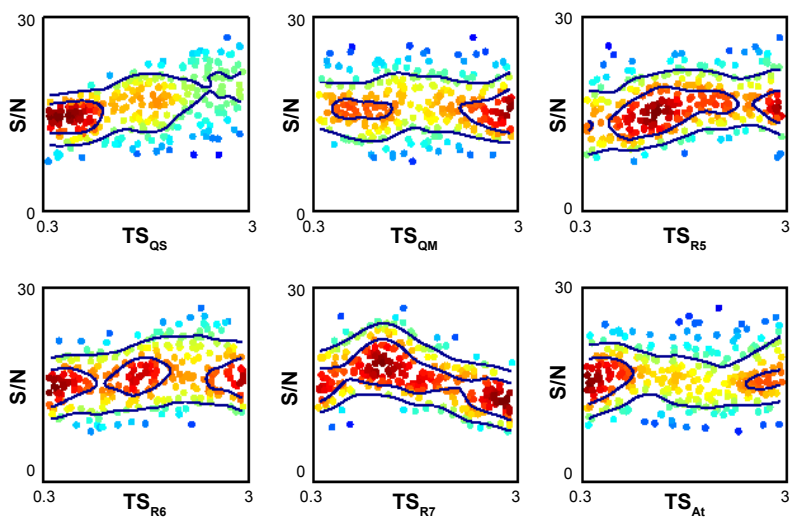

Supplement: Figure S5 — Parametric sampling distribution for modular time-scale analysis. Time scale parameters were randomly and uniformly varied across one order of magnitude for the time-scale of each module or component to produce roughly 360 parameter sets for each System (2, 3, and 4). Simulations of each parameter set yielded a corresponding S/N value, which is plotted here as a function of the individual time-scale parameters. Each point represents an individual parameter set. Warmer colors indicate higher point density; contour lines also indicate point density. describes the time-scale of the quorum signaling molecules (including diffusion), denotes the time-scale of the quorum sensing module (, , …), and other time-scales are specific to the components , , and . (PDF) [file pcbi.1002579.s005.pdf]

**A**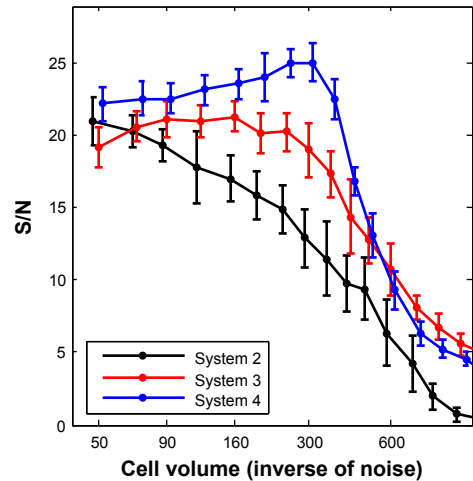**B**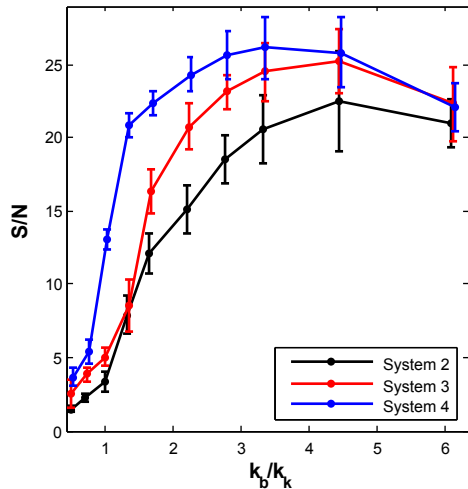

Supplement: Figure S6 — Population level properties of time-scale optimized Systems 2, 3 and 4. (A) Signal to noise value (S/N) for different cell volume . (B) Signal to noise value (S/N) for different ratio of stem cell division rate () and -cell killing rate (). With the time-scale optimization, all systems show an increase by 5 units of their S/N value. (PDF) [file pcbi.1002579.s006.pdf]

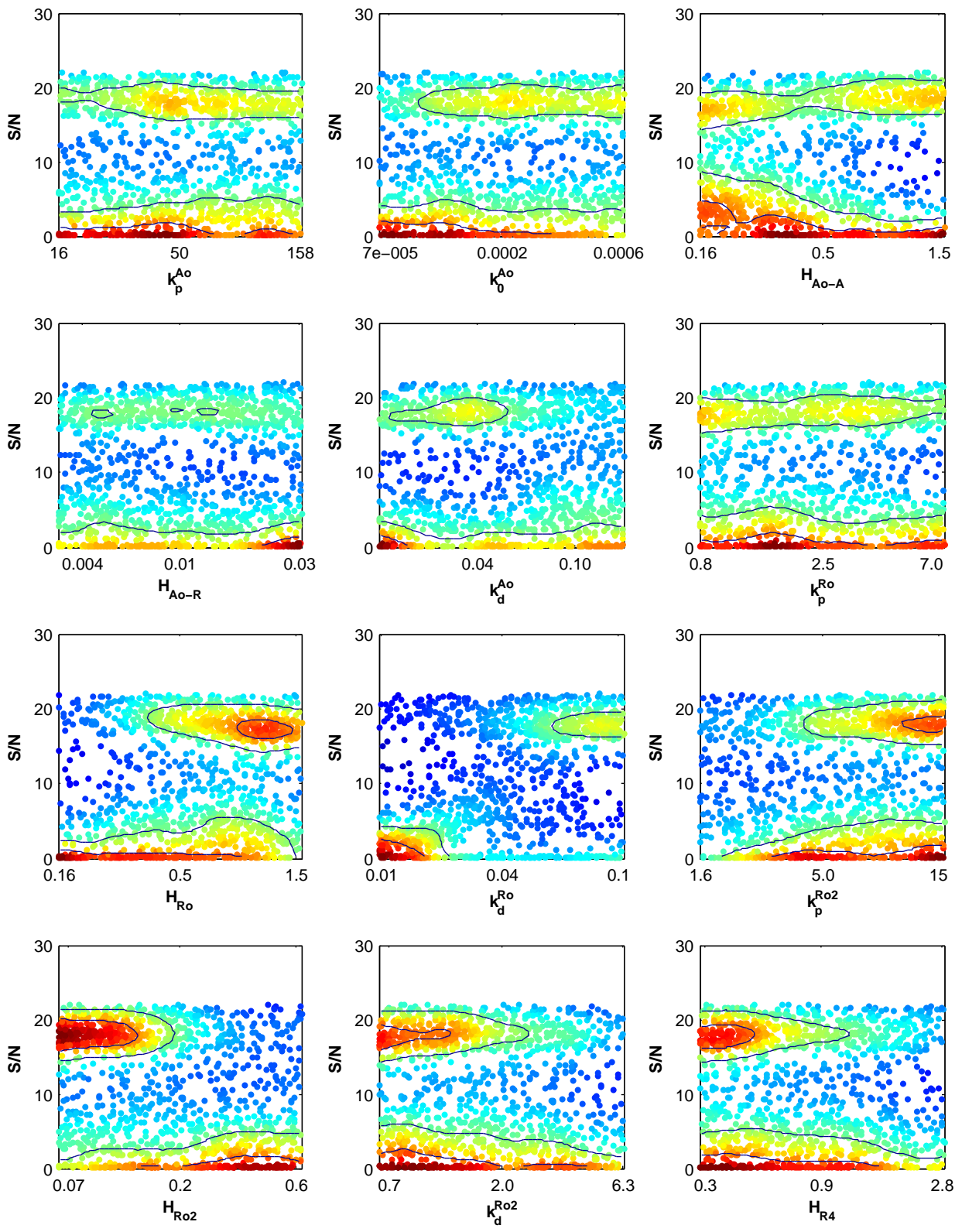

Supplement: Figure S7 — Oscillator rate constants (see Table S1) were randomly varied across one order of magnitude around initial values (uniform distribution in the log space) to produce roughly 2000 parameter sets. Simulations of each parameter set yielded a corresponding S/N value, which is plotted here as a function of the individual parameters. Each point represents an individual parameter set. Warmer colors and contour lines indicate higher point density. (PDF) [file pcbi.1002579.s007.pdf]

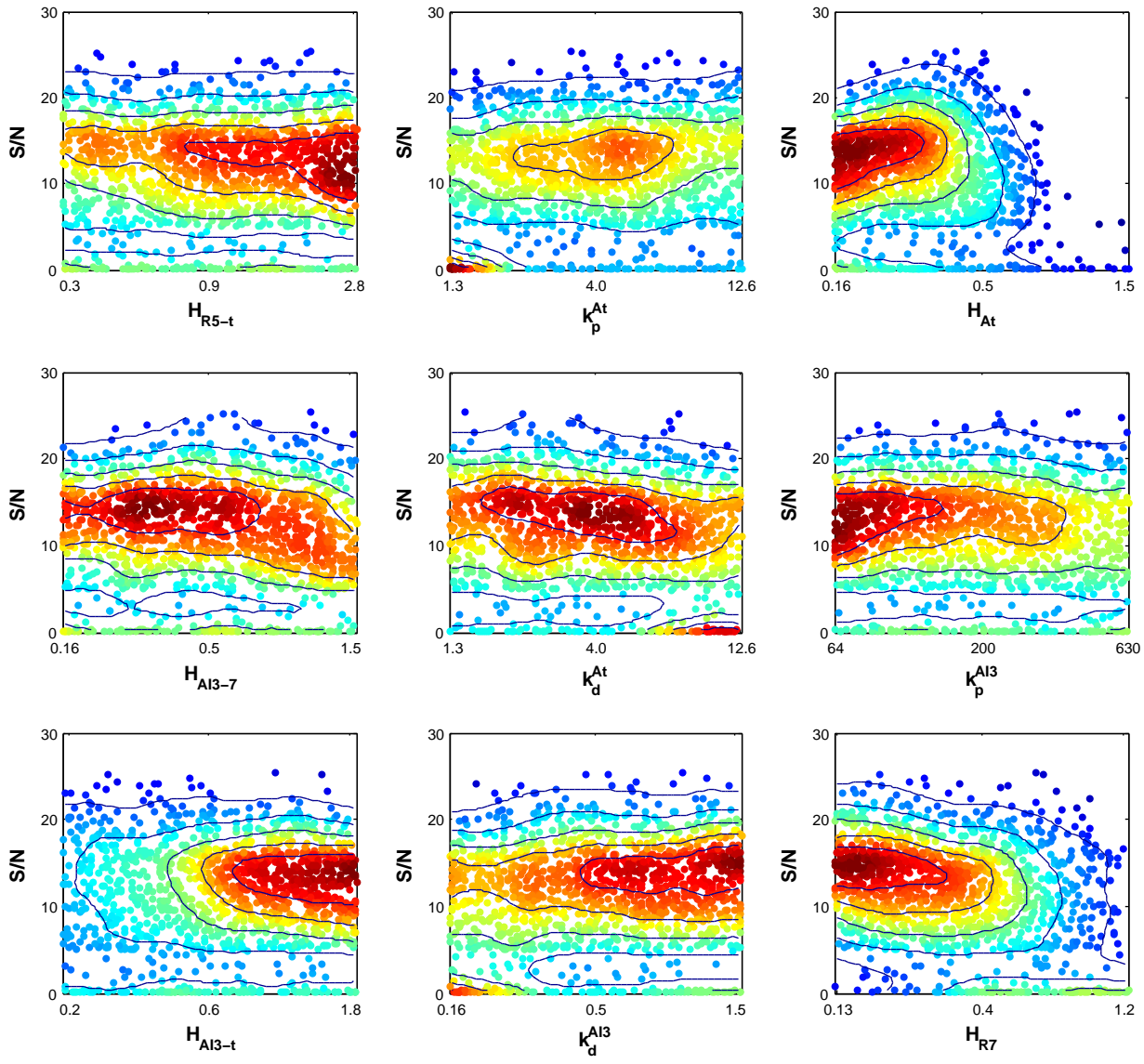

Supplement: Figure S8 — Throttle rate constants (see Table S1) were randomly varied across one order of magnitude around initial values (uniform distribution in the log space) to produce roughly 6000 parameter sets. Simulations of each parameter set yielded a corresponding S/N value, which is plotted here as a function of the individual parameters. Each point represents an individual parameter set. Warmer colors indicate higher point density; contour lines also indicate point density. (PDF) [file pcbi.1002579.s008.pdf]

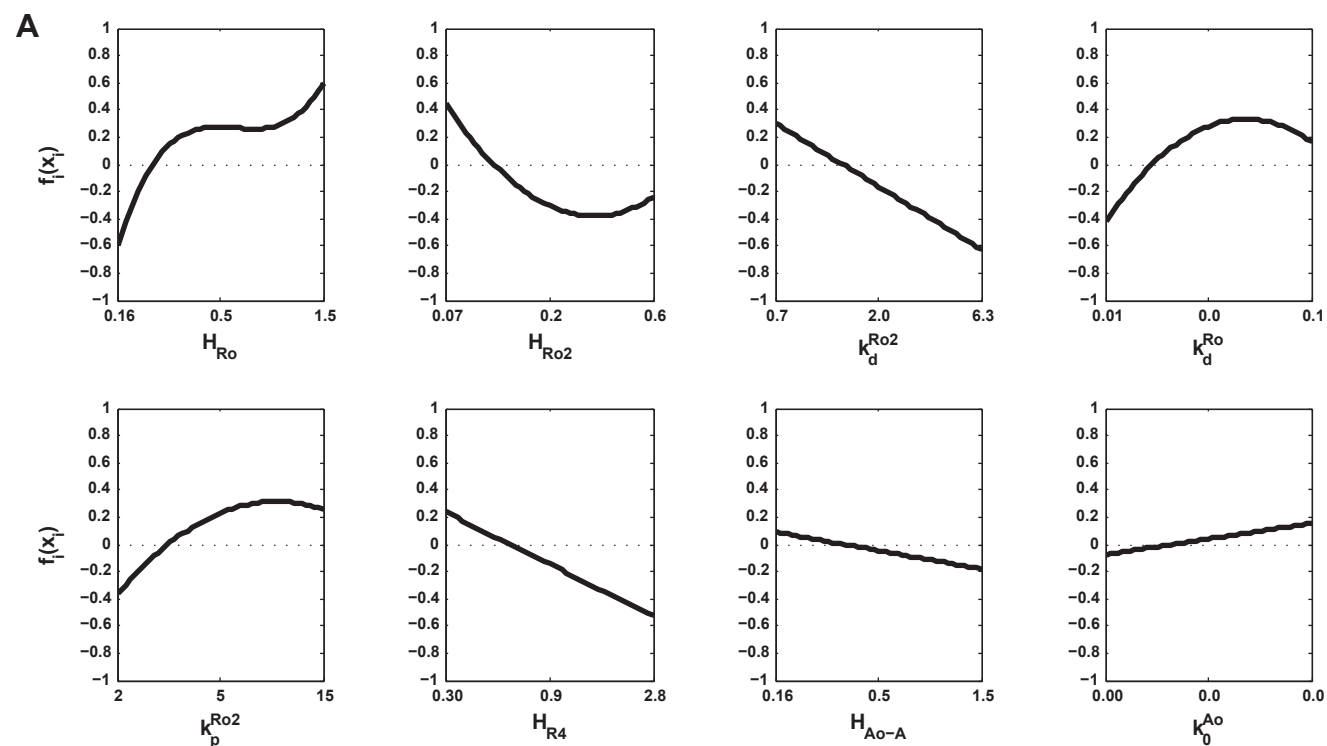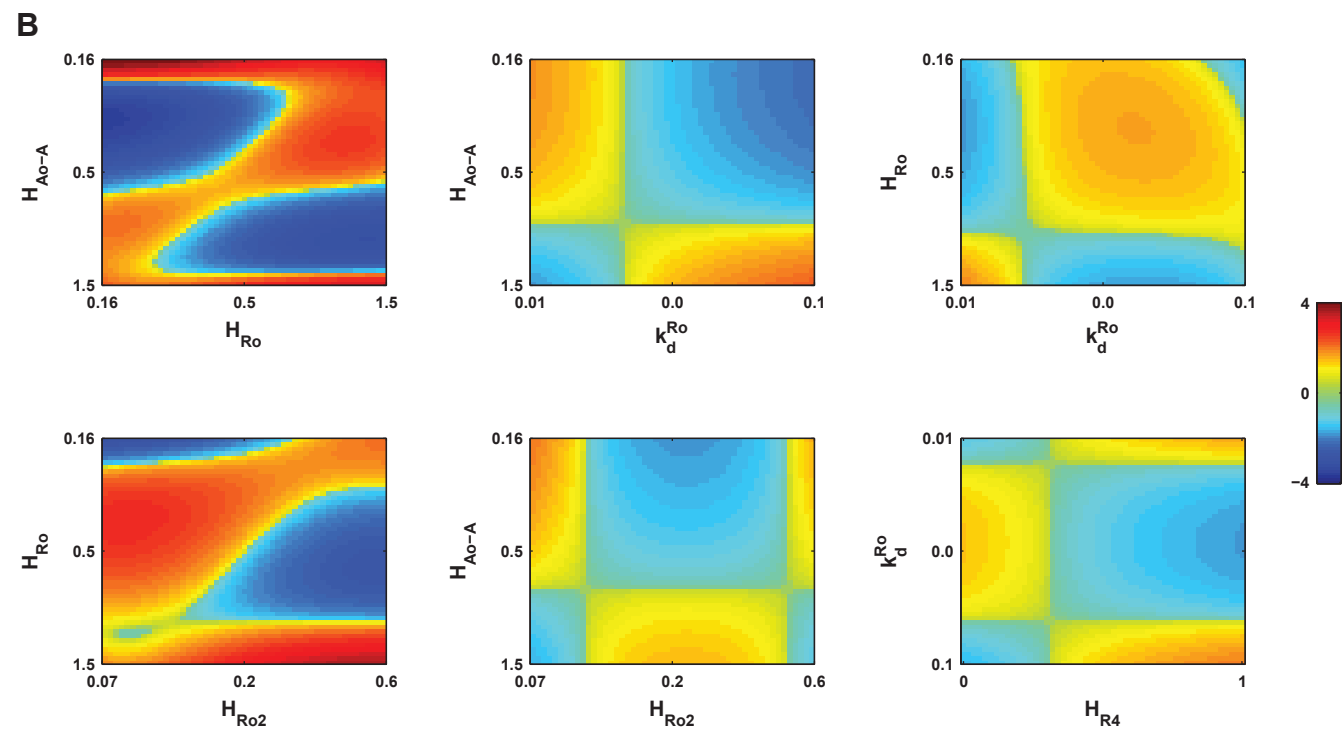

Supplement: Figure S9 — RS-HDMR global parametric sensitivity analysis of oscillator module rate constants (see Figure S7), describing the influence of parameter variation on observed S/N. (A) RS-HDMR first-order component functions, in order of decreasing global sensitivity index . (B) Second-order RS-HDMR component functions in order of decreasing global sensitivity index . (PDF) [file pcbi.1002579.s009.pdf]

**A**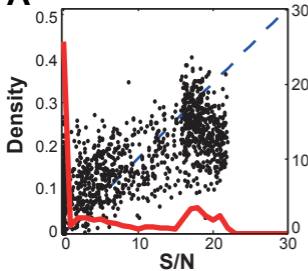**B**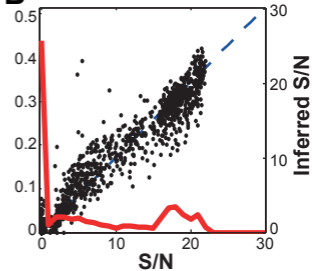**C**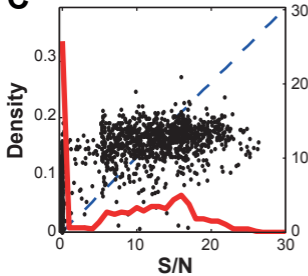**D**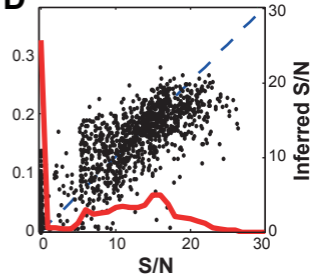

Supplement: Figure S10 — Inference of the S/N values for Systems 3 and 4. (A) RS-HDMR inference of System 3 S/N value using oscillator rate constants (A) or oscillator phenotypes (B), and RS-HDMR inference of System 4 S/N value using either throttle rate constants (C) or throttle phenotypes (D). The red curve indicates the distribution of S/N observed in response to parameter variation in either the oscillator or throttle. Black dots indicate observed vs. inferred S/N value for individual sets of oscillator or throttle parameter vectors. Inference accuracy corresponds to values reported in Figure 8E and 8I. (PDF) [file pcbi.1002579.s010.pdf]

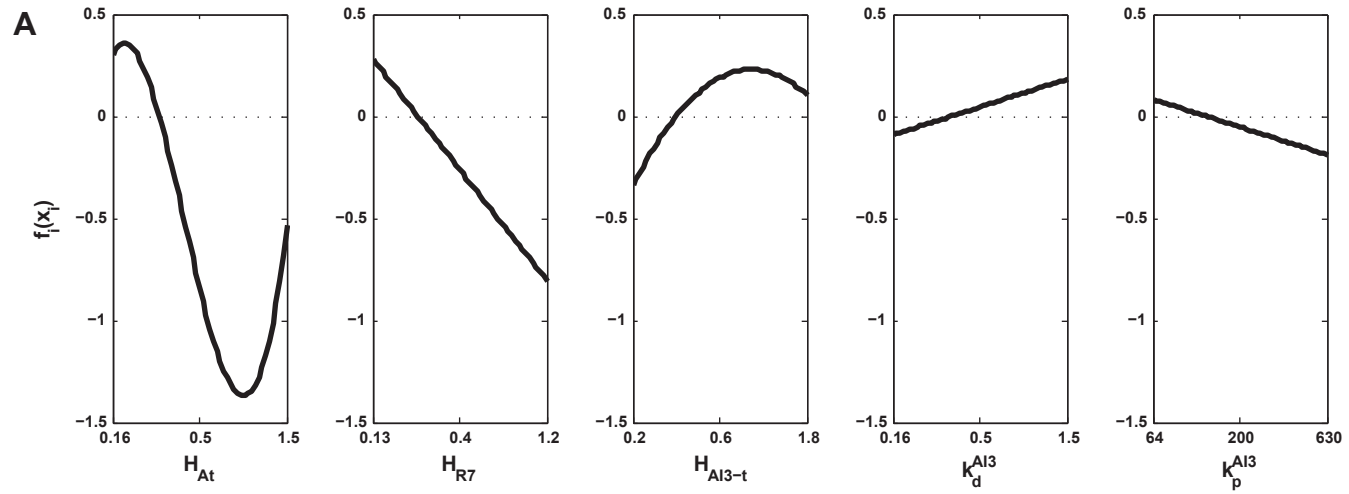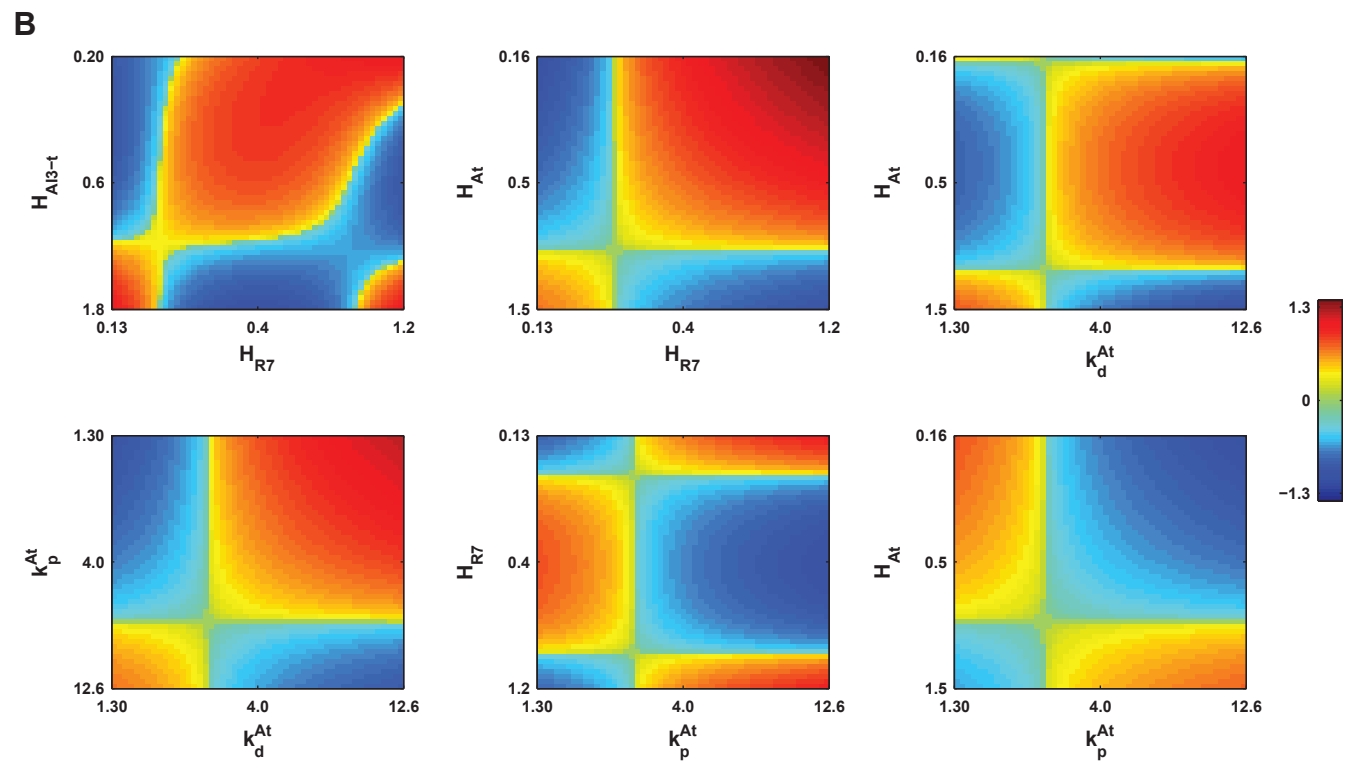

Supplement: Figure S11 — RS-HDMR parametric sensitivity analysis of the throttle module rate constants (see Figure S8), describing the influence of parameter variation on observed S/N. (A) RS-HDMR first-order component functions, in order of decreasing sensitivity index . (B) Second-order RS-HDMR component functions in order of decreasing sensitivity index . (PDF) [file pcbi.1002579.s011.pdf]

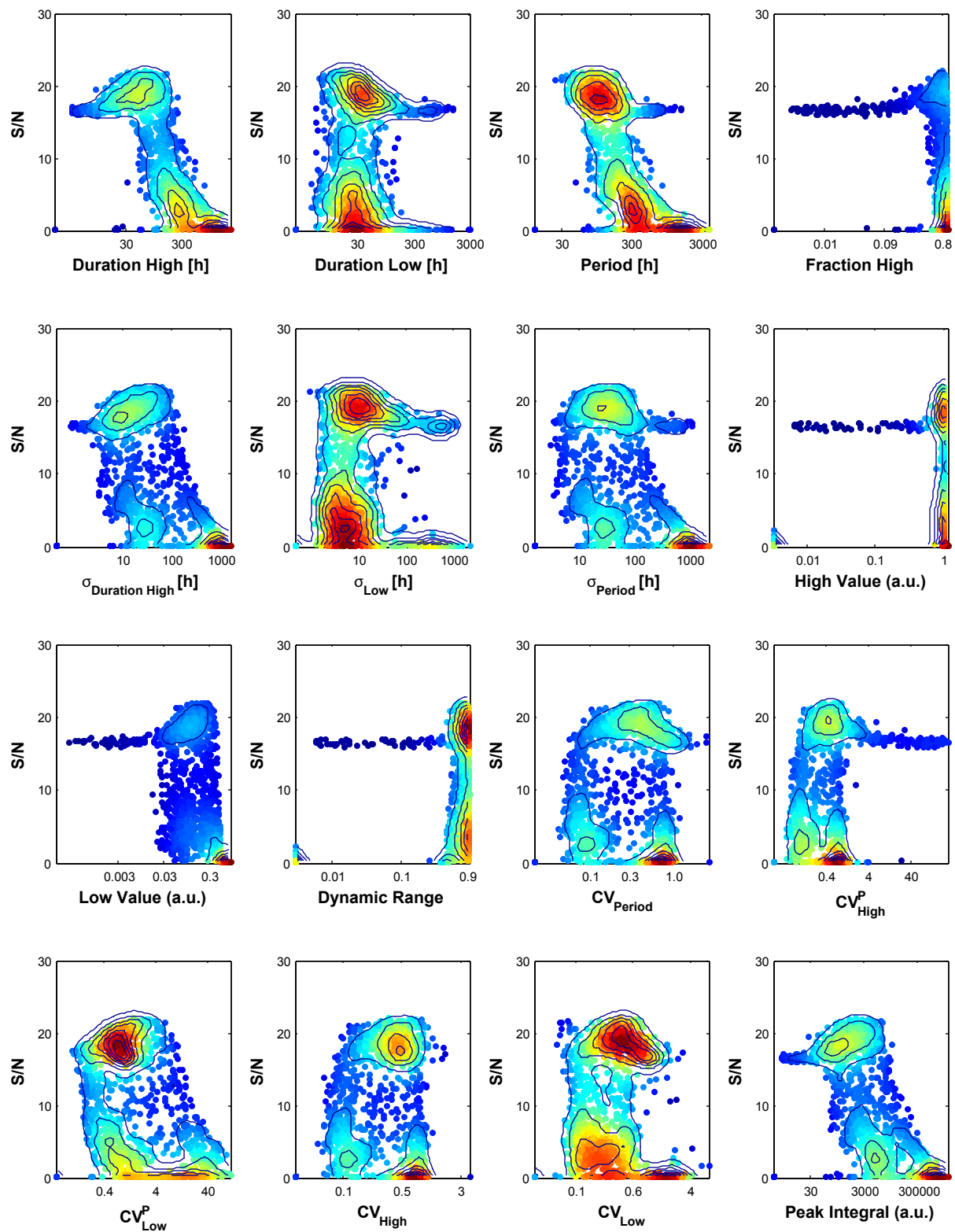

Supplement: Figure S12 — S/N values plotted against the different oscillator phenotypes (as described in Table S4) corresponding to the parameter sets of Figure S7. Multiple simulations of each parameter set yielded a phenotype in the isolated System (see Figure 8A) corresponding to the S/N value evaluated with the whole System 3. Each point represents an individual parameter set. Warmer colors indicate higher point density; contour lines also indicate point density. (PDF) [file pcbi.1002579.s012.pdf]

**A**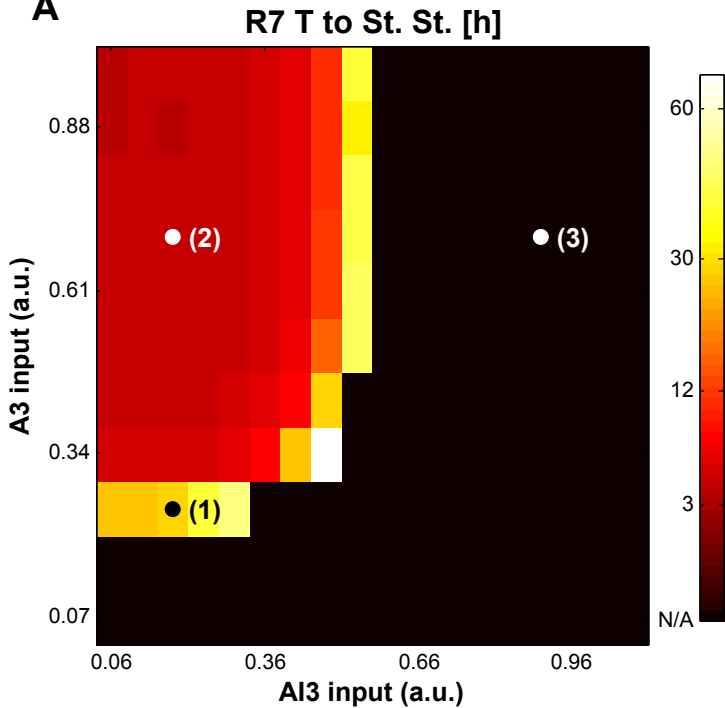**B**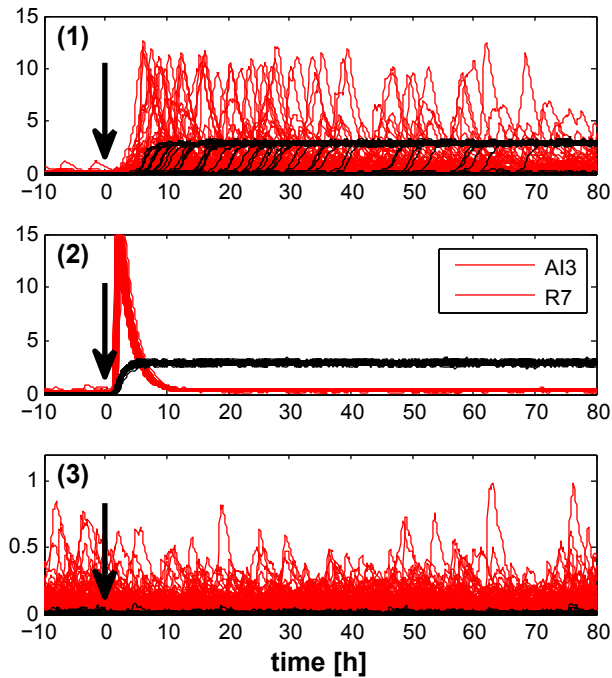

Supplement: Figure S13 — Standard deviation of the time for to reach steady state in the throttle module. (A) The standard deviation of the time for to reach its steady state is measured for given levels of and external ; the colorbar denotes the standard deviation for 100 independent simulations. (B) Time trajectories for different combinations of and : (1) the intermediate case exhibits high variability with switching behavior; (2) high and low results in rapid and simultaneous toggle switching; (3) high and results in no toggle switching (notice the different scale on the y-axis). Input and doses are introduced into the system at time as marked by the arrow. (PDF) [file pcbi.1002579.s013.pdf]

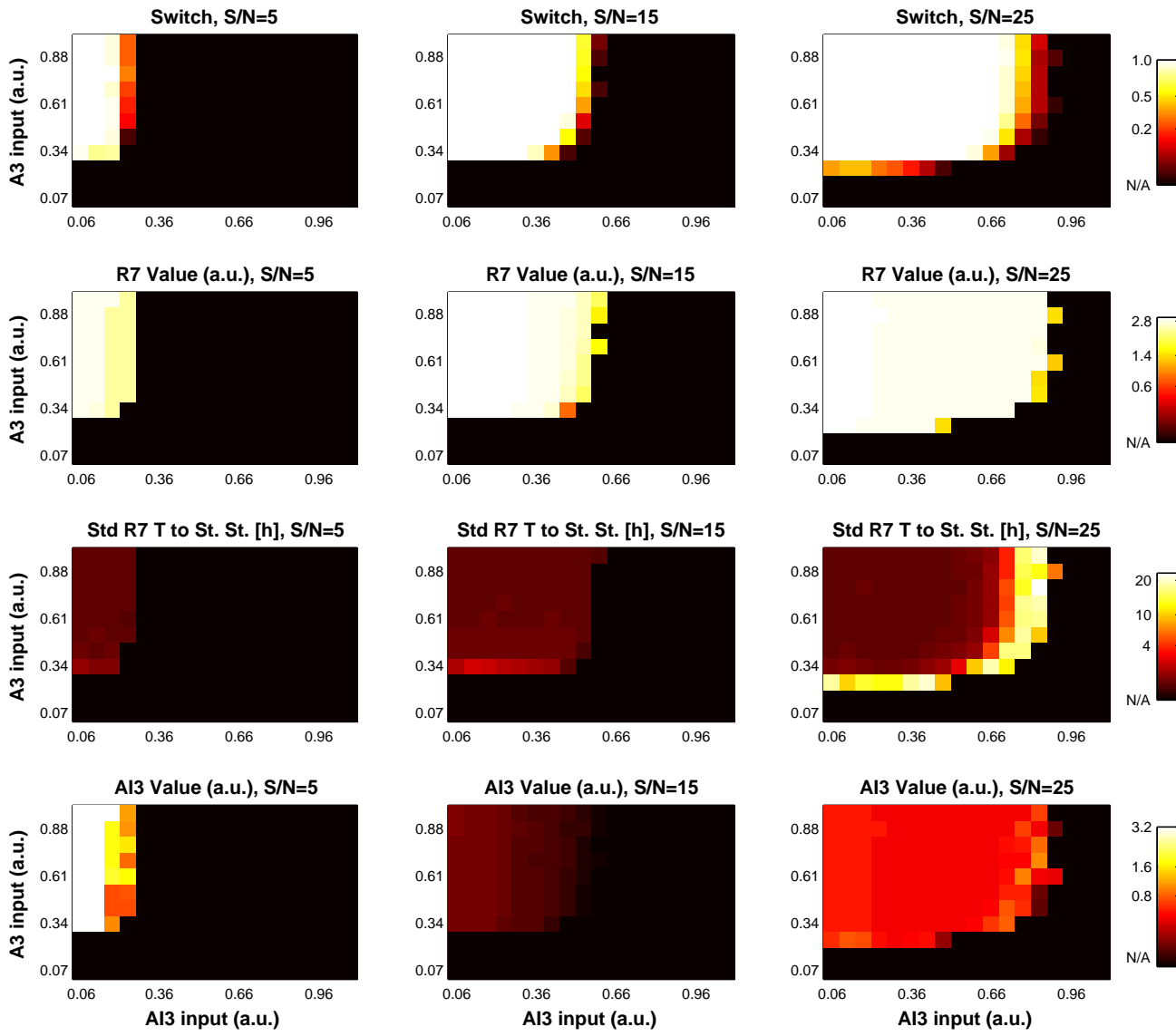

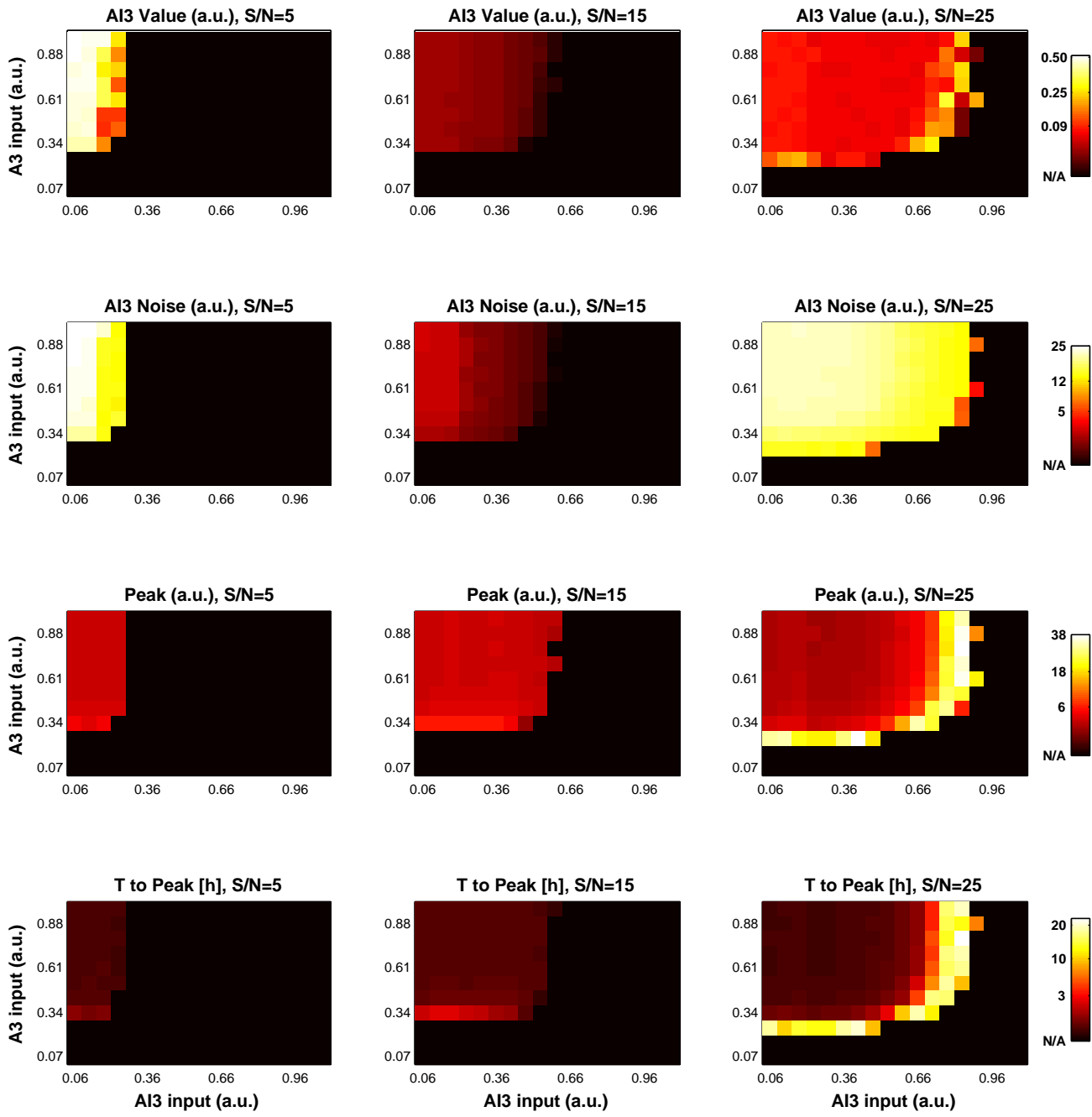

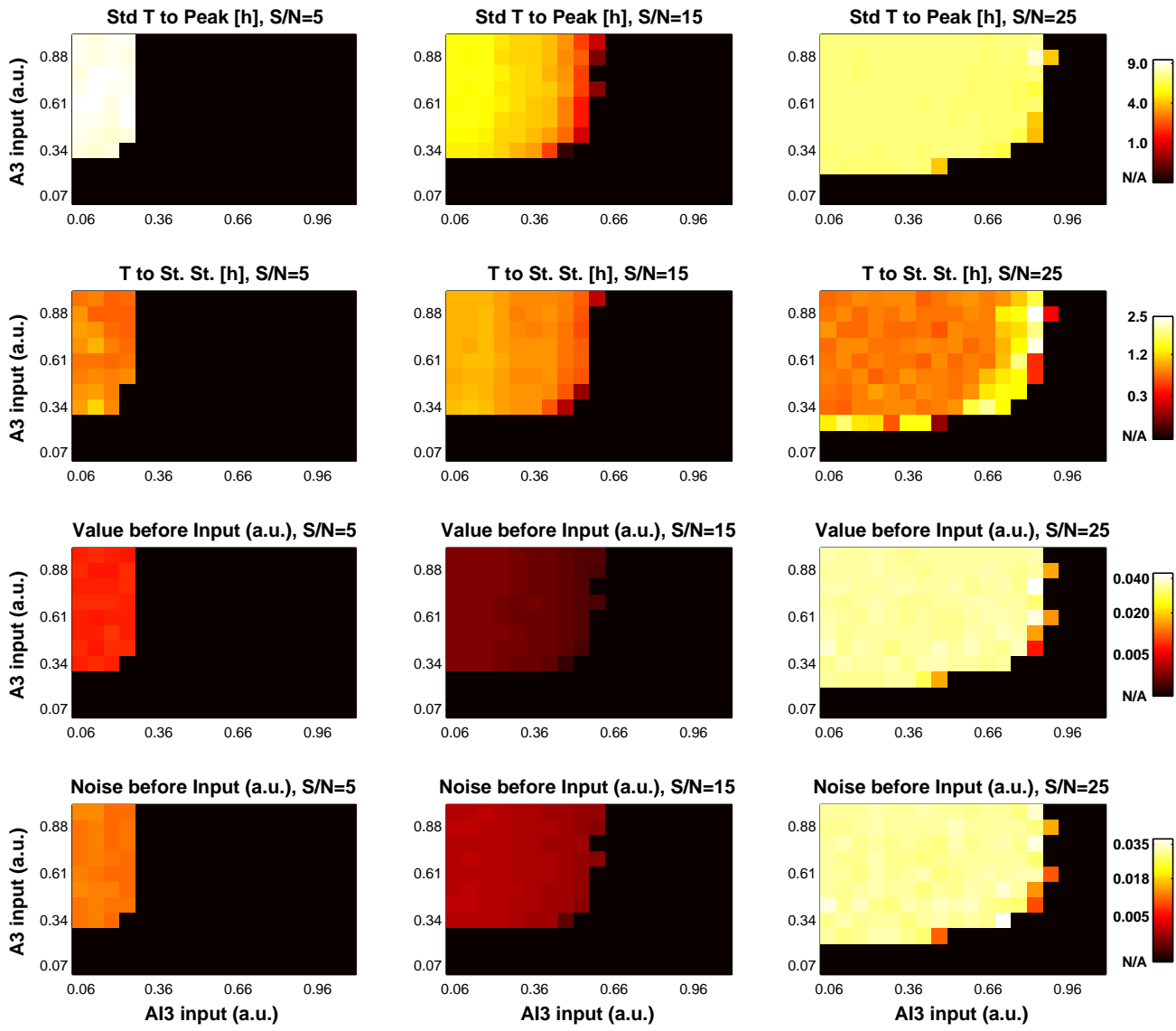

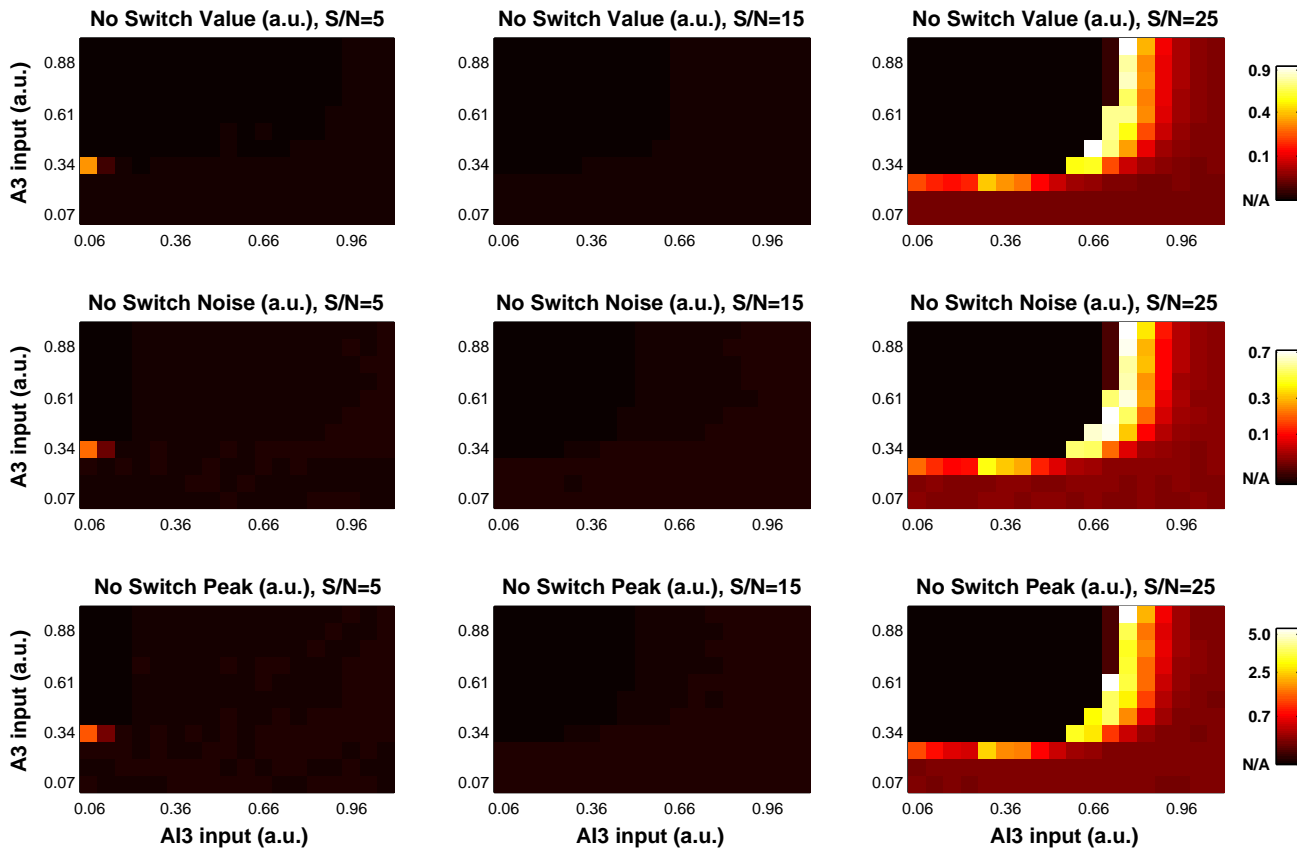

Supplement: Figure S14 — Average heat map for different values of S/N for the throttle phenotypes (as described in Table S5). These maps are obtained as the average of the maps resulting from simulations of parameter sets having similar S/N values. (PDF) [file pcbi.1002579.s014.pdf]

Upstream Node

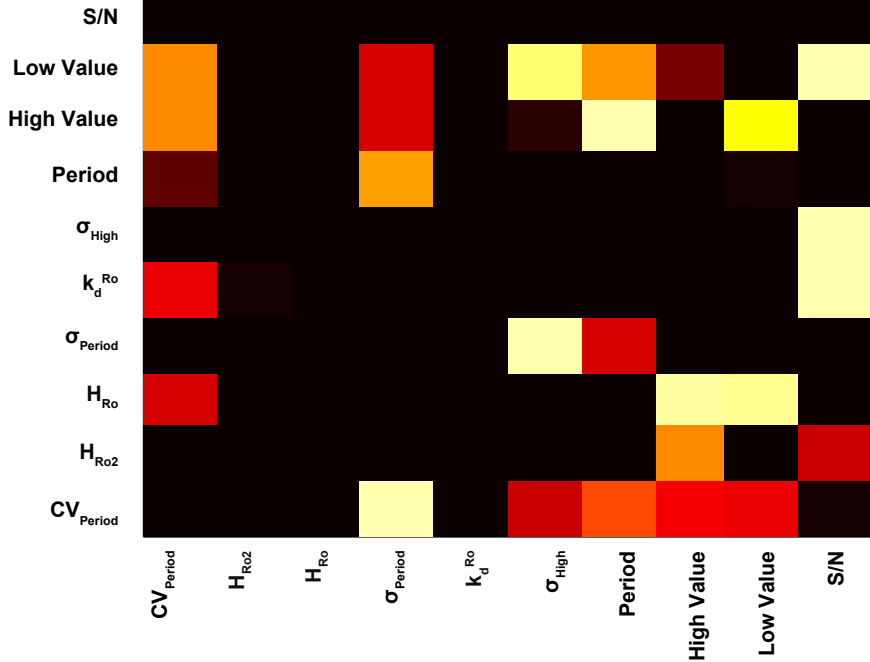

Downstream Node

Supplement: Figure S15 — Scores for the edges of the Bayesian network of the oscillator module including module parameters and phenotypes (see Text S1, Sec. 5.2.4). Only the most significant phenotypes are taken as nodes of the network. For the Figure 9 A, only edges with scores above 0.8 are shown. (PDF) [file pcbi.1002579.s015.pdf]

Upstream Node

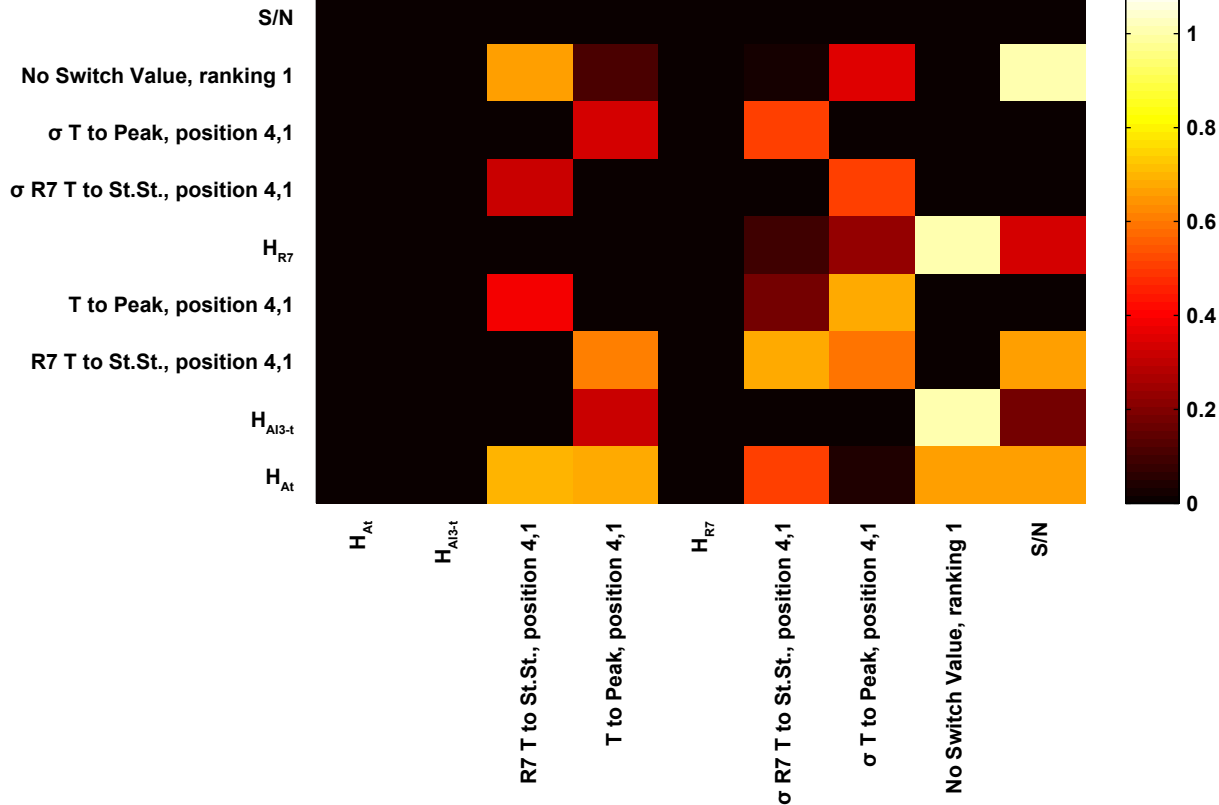

Downstream Node

Supplement: Figure S16 — Scores for the edges of the Bayesian network of the throttle module including module parameters and phenotypes (see Text S1, Sec. 5.2.4). Only the most significant phenotypes are taken as nodes of the network. For the Figure 9 B, only edges with scores above 0.3 are shown. (PDF) [file pcbi.1002579.s016.pdf]

**A****System 2**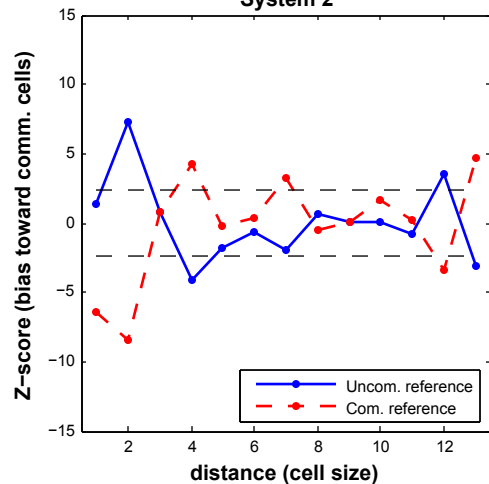**B****System 3**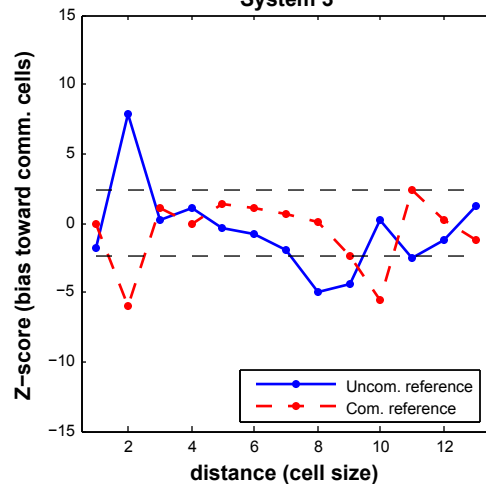**C****System 4**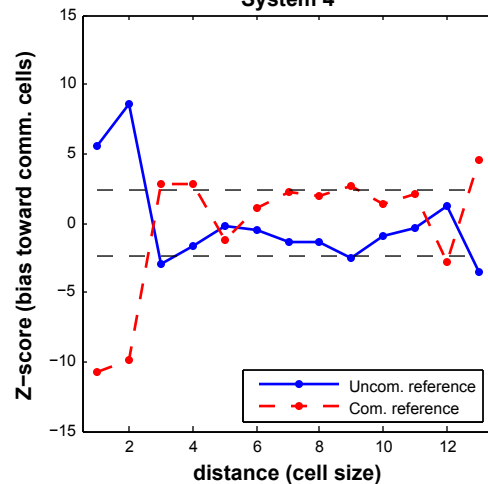**D****System 2 (low noise)**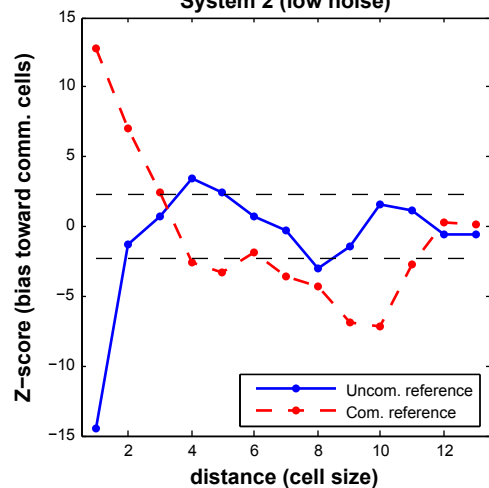**E****System 3 (low noise)**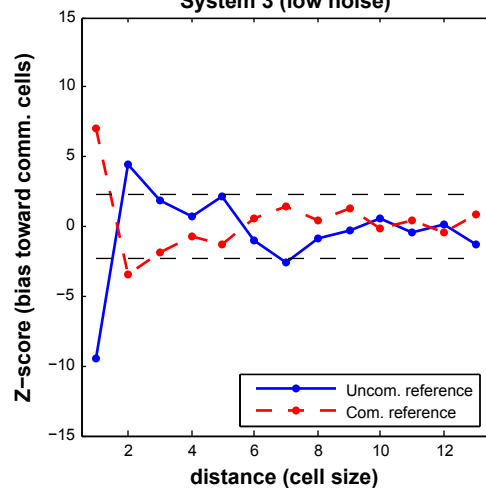**F****System 4 (low noise)**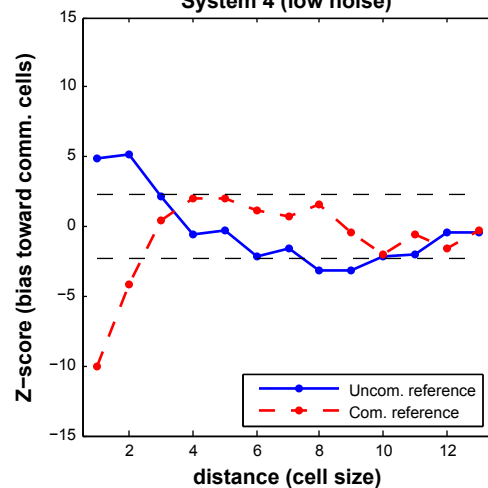

Supplement: Figure S18 — Spatial patterning and impact of molecular noise on the patterning. For a given uncommitted (blue) or committed (red) reference cell, the Z-score (see Text S1, Sec. 5.4) indicates the distribution bias of committed neighbors at a given distance (dashed lines, p0.01). We performed simulations using the Langevin models with (A–C) or (D–F). For Systems 2 and 4, committed cells are not likely to have committed neighbors (A,C), whereas System 3 has no significant bias for short distances. With lower noise (D,E), committed cells in Systems 2 and 3 tend to cluster, such that committed cells bias to have committed cell neighbors. (F) System 4 demonstrates enhanced lateral inhibition, and committed cells bias to not have committed cell neighbors. (PDF) [file pcbi.1002579.s018.pdf]

**A**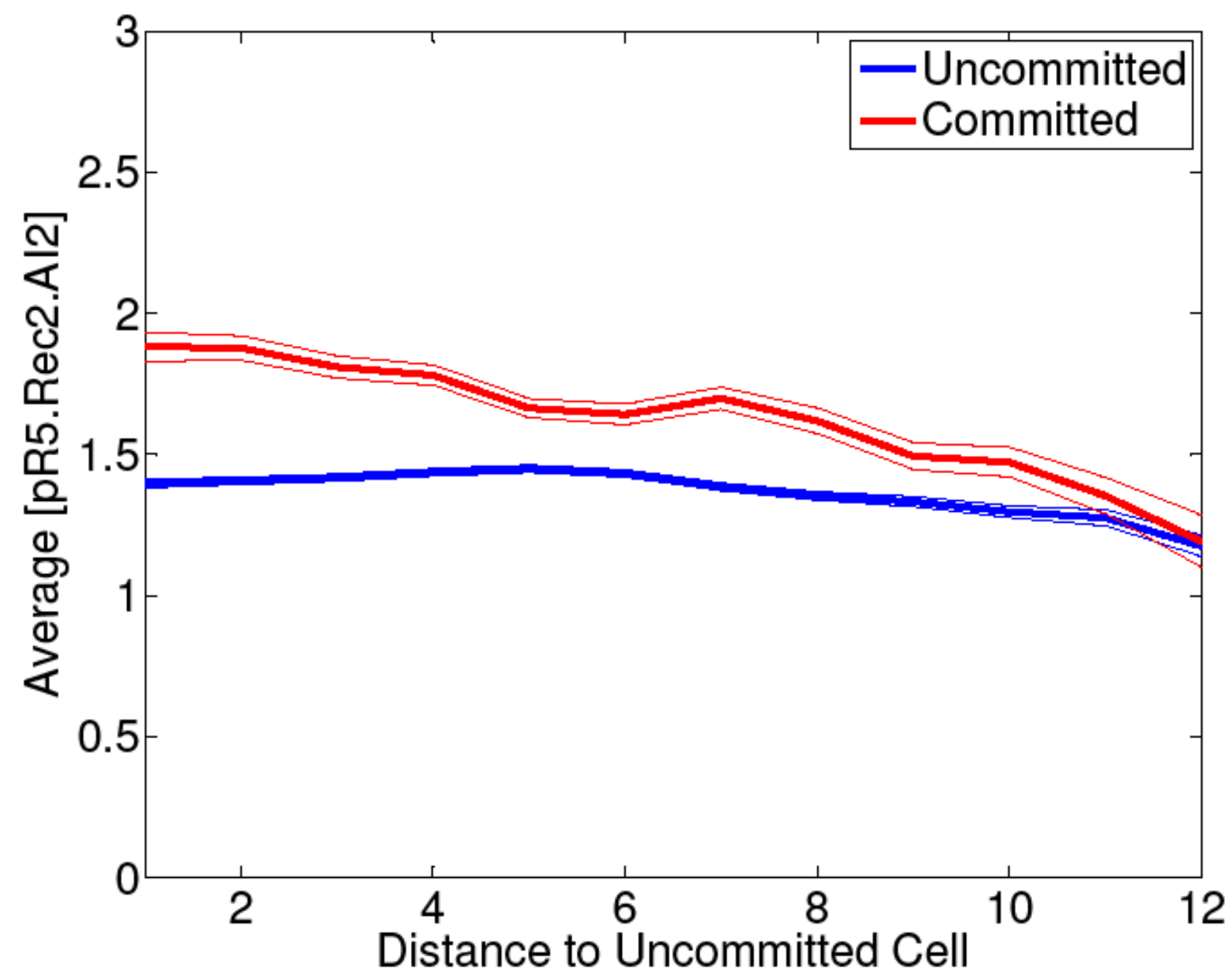**B**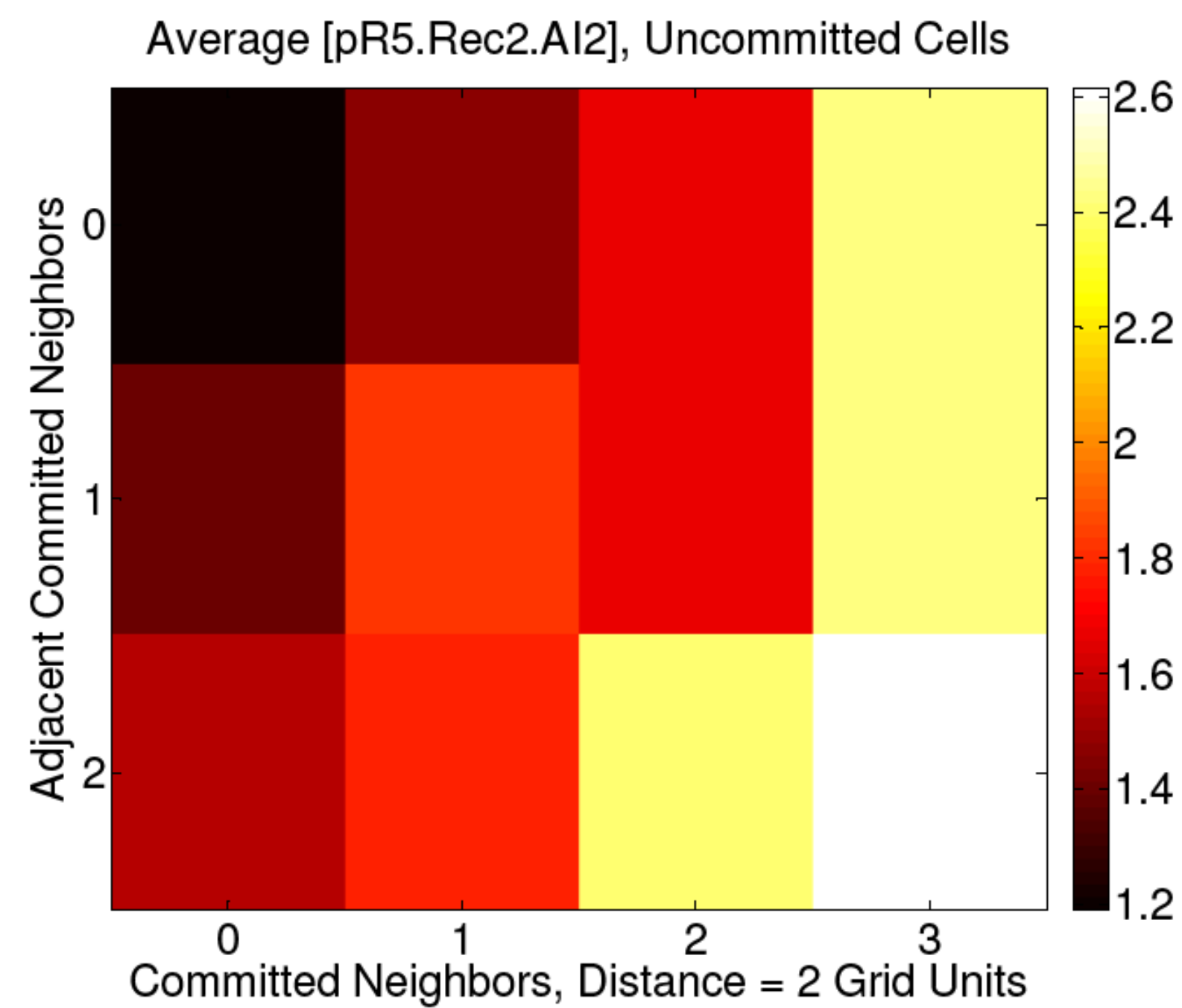**C**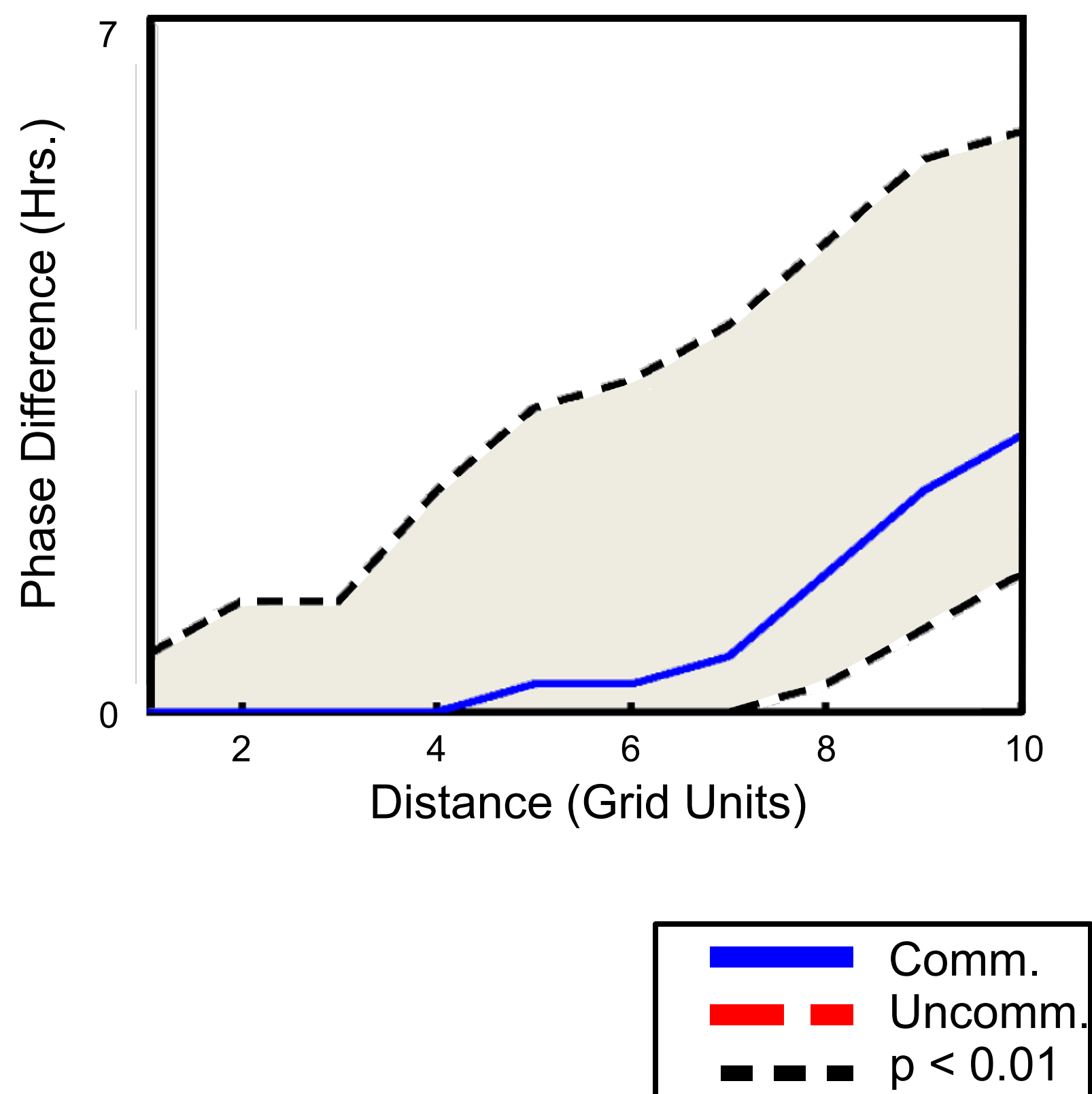**D**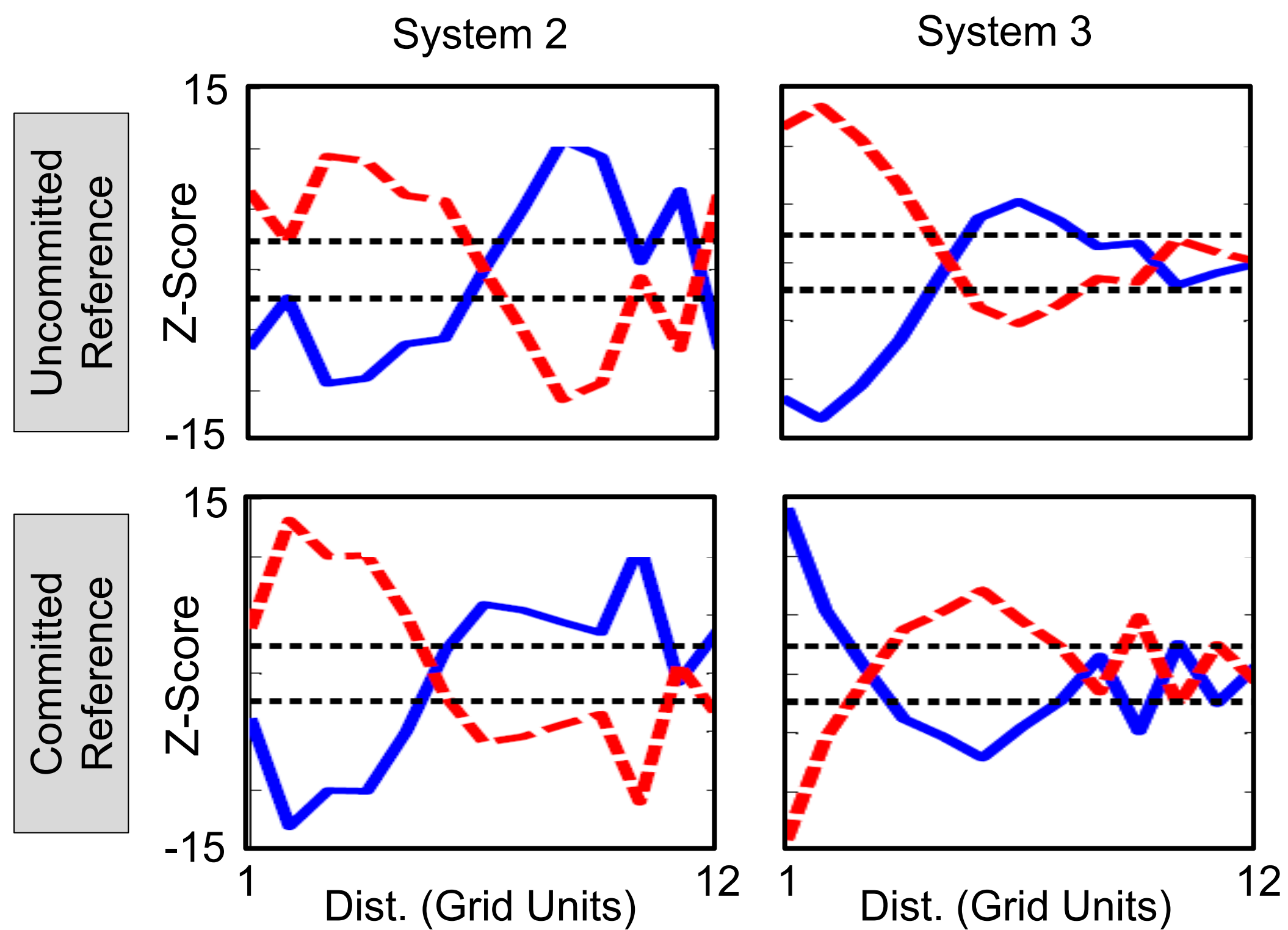

Supplement: Figure S19 — Spatiotemporal analysis of System 3 using the Gillespie model. We define activity for the “Population Control” (PC) module as the level of Rec2.AI2 complex-bound promoter for the R5 repressor (pR5.Rec2.AI2). (A) The thick lines represent the PC activity for uncommitted cells as a function of distance from uncommitted (blue) and committed (red) neighboring cells, averaged over all cells and all time points for a given simulation. Thin lines represent PC activity +/− the standard error of the mean at each distance. (B) Average PC activity for all uncommitted cells over all time points for a given simulation are shown as a function of the number of committed neighbors at one (ordinate) and two (abscissa) grid units away. (C) We measured the time difference between nearest oscillation peaks of dimerized R1 (R1D) for all pairs of coexistent uncommitted cells throughout a given simulation. For example, if four uncommitted cells are alive at a given time point, we would calculate the phase difference among all of the pairs of cells (six in this case). Average phase difference increases as the distance between neighboring cells increases (blue line). The lower and upper black dashed lines represent the first and third quartiles of the phase difference, respectively. Phase difference increases as a function of distance because cells closer together are more likely to have originated from the same parent cell. (D) For a given uncommitted or committed reference cell, the Z-score (see Text S1, Sec. 5.4) indicates the distribution bias of committed and uncommitted neighbors at a given distance (dashed lines, p0.01). Patterning was examined for Systems 2 and 3. (PDF) [file pcbi.1002579.s019.pdf]
