# Supplementary material for: Modular Design of Artificial Tissue Homeostasis: Robust Control through Synthetic Cellular Heterogeneity
Source: PLoS Comput Biol. 2012 Jul 19;8(7):e1002579. doi: 10.1371/journal.pcbi.1002579 (PMC3400602; doi:10.1371/journal.pcbi.1002579)
Supplement: Figure S17 — Population density for different ratios of division and killing rate. Deterministic simulation with a two-population model (A,C) and stochastic simulations of the Systems 2, 3 and 4 (B–D) show qualitatively similar results. (A–B) The population of uncommitted cells remains constant with a small decrease for low rate ratio. (C–D) The population of committed cells follows a power law with an exponent near 1 for low ratio and close to for large ratio. Power laws in (D) are fitted on the results of System 2, the closest to the ODE model. (PDF) [file pcbi.1002579.s017.pdf]

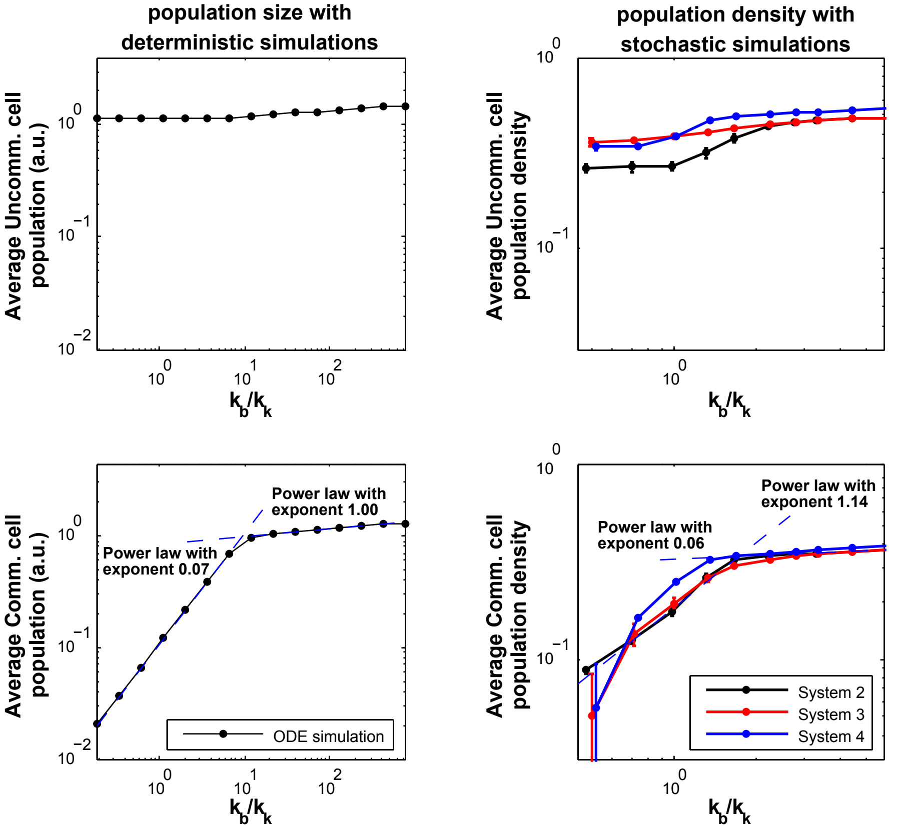
population size with deterministic simulations
population density with stochastic simulations
Average Uncomm. cell population (a.u.)
Average Uncomm. cell population density
Average Comm. cell population (a.u.)
Average Comm. cell population density
k_b/k_k
Power law with exponent 1.00
Power law with exponent 0.07
ODE simulation
Power law with exponent 1.14
Power law with exponent 0.06
System 2
System 3
System 4
