# Supplementary material for: Modular Design of Artificial Tissue Homeostasis: Robust Control through Synthetic Cellular Heterogeneity
Source: PLoS Comput Biol. 2012 Jul 19;8(7):e1002579. doi: 10.1371/journal.pcbi.1002579 (PMC3400602; doi:10.1371/journal.pcbi.1002579)
Supplement: Table S1 — Parameters for the Langevin models of Systems 2 to 4. (PDF) [file pcbi.1002579.s020.pdf]

Module	System 2			System 3			System 4		
	Name	para. #	Value	Name	para. #	Value	Name	para. #	Value
Quorum singaling	k_p^{AI1}	1	2.0	identical			identical		
	H_{AI1}	2	1.5	identical			identical		
	k_d^{AI1}	3	0.2	identical			identical		
	k_p^{AI2}	4	2.0	identical			identical		
	H_{AI2}	5	0.45	identical			identical		
	k_d^{AI2}	6	0.2	identical			identical		
	k_{diff}	7	5.0	identical			identical		
Quorum sensing module	k_p^{A1}	8	1.0	identical to Syst. 2			k_p^{R1}	8	1.0
	H_{A1}	9	2.7	identical to Syst. 2			H_{R1}	9	2.7
	k_d^{A1}	10	1.0	identical to Syst. 2			k_d^{R1}	10	1.0
	k_p^{R2}	11	1.0	identical			identical		
	H_{R2}	12	2.0	identical			identical		
	k_d^{R2}	13	1.0	identical			identical		
	–			k_p^{A3}	14	2.0	identical		
	–			H_{A3-1}	15	0.5	identical		
	–			H_{A3-4}	16	0.5	H_{A3-2}	16	0.5
	–			k_d^{A3}	17	2.0	identical		
Commitment module	k_p^{R5}	18	1.0	identical			identical		
	H_{R5-1}	19	0.6	H_{R5-3}	19	0.6	identical		
	H_{R5-2}	20	0.5	identical to Syst. 2			H_{R5-t}	20	0.9
	k_d^{R5}	21	1.0	identical			identical		
	k_p^{R6}	22	1.0	identical			identical		
	H_{R6-5}	23	0.5	identical			identical		
	H_{R6-7}	24	0.5	identical			identical		
	k_d^{R6}	25	1.0	identical			identical		
	k_p^{R7}	26	3.0	identical			identical		
	H_{R7}	27	0.4	identical			identical		
	k_d^{R7}	28	1.0	identical			identical		
Cell fate parameters	k_p^{GAF}	29	1.7	identical			identical		
	H_{GAF}	30	0.7	identical to Syst. 2			H_{GAF}	30	0.3
	k_d^{GAF}	31	1.0	identical			identical		
	k_p^{GATA}	32	1.0	identical			identical		
	H_{GATA}	33	0.2	identical			identical		
	k_d^{GATA}	34	1.0	identical			identical		
**Additional modules**	–			k_p^{Ao}	35	50	k_p^{At}	35	4.0
	–			k_0^{Ao}	36	0.0002	H_{At}	36	0.5
	–			H_{Ao-A}	37	0.5	k_d^{At}	38	4.0
	–			H_{Ao-R}	38	0.01	k_p^{AI3}	39	200
	–			k_d^{Ao}	39	0.1	H_{AI3-t}	40	0.6
	–			k_p^{Ro}	40	2.5	H_{AI3-7}	37	0.5
	–			H_{Ro}	41	0.5	k_d^{AI3}	41	0.5
	–			k_d^{Ro}	42	0.04	–		
	–			k_p^{Ro2}	43	5	–		
	–			H_{Ro2}	44	0.2	–		
	–			k_d^{Ro2}	45	2.0	–		
	–			k_p^{R4}	46	1.0	–		
	–			H_{R4}	47	0.9	–		
	–			k_d^{R4}	48	1.0	–		

Table S1: Parameters for the Langevin models of Systems 2 to 4
