# Supplementary material for: Modular Design of Artificial Tissue Homeostasis: Robust Control through Synthetic Cellular Heterogeneity
Source: PLoS Comput Biol. 2012 Jul 19;8(7):e1002579. doi: 10.1371/journal.pcbi.1002579 (PMC3400602; doi:10.1371/journal.pcbi.1002579)
Supplement: Table S2 — Scaled parameters for the time-scale analysis. The kinetics parameters ( and ) from Table S1 are scaled by the time-scale parameters according to their module. For each combination of time-scale parameters, the and parameters are used for the Langevin simulations. (PDF) [file pcbi.1002579.s021.pdf]

Quorum signaling module	Quorum sensing module	Toggle switch
$\overline{k_p^{AI1}} = TS_{QS}k_p^{AI1}$<br>$\overline{k_d^{AI1}} = TS_{QS}k_d^{AI1}$	$\overline{k_p^{A1}} = TS_{QM}k_p^{A1}$<br>$\overline{k_d^{A1}} = TS_{QM}k_d^{A1}$	$\overline{k_p^{R5}} = TS_{R5}k_p^{R5}$<br>$\overline{k_d^{R5}} = TS_{R5}k_d^{R5}$
$\overline{k_p^{AI2}} = TS_{QS}k_p^{AI2}$<br>$\overline{k_d^{AI2}} = TS_{QS}k_d^{AI2}$	$\overline{k_p^{R2}} = TS_{QM}k_p^{R2}$<br>$\overline{k_d^{R2}} = TS_{QM}k_d^{R2}$	$\overline{k_p^{R6}} = TS_{R6}k_p^{R6}$<br>$\overline{k_d^{R6}} = TS_{R6}k_d^{R6}$
$\overline{k_{diff}} = TS_{QS}k_{diff}$	(in Systems 3 & 4)<br>$\overline{k_p^{A3}} = TS_{QM}k_p^{A3}$<br>$\overline{k_d^{A3}} = TS_{QM}k_d^{A3}$	$\overline{k_p^{R7}} = TS_{R7}k_p^{R7}$<br>$\overline{k_d^{R7}} = TS_{R7}k_d^{R7}$
(in System 4)<br>$\overline{k_p^{AI3}} = TS_{QS}k_p^{AI3}$<br>$\overline{k_d^{AI3}} = TS_{QS}k_d^{AI3}$		(in System 4)<br>$\overline{k_p^{At}} = TS_{At}k_p^{At}$<br>$\overline{k_d^{At}} = TS_{At}k_d^{At}$

Table S2: Scaled parameters for the time-scale analysis. The kinetics parameters (k_p^{α} and k_d^{α}) from table S1 are scaled by the time-scale parameters TS_{α} according to their module. For each combination of time-scale parameters, the $\overline{k_p^{\alpha}}$ and $\overline{k_d^{\alpha}}$ parameters are used for the Langevin simulations.
