# Supplementary material for: Modular Design of Artificial Tissue Homeostasis: Robust Control through Synthetic Cellular Heterogeneity
Source: PLoS Comput Biol. 2012 Jul 19;8(7):e1002579. doi: 10.1371/journal.pcbi.1002579 (PMC3400602; doi:10.1371/journal.pcbi.1002579)
Supplement: Table S3 — List of reactions for the full multicellular model of System 3. Depending on whether the reactions are associative or dissociative, reaction rates are in units of (molecules per or . (PDF) [file pcbi.1002579.s022.pdf]

**Reactants**	**Products**	**Rate**
pI1	pI1+I1	$5.7 \cdot 10^{-5}$
pI1+R7$_2$	pI1.R7$_2$	$1.0 \cdot 10^{-3}$
pI1.R7$_2$	pI1+R7$_2$	$1.0 \cdot 10^{-3}$
pI1.R7$_2$	pI1	$1.0 \cdot 10^{-3}$
pI1.R7$_2$	pI1.R7$_2$+I1	$1.0 \cdot 10^{-6}$
pI1+Rec1.AI1	pI1.Rec1.AI1	$1.0 \cdot 10^{-1}$
pI1.Rec1.AI1	pI1+Rec1.AI1	$1.6 \cdot 10^{-2}$
pI1.Rec1.AI1	pI1	$2.5 \cdot 10^{-3}$
pI1.Rec1.AI1	pI1.Rec1.AI1+I1	$1.0 \cdot 10^{-1}$
pI1.Rec1.AI1+R7$_2$	pI1.R7$_2$.Rec1.AI1	$1.0 \cdot 10^{-3}$
pI1.R7$_2$+Rec1.AI1	pI1.R7$_2$.Rec1.AI1	$1.0 \cdot 10^{-1}$
pI1.R7$_2$.Rec1.AI1	pI1.Rec1.AI1+R7$_2$	$1.0 \cdot 10^{-3}$
pI1.R7$_2$.Rec1.AI1	pI1.R7$_2$+Rec1.AI1	$1.6 \cdot 10^{-2}$
pI1.R7$_2$.Rec1.AI1	pI1.R7$_2$	$2.5 \cdot 10^{-3}$
pI1.R7$_2$.Rec1.AI1	pI1.Rec1.AI1	$1.0 \cdot 10^{-3}$
pI1.R7$_2$.Rec1.AI1	pI1.R7$_2$.Rec1.AI1+I1	$1.0 \cdot 10^{-6}$
pGAF+Rec1.AI1	pGAF.Rec1.AI1	$8.7 \cdot 10^{-2}$
pGAF.Rec1.AI1	pGAF+Rec1.AI1	$8.0 \cdot 10^{-3}$
pGAF.Rec1.AI1	pGAF	$2.5 \cdot 10^{-3}$
pGAF	pGAF+GAF	$1.0 \cdot 10^{-7}$
pGAF.Rec1.AI1	pGAF.Rec1.AI1+GAF	$2.0 \cdot 10^{-2}$
pRec1	pRec1+Rec1	$1.5 \cdot 10^{-2}$
I1	I1+AI1	$2.1 \cdot 10^{-3}$
AI1		$3.0 \cdot 10^{-3}$
Rec1+AI1	Rec1.AI1	$5.6 \cdot 10^{-5}$
Rec1.AI1	Rec1+AI1	$1.4 \cdot 10^{-3}$
Rec1.AI1		$2.5 \cdot 10^{-3}$
GAF+GAF	GAF$_2$	$5.0 \cdot 10^{-3}$
GAF$_2$	GAF+GAF	$1.0 \cdot 10^{-3}$
GAF		$5.0 \cdot 10^{-4}$
I1		$5.7 \cdot 10^{-3}$
Rec1		$2.5 \cdot 10^{-3}$
GAF$_2$		$1.0 \cdot 10^{-3}$
pAo	pAo+Ao	$1.0 \cdot 10^{-5}$
pAo+Ro$_2$	pAo.Ro$_2$	$1.0 \cdot 10^{-3}$
pAo.Ro$_2$	pAo+Ro$_2$	$1.0 \cdot 10^{-7}$
pAo.Ro$_2$	pAo	$1.0 \cdot 10^{-5}$
pAo.Ro$_2$	pAo.Ro$_2$+Ao	$1.0 \cdot 10^{-7}$
pAo+Ao$_2$	pAo.Ao$_2$	$1.0 \cdot 10^{-3}$
pAo.Ao$_2$	pAo+Ao$_2$	$5.0 \cdot 10^{-4}$
pAo.Ao$_2$	pAo	$1.0 \cdot 10^{-4}$
pAo.Ao$_2$	pAo.Ao$_2$+Ao	$4.0 \cdot 10^{-2}$
pAo.Ao$_2$+Ro$_2$	pAo.Ro$_2$.Ao$_2$	$1.0 \cdot 10^{-3}$
pAo.Ro$_2$+Ao$_2$	pAo.Ro$_2$.Ao$_2$	$1.0 \cdot 10^{-3}$
pAo.Ro$_2$.Ao$_2$	pAo.Ao$_2$+Ro$_2$	$1.0 \cdot 10^{-7}$
pAo.Ro$_2$.Ao$_2$	pAo.Ro$_2$+Ao$_2$	$5.0 \cdot 10^{-4}$
pAo.Ro$_2$.Ao$_2$	pAo.Ro$_2$	$1.0 \cdot 10^{-4}$
pAo.Ro$_2$.Ao$_2$	pAo.Ao$_2$	$1.0 \cdot 10^{-5}$
pAo.Ro$_2$.Ao$_2$	pAo.Ro$_2$.Ao$_2$+Ao	$1.0 \cdot 10^{-7}$

Table S3: List of reactions for the full multicellular model of System 3. Depending on whether the reactions are associative or dissociative, reaction rates are in units of (molecules per cell)$^{-1}$(s)$^{-1}$ or s^{-1}.

Reactants	Products	Rate
pRo	pRo+Ro+Ro2	$1.0 \cdot 10^{-5}$
pRo+Ao_2	pRo.Ao_2	$1.0 \cdot 10^{-3}$
pRo.Ao_2	pRo+Ao_2	$5.0 \cdot 10^{-4}$
pRo.Ao_2	pRo	$1.0 \cdot 10^{-4}$
pRo.Ao_2	pRo.Ao_2+Ro+Ro2	$5.0 \cdot 10^{-3}$
pRo.Ao_2+Ao_2	pRo.Ao_2.Ao_2	$1.0 \cdot 10^{-3}$
pRo.Ao_2.Ao_2	pRo.Ao_2+Ao_2	$5.0 \cdot 10^{-4}$
pRo.Ao_2.Ao_2	pRo.Ao_2	$1.0 \cdot 10^{-4}$
pRo.Ao_2.Ao_2	pRo.Ao_2.Ao_2+Ro+Ro2	$5.0 \cdot 10^{-3}$
Ro		$4.0 \cdot 10^{-5}$
Ro2		$1.0 \cdot 10^{-3}$
Ao		$1.0 \cdot 10^{-4}$
pR4	pR4+R4	$1.0 \cdot 10^{-2}$
pR4+$Ro2_2$	pR4.$Ro2_2$	$7.0 \cdot 10^{-4}$
pR4.$Ro2_2$	pR4+$Ro2_2$	$1.0 \cdot 10^{-3}$
pR4.$Ro2_2$	pR4	$1.0 \cdot 10^{-3}$
pR4.$Ro2_2$	pR4.$Ro2_2$+R4	$1.0 \cdot 10^{-3}$
pR4.$Ro2_2$+$Ro2_2$	pR4.$Ro2_2$.$Ro2_2$	$7.0 \cdot 10^{-4}$
pR4.$Ro2_2$.$Ro2_2$	pR4.$Ro2_2$+$Ro2_2$	$1.0 \cdot 10^{-3}$
pR4.$Ro2_2$.$Ro2_2$	pR4.$Ro2_2$	$1.0 \cdot 10^{-3}$
pR4.$Ro2_2$.$Ro2_2$	pR4.$Ro2_2$.$Ro2_2$+R4	$1.0 \cdot 10^{-3}$
R4		$1.0 \cdot 10^{-3}$
pA3	pA3+A3	$1.0 \cdot 10^{-6}$
pA3+$R4_2$	pA3.$R4_2$	$2.0 \cdot 10^{-4}$
pA3.$R4_2$	pA3+$R4_2$	$1.0 \cdot 10^{-3}$
pA3.$R4_2$	pA3	$1.0 \cdot 10^{-3}$
pA3.$R4_2$	pA3.$R4_2$+A3	$1.0 \cdot 10^{-6}$
pA3.$R4_2$+$R4_2$	pA3.$R4_2$.$R4_2$	$2.0 \cdot 10^{-4}$
pA3.$R4_2$.$R4_2$	pA3.$R4_2$+$R4_2$	$1.0 \cdot 10^{-3}$
pA3.$R4_2$.$R4_2$	pA3.$R4_2$	$1.0 \cdot 10^{-3}$
pA3.$R4_2$.$R4_2$	pA3.$R4_2$.$R4_2$+A3	$1.0 \cdot 10^{-6}$
pA3+Rec1.AI1	pA3.Rec1.AI1	$8.7 \cdot 10^{-2}$
pA3.Rec1.AI1	pA3+Rec1.AI1	$8.0 \cdot 10^{-3}$
pA3.Rec1.AI1	pA3	$2.5 \cdot 10^{-3}$
pA3.Rec1.AI1	pA3.Rec1.AI1+A3	$2.0 \cdot 10^{-2}$
pA3.Rec1.AI1+$R4_2$	pA3.$R4_2$.Rec1.AI1	$2.0 \cdot 10^{-4}$
pA3.$R4_2$+Rec1.AI1	pA3.$R4_2$.Rec1.AI1	$8.7 \cdot 10^{-2}$
pA3.$R4_2$.Rec1.AI1	pA3.Rec1.AI1+$R4_2$	$1.0 \cdot 10^{-3}$
pA3.$R4_2$.Rec1.AI1	pA3.$R4_2$+Rec1.AI1	$8.0 \cdot 10^{-3}$
pA3.$R4_2$.Rec1.AI1	pA3.$R4_2$	$2.5 \cdot 10^{-3}$
pA3.$R4_2$.Rec1.AI1	pA3.Rec1.AI1	$1.0 \cdot 10^{-3}$
pA3.$R4_2$.Rec1.AI1	pA3.$R4_2$.Rec1.AI1+A3	$1.0 \cdot 10^{-6}$
pA3.$R4_2$.Rec1.AI1+$R4_2$	pA3.$R4_2$.$R4_2$.Rec1.AI1	$2.0 \cdot 10^{-4}$
pA3.$R4_2$.$R4_2$+Rec1.AI1	pA3.$R4_2$.$R4_2$.Rec1.AI1	$8.7 \cdot 10^{-2}$
pA3.$R4_2$.$R4_2$.Rec1.AI1	pA3.$R4_2$.Rec1.AI1+$R4_2$	$1.0 \cdot 10^{-3}$
pA3.$R4_2$.$R4_2$.Rec1.AI1	pA3.$R4_2$.$R4_2$+Rec1.AI1	$8.0 \cdot 10^{-3}$
pA3.$R4_2$.$R4_2$.RhlR.AI1	pA3.$R4_2$.$R4_2$	$2.5 \cdot 10^{-3}$
pA3.$R4_2$.$R4_2$.Rec1.AI1	pA3.$R4_2$.Rec1.AI1	$1.0 \cdot 10^{-3}$
pA3.$R4_2$.$R4_2$.Rec1.AI1	pA3.$R4_2$.$R4_2$.Rec1.AI1+A3	$1.0 \cdot 10^{-6}$
A3		$1.0 \cdot 10^{-3}$

Table S3 continued.

Reactants	Products	Rate
pR5	pR5+R5	$1.0 \cdot 10^{-6}$
pR5+A3$_2$	pR5.A3$_2$	$1.0 \cdot 10^{-4}$
pR5.A3$_2$	pR5+A3$_2$	$1.0 \cdot 10^{-3}$
pR5.A3$_2$	pR5	$5.0 \cdot 10^{-4}$
pR5.A3$_2$	pR5.A3$_2$+R5	$1.0 \cdot 10^{-5}$
pR5.A3$_2$+A3$_2$	pR5.A3$_2$.A3$_2$	$1.0 \cdot 10^{-4}$
pR5.A3$_2$.A3$_2$	pR5.A3$_2$+A3$_2$	$1.0 \cdot 10^{-3}$
pR5.A3$_2$.A3$_2$	pR5.A3$_2$	$5.0 \cdot 10^{-4}$
pR5.A3$_2$.A3$_2$	pR5.A3$_2$.A3$_2$+R5	$1.0 \cdot 10^{-2}$
pR5+R2$_2$	pR5.R2$_2$	$3.0 \cdot 10^{-4}$
pR5.R2$_2$	pR5+R2$_2$	$1.0 \cdot 10^{-3}$
pR5.R2$_2$	pR5	$1.0 \cdot 10^{-3}$
pR5.R2$_2$	pR5.R2$_2$+R5	$1.0 \cdot 10^{-6}$
pR5.R2$_2$+A3$_2$	pR5.A3$_2$.R2$_2$	$1.0 \cdot 10^{-4}$
pR5.A3$_2$+R2$_2$	pR5.A3$_2$.R2$_2$	$3.0 \cdot 10^{-4}$
pR5.A3$_2$.R2$_2$	pR5.R2$_2$+A3$_2$	$1.0 \cdot 10^{-3}$
pR5.A3$_2$.R2$_2$	pR5.A3$_2$+R2$_2$	$1.0 \cdot 10^{-3}$
pR5.A3$_2$.R2$_2$	pR5.A3$_2$	$1.0 \cdot 10^{-3}$
pR5.A3$_2$.R2$_2$	pR5.R2$_2$	$5.0 \cdot 10^{-4}$
pR5.A3$_2$.R2$_2$	pR5.A3$_2$.R2$_2$+R5	$1.0 \cdot 10^{-6}$
pR5.A3$_2$.R2$_2$+A3$_2$	pR5.A3$_2$.A3$_2$.R2$_2$	$1.0 \cdot 10^{-4}$
pR5.A3$_2$.A3$_2$+R2$_2$	pR5.A3$_2$.A3$_2$.R2$_2$	$3.0 \cdot 10^{-4}$
pR5.A3$_2$.A3$_2$.R2$_2$	pR5.A3$_2$.R2$_2$+A3$_2$	$1.0 \cdot 10^{-3}$
pR5.A3$_2$.A3$_2$.R2$_2$	pR5.A3$_2$.A3$_2$+R2$_2$	$1.0 \cdot 10^{-3}$
pR5.A3$_2$.A3$_2$.R2$_2$	pR5.A3$_2$.A3$_2$	$1.0 \cdot 10^{-3}$
pR5.A3$_2$.A3$_2$.R2$_2$	pR5.A3$_2$.R2$_2$	$5.0 \cdot 10^{-4}$
pR5.A3$_2$.A3$_2$.R2$_2$	pR5.A3$_2$.A3$_2$.R2$_2$+R5	$1.0 \cdot 10^{-6}$
R5		$1.0 \cdot 10^{-3}$
pR2	pR2+R2	$1.0 \cdot 10^{-5}$
pR2+Rec2.AI2	pR2.Rec2.AI2	$3.0 \cdot 10^{-4}$
pR2.Rec2.AI2	pR2+Rec2.AI2	$1.0 \cdot 10^{-3}$
pR2.Rec2.AI2	pR2	$1.0 \cdot 10^{-3}$
pR2.Rec2.AI2	pR2.Rec2.AI2+R2	$1.0 \cdot 10^{-2}$
R2		$1.0 \cdot 10^{-3}$
R2+R2	R2$_2$	$5.0 \cdot 10^{-4}$
R2$_2$	R2+R2	$1.0 \cdot 10^{-3}$
R2$_2$		$1.0 \cdot 10^{-3}$
pI2	pI2+I2	$2.0 \cdot 10^{-3}$
pI2+R6$_2$	pI2.R6$_2$	$1.0 \cdot 10^{-3}$
pI2.R6$_2$	pI2+R6$_2$	$1.0 \cdot 10^{-3}$
pI2.R6$_2$	pI2	$1.0 \cdot 10^{-3}$
pI2.R6$_2$	pI2.R6$_2$+I2	$1.0 \cdot 10^{-5}$
pI2+Rec2.AI2	pI2.Rec2.AI2	$3.0 \cdot 10^{-4}$
pI2.Rec2.AI2	pI2+Rec2.AI2	$1.0 \cdot 10^{-3}$
pI2.Rec2.AI2	pI2	$1.0 \cdot 10^{-3}$
pI2.Rec2.AI2	pI2.Rec2.AI2+I2	$1.0 \cdot 10^{-2}$
pI2.Rec2.AI2+R6$_2$	pI2.R6$_2$.Rec2.AI2	$1.0 \cdot 10^{-3}$
pI2.R6$_2$+Rec2.AI2	pI2.R6$_2$.Rec2.AI2	$3.0 \cdot 10^{-4}$
pI2.R6$_2$.Rec2.AI2	pI2.Rec2.AI2+R6$_2$	$1.0 \cdot 10^{-3}$
pI2.R6$_2$.Rec2.AI2	pI2.R6$_2$+Rec2.AI2	$1.0 \cdot 10^{-3}$
pI2.R6$_2$.Rec2.AI2	pI2.R6$_2$	$1.0 \cdot 10^{-3}$
pI2.R6$_2$.Rec2.AI2	pI2.Rec2.AI2	$1.0 \cdot 10^{-3}$
pI2.R6$_2$.Rec2.AI2	pI2.R6$_2$.Rec2.AI2+I2	$1.0 \cdot 10^{-5}$

Table S3 continued.

Reactants	Products	Rate
I2	I2+AI2	$1.2 \cdot 10^{-3}$
AI2		$1.0 \cdot 10^{-3}$
Rec2+AI2	Rec2.AI2	$3.0 \cdot 10^{-4}$
Rec2.AI2	Rec2+AI2	$1.0 \cdot 10^{-3}$
Rec2.AI2		$1.0 \cdot 10^{-3}$
pRec2	pRec2+Rec2	$4.4 \cdot 10^{-4}$
pRec2+Rec2.AI2	pRec2.Rec2.AI2	$3.3 \cdot 10^{-4}$
pRec2.Rec2.AI2	pRec2+Rec2.AI2	$1.0 \cdot 10^{-3}$
pRec2.Rec2.AI2	pRec2	$1.0 \cdot 10^{-3}$
pRec2.Rec2.AI2	pRec2.Rec2.AI2+Rec2	$5.3 \cdot 10^{-3}$
I2		$1.0 \cdot 10^{-3}$
Rec2		$1.7 \cdot 10^{-3}$
pR6	pR6+R6	$2.0 \cdot 10^{-2}$
pR6+$R5_2$	pR6.$R5_2$	$1.0 \cdot 10^{-3}$
pR6.$R5_2$	pR6+$R5_2$	$1.0 \cdot 10^{-3}$
pR6.$R5_2$	pR6	$1.0 \cdot 10^{-3}$
pR6.$R5_2$	pR6.$R5_2$+R6	$1.0 \cdot 10^{-6}$
pR6.$R5_2$+$R5_2$	pR6.$R5_2$.$R5_2$	$1.0 \cdot 10^{-3}$
pR6.$R5_2$.$R5_2$	pR6.$R5_2$+$R5_2$	$1.0 \cdot 10^{-3}$
pR6.$R5_2$.$R5_2$	pR6.$R5_2$	$1.0 \cdot 10^{-3}$
pR6.$R5_2$.$R5_2$	pR6.$R5_2$.$R5_2$+R6	$1.0 \cdot 10^{-6}$
pR6+$R7_2$	pR6.$R7_2$	$8.0 \cdot 10^{-4}$
pR6.$R7_2$	pR6+$R7_2$	$1.0 \cdot 10^{-3}$
pR6.$R7_2$	pR6	$1.0 \cdot 10^{-3}$
pR6.$R7_2$	pR6.$R7_2$+R6	$1.0 \cdot 10^{-6}$
pR6.$R7_2$+$R5_2$	pR6.$R5_2$.$R7_2$	$1.0 \cdot 10^{-3}$
pR6.$R5_2$+$R7_2$	pR6.$R5_2$.$R7_2$	$8.0 \cdot 10^{-4}$
pR6.$R5_2$.$R7_2$	pR6.$R7_2$+$R5_2$	$1.0 \cdot 10^{-3}$
pR6.$R5_2$.$R7_2$	pR6.$R5_2$+$R7_2$	$1.0 \cdot 10^{-3}$
pR6.$R5_2$.$R7_2$	pR6.$R5_2$	$1.0 \cdot 10^{-3}$
pR6.$R5_2$.$R7_2$	pR6.$R7_2$	$1.0 \cdot 10^{-3}$
pR6.$R5_2$.$R7_2$	pR6.$R5_2$.$R7_2$+R6	$1.0 \cdot 10^{-6}$
pR6.$R5_2$.$R7_2$+$R5_2$	pR6.$R5_2$.$R5_2$.$R7_2$	$1.0 \cdot 10^{-3}$
pR6.$R5_2$.$R5_2$+$R7_2$	pR6.$R5_2$.$R5_2$.$R7_2$	$8.0 \cdot 10^{-4}$
pR6.$R5_2$.$R5_2$.$R7_2$	pR6.$R5_2$.$R7_2$+$R5_2$	$1.0 \cdot 10^{-3}$
pR6.$R5_2$.$R5_2$.$R7_2$	pR6.$R5_2$.$R5_2$+$R7_2$	$1.0 \cdot 10^{-3}$
pR6.$R5_2$.$R5_2$.$R7_2$	pR6.$R5_2$.$R5_2$	$1.0 \cdot 10^{-3}$
pR6.$R5_2$.$R5_2$.$R7_2$	pR6.$R5_2$.$R7_2$	$1.0 \cdot 10^{-3}$
pR6.$R5_2$.$R5_2$.$R7_2$	pR6.$R5_2$.$R5_2$.$R7_2$+R6	$1.0 \cdot 10^{-6}$
pR6.$R7_2$+$R7_2$	pR6.$R7_2$.$R7_2$	$8.0 \cdot 10^{-4}$
pR6.$R7_2$.$R7_2$	pR6.$R7_2$+$R7_2$	$1.0 \cdot 10^{-3}$
pR6.$R7_2$.$R7_2$	pR6.$R7_2$	$1.0 \cdot 10^{-3}$
pR6.$R7_2$.$R7_2$	pR6.$R7_2$.$R7_2$+R6	$1.0 \cdot 10^{-6}$
pR6.$R7_2$.$R7_2$+$R5_2$	pR6.$R5_2$.$R7_2$.$R7_2$	$1.0 \cdot 10^{-3}$
pR6.$R5_2$.$R7_2$+$R7_2$	pR6.$R5_2$.$R7_2$.$R7_2$	$8.0 \cdot 10^{-4}$
pR6.$R5_2$.$R7_2$.$R7_2$	pR6.$R7_2$.$R7_2$+$R5_2$	$1.0 \cdot 10^{-3}$
pR6.$R5_2$.$R7_2$.$R7_2$	pR6.$R5_2$.$R7_2$+$R7_2$	$1.0 \cdot 10^{-3}$
pR6.$R5_2$.$R7_2$.$R7_2$	pR6.$R5_2$.$R7_2$	$1.0 \cdot 10^{-3}$
pR6.$R5_2$.$R7_2$.$R7_2$	pR6.$R7_2$.$R7_2$	$1.0 \cdot 10^{-3}$
pR6.$R5_2$.$R7_2$.$R7_2$	pR6.$R5_2$.$R7_2$.$R7_2$+R6	$1.0 \cdot 10^{-6}$
pR6.$R5_2$.$R7_2$.$R7_2$+$R5_2$	pR6.$R5_2$.$R5_2$.$R7_2$.$R7_2$	$1.0 \cdot 10^{-3}$

Table S3 continued.

**Reactants**	**Products**	**Rate**
$pR6.R5_2.R5_2.R7_2$+$R7_2$	$pR6.R5_2.R5_2.R7_2.R7_2$	$8.0 \cdot 10^{-4}$
$pR6.R5_2.R5_2.R7_2.R7_2$	$pR6.R5_2.R7_2.R7_2$+$R5_2$	$1.0 \cdot 10^{-3}$
$pR6.R5_2.R5_2.R7_2.R7_2$	$pR6.R5_2.R5_2.R7_2$+$R7_2$	$1.0 \cdot 10^{-3}$
$pR6.R5_2.R5_2.R7_2.R7_2$	$pR6.R5_2.R5_2.R7_2$	$1.0 \cdot 10^{-3}$
$pR6.R5_2.R5_2.R7_2.R7_2$	$pR6.R5_2.R7_2.R7_2$	$1.0 \cdot 10^{-3}$
$pR6.R5_2.R5_2.R7_2.R7_2$	$pR6.R5_2.R5_2.R7_2.R7_2$+R6	$1.0 \cdot 10^{-6}$
$pR6.R5_2.R7_2$+$R5_2$	$pR6.R5_2.R5_2.R7_2$	$1.0 \cdot 10^{-3}$
$pR6.R5_2.R5_2$+$R7_2$	$pR6.R5_2.R5_2.R7_2$	$8.0 \cdot 10^{-4}$
$pR6.R5_2.R5_2.R7_2$	$pR6.R5_2.R7_2$+$R5_2$	$1.0 \cdot 10^{-3}$
$pR6.R5_2.R5_2.R7_2$	$pR6.R5_2.R5_2$+$R7_2$	$1.0 \cdot 10^{-3}$
$pR6.R5_2.R5_2.R7_2$	$pR6.R5_2.R5_2$	$1.0 \cdot 10^{-3}$
$pR6.R5_2.R5_2.R7_2$	$pR6.R5_2.R7_2$	$1.0 \cdot 10^{-3}$
$pR6.R5_2.R5_2.R7_2$	$pR6.R5_2.R5_2.R7_2$+R6	$1.0 \cdot 10^{-6}$
R6		$4.0 \cdot 10^{-3}$
R6+R6	$R6_2$	$1.0 \cdot 10^{-3}$
$R6_2$	R6+R6	$1.0 \cdot 10^{-3}$
$R6_2$		$2.0 \cdot 10^{-3}$
pR7	pR7+R7	$2.0 \cdot 10^{-2}$
pR7+$R6_2$	$pR7.R6_2$	$1.0 \cdot 10^{-3}$
$pR7.R6_2$	pR7+$R6_2$	$1.0 \cdot 10^{-3}$
$pR7.R6_2$	pR7	$1.0 \cdot 10^{-3}$
$pR7.R6_2$	$pR7.R6_2$+R7	$1.0 \cdot 10^{-6}$
$pR7.R6_2$+$R6_2$	$pR7.R6_2.R6_2$	$1.0 \cdot 10^{-3}$
$pR7.R6_2.R6_2$	$pR7.R6_2$+$R6_2$	$1.0 \cdot 10^{-3}$
$pR7.R6_2.R6_2$	$pR7.R6_2$	$1.0 \cdot 10^{-3}$
$pR7.R6_2.R6_2$	$pR7.R6_2.R6_2$+R7	$1.0 \cdot 10^{-6}$
R7		$4.0 \cdot 10^{-3}$
R7+R7	$R7_2$	$1.0 \cdot 10^{-3}$
$R7_2$	R7+R7	$1.0 \cdot 10^{-3}$
$R7_2$		$2.0 \cdot 10^{-3}$
pGATA4	pGATA4+GATA4	$5.0 \cdot 10^{-3}$
$pGATA4.R6_2$	$pGATA4.R6_2$+GATA4	$1.0 \cdot 10^{-6}$
pGATA4+$R6_2$	$pGATA4.R6_2$	$1.0 \cdot 10^{-3}$
$pGATA4.R6_2$	pGATA4+$R6_2$	$1.0 \cdot 10^{-3}$
GATA4		$1.0 \cdot 10^{-3}$
$pGATA4.R6_2$	pGATA4	$1.0 \cdot 10^{-3}$
pNotEndo	pNotEndo+NotEndo	$1.0 \cdot 10^{-2}$
pNotEndo+GATA4	pNotEndo.GATA4	$7.0 \cdot 10^{-4}$
pNotEndo.GATA4	pNotEndo+GATA4	$1.0 \cdot 10^{-3}$
pNotEndo.GATA4	pNotEndo	$1.0 \cdot 10^{-3}$
pNotEndo+Endo	pNotEndo.Endo	$1.0 \cdot 10^{-3}$
pNotEndo.Endo	pNotEndo+Endo	$1.0 \cdot 10^{-3}$
pNotEndo.Endo	pNotEndo	$1.0 \cdot 10^{-3}$
NotEndo		$1.0 \cdot 10^{-3}$

Table S3 continued.

**Reactants**	**Products**	**Rate**
pEndo	pEndo+Endo+GAF2	$5.0 \cdot 10^{-3}$
pEndo.NotEndo	pEndo.NotEndo+Endo+GAF2	$1.0 \cdot 10^{-7}$
pEndo	pEndo+E	$3.0 \cdot 10^{-5}$
pEndo+NotEndo	pEndo.NotEndo	$1.0 \cdot 10^{-3}$
pEndo.NotEndo	pEndo+NotEndo	$1.0 \cdot 10^{-3}$
pEndo.NotEndo	pEndo	$1.0 \cdot 10^{-3}$
Endo		$1.0 \cdot 10^{-3}$
Ao+Ao	Ao_2	$1.0 \cdot 10^{-3}$
Ao_2	Ao+Ao	$5.0 \cdot 10^{-3}$
Ao_2		$1.0 \cdot 10^{-4}$
Ro+Ro	Ro_2	$1.0 \cdot 10^{-3}$
Ro_2	Ro+Ro	$2.5 \cdot 10^{-2}$
Ro_2		$2.0 \cdot 10^{-5}$
Ro2+Ro2	$Ro2_2$	$1.0 \cdot 10^{-3}$
$Ro2_2$	Ro2+Ro2	$1.0 \cdot 10^{-3}$
$Ro2_2$		$1.0 \cdot 10^{-3}$
R4+R4	$R4_2$	$1.0 \cdot 10^{-3}$
$R4_2$	R4+R4	$1.0 \cdot 10^{-3}$
$R4_2$		$1.0 \cdot 10^{-3}$
R5+R5	$R5_2$	$1.0 \cdot 10^{-3}$
$R5_2$	R5+R5	$1.0 \cdot 10^{-3}$
$R5_2$		$1.0 \cdot 10^{-3}$
A3+A3	$A3_2$	$5.0 \cdot 10^{-3}$
$A3_2$	A3+A3	$1.0 \cdot 10^{-3}$
$A3_2$		$5.0 \cdot 10^{-4}$
CELL AI1	GRID AI1	$1.0 \cdot 10^{-1}$
GRID AI1	CELL AI1	$1.0 \cdot 10^{-1}$
CELL AI2	GRID AI2	$1.0 \cdot 10^{-1}$
GRID AI2	CELL AI2	$1.0 \cdot 10^{-1}$
GRID AI1		$3.0 \cdot 10^{-3}$
GRID AI2		$1.0 \cdot 10^{-3}$

Table S3 continued.
