# Supplementary material for: Modular Design of Artificial Tissue Homeostasis: Robust Control through Synthetic Cellular Heterogeneity
Source: PLoS Comput Biol. 2012 Jul 19;8(7):e1002579. doi: 10.1371/journal.pcbi.1002579 (PMC3400602; doi:10.1371/journal.pcbi.1002579)
Supplement: Table S4 — Phenotypes for the oscillator module (see Figure 8A ). (PDF) [file pcbi.1002579.s023.pdf]

Phenotype Name	Description
Duration High	Average duration of the intervals where $R4$ concentration is above 50% of the dynamic range.
Duration Low	Average duration of the intervals where $R4$ concentration is below 50% of the dynamic range.
Period	Average time interval between two switches from high to low concentrations of $R4$.
Fraction High	Fraction of the time when $R4$ concentration is high (*Duration High*/*Period*).
$\sigma_{\text{Duration High}}$	Standard deviation of the duration of the intervals where $R4$ concentration is high.
$\sigma_{\text{Duration Low}}$	Standard deviation of the duration of the intervals where $R4$ concentration is high.
σ_{Period}	Standard deviation of the time interval between two switches from high to low concentrations of $R4$.
High Value	Average concentration of $R4$ when $R4$ has a high concentration.
Low Value	Average concentration of $R4$ when $R4$ has a low concentration.
Dynamic Range	Difference between the high and low values divided by the high value.
$\text{CV}_{\text{Period}}$	Coefficient of variation of the Period: standard deviation of the Period divided by its mean ($\sigma_{\text{Period}}/Period$).
$\text{CV}^{\text{P}}_{\text{High}}$	Standard deviation of the Duration High divided by the mean Period ($\sigma_{\text{Duration High}}/Period$).
$\text{CV}^{\text{P}}_{\text{Low}}$	Standard deviation of the Duration Low divided by the mean Period ($\sigma_{\text{Duration Low}}/Period$).
CV_{High}	Standard deviation of the duration of the intervals where $R4$ concentration is high divided by its mean ($\sigma_{\text{Duration High}}/Duration\ High$).
CV_{Low}	Standard deviation of the duration of the intervals where $R4$ concentration is low divided by its mean ($\sigma_{\text{Duration Low}}/Duration\ Low$).
Peak Integral	*Duration High* multiplied by *High Value*.

Table S4: Phenotypes for the oscillator module (see Figure 8A).
