# Supplementary material for: Modular Design of Artificial Tissue Homeostasis: Robust Control through Synthetic Cellular Heterogeneity
Source: PLoS Comput Biol. 2012 Jul 19;8(7):e1002579. doi: 10.1371/journal.pcbi.1002579 (PMC3400602; doi:10.1371/journal.pcbi.1002579)
Supplement: Table S5 — Phenotypes for the throttle module (see Figure 8F ). (PDF) [file pcbi.1002579.s024.pdf]

Phenotype Name	Description
Switch	Fraction of toggles that switch after the input (a switch occurs when $R7$ reaches a steady state with a concentration above 2 a.u.).
$R7$ Value	Average concentration of $R7$ at steady state (concentration above 2 a.u.) after a switch.
$R7$ T to St. St.	Average time for $R7$ to reach steady state (defined as the time when $R7$ concentration reaches $R7\ Value - 2 \cdot R7\ Noise$) after input (when a switch occurs).
σ $R7$ T to St. St.	Standard deviation of the time for $R7$ to reach steady state after input (when a switch occurs).
$AI3$ Value	Average concentration of $AI3$ at steady state (defines as 1 hour after $R7$ reaches steady state) after a switch.
$AI3$ Noise	Standard deviation of $AI3$ concentration at steady state after a switch.
Peak	Average peak (maximal) value of $AI3$ concentration after input (when a switch occurs).
T to Peak	Average time for $AI3$ to reach its maximal concentration after input (when a switch occurs).
σ T to Peak	Standard deviation of the time for $AI3$ to reach its maximal concentration after input (when a switch occurs).
T to St. St.	Average time for $AI3$ to reach steady state (defined as the time when $AI3$ concentration decreases below $AI3\ Value + 2 \cdot AI3\ Noise$) after peak (when a switch occurs).
σ T to St. St.	Standard deviation of the time for $AI3$ to reach steady state after peak (when a switch occurs).
Value before Input	Average concentration of $AI3$ at steady state before input.
Noise before Input	Standard deviation of $AI3$ concentration at steady state before input.
No Switch Value	Average concentration of $AI3$ at steady state after input when no switch occurs ($R7$ remains below 2 a.u. after input).
No Switch Noise	Standard deviation of the concentration of $AI3$ at steady state after input when no switch occurs.
No Switch Peak	Average maximal concentration of $AI3$ at steady state after input when no switch occurs.

Table S5: Phenotypes for the throttle module (see also Figure 8F).
