# Supplementary material for: Modular Design of Artificial Tissue Homeostasis: Robust Control through Synthetic Cellular Heterogeneity
Source: PLoS Comput Biol. 2012 Jul 19;8(7):e1002579. doi: 10.1371/journal.pcbi.1002579 (PMC3400602; doi:10.1371/journal.pcbi.1002579)
Supplement: Table S6 — Features used to analyze throttle behavior. These features were measured for each throttle phenotype (see Table S5), where “image” refers to the observed phenotype as a response to the two inputs, and (see Figure S14). (PDF) [file pcbi.1002579.s025.pdf]

Feature Name	Description
Value$_{x=[AI3],y=[A3]}$	Phenotype value at a particular pair of [AI3],[A3] input values.
Integral	Sum of all values over the image.
Max	Max value over the image.
Min	Min value over the image.
Mean	Mean value over the image.
σ	Standard deviation over the image.
Entropy	Entropy over the image.
Contrast	See `graycoprops()`, Matlab (Natick, MA)
Correlation	See `graycoprops()`, Matlab (Natick, MA)
Energy	See `graycoprops()`, Matlab (Natick, MA)
Homogeneity	See `graycoprops()`, Matlab (Natick, MA)
Area	See `regionprops()`, Matlab (Natick, MA)
Centriod	See `regionprops()`, Matlab (Natick, MA)
Bounding Box	See `regionprops()`, Matlab (Natick, MA)
Major Axis Length	See `regionprops()`, Matlab (Natick, MA)
Minor Axis Length	See `regionprops()`, Matlab (Natick, MA)
Eccentricity	See `regionprops()`, Matlab (Natick, MA)
Orientation	See `regionprops()`, Matlab (Natick, MA)
Convex Area	See `regionprops()`, Matlab (Natick, MA)
Filled Area	See `regionprops()`, Matlab (Natick, MA)
Euler Number	See `regionprops()`, Matlab (Natick, MA)
Equivalent Diameter	See `regionprops()`, Matlab (Natick, MA)
Solidity	See `regionprops()`, Matlab (Natick, MA)
Extent	See `regionprops()`, Matlab (Natick, MA)
Perimeter	See `regionprops()`, Matlab (Natick, MA)
Pattern Matching	Compare two-input function to mean functions calculated for every S/N (binned by integer) in a training set. Record S/N at which pattern most closely fits according to least-squares.
Pattern Matching$_{i=S/N}$	Compare image to mean function calculated for S/N=i (binned by integer) in a training set. Record the sum-of-squares difference calculated over the two-input space.
Pattern Matching$_{Norm}$	Same as "Pattern Matching," but images (and the averages to which they are compared) are mean-centered and variance-normalized.

Table S6: Features used to analyze throttle behavior. These features were measured for each throttle phenotype (see Table S5), where "image" refers to the observed phenotype as a response to the two inputs, $A3$ and $AI3$ (see Figure S14).
