# Supplementary material for: Modular Design of Artificial Tissue Homeostasis: Robust Control through Synthetic Cellular Heterogeneity
Source: PLoS Comput Biol. 2012 Jul 19;8(7):e1002579. doi: 10.1371/journal.pcbi.1002579 (PMC3400602; doi:10.1371/journal.pcbi.1002579)
Supplement: Table S7 — Rate constants for two-compartment model of the UPC module. (PDF) [file pcbi.1002579.s026.pdf]

| Rate Label | Reaction |
|---|---|
| $k_0$ | Express pI1 |
| $k_1$ | Bind pI1.Rec1.AI1 |
| $k_2$ | Dissoc.pI1.Rec12.AI1 |
| $k_3$ | Decay.Rec1.AI1 (Same as Decay.Rec1) |
| $k_4$ | Express.pI1.Rec1.AI1 |
| $k_5$ | Synth.AI1 |
| $k_6$ | Decay.AI1 |
| $k_7$ | Bind.Rec1.AI1 |
| $k_8$ | Dissoc.Rec1.AI1 |
| $k_9$ | Express.pRec1 |
| $k_{10}$ | Decay.LuxI |
| $k_{11}$ | Decay.Rec1 |
| $k_{12}$ | Express.pA2 |
| $k_{13}$ | Express.pA2.Rec1.AI1 |
| $k_{14}$ | Decay.A2 |
| $k_{15}$ | Bind.pA2.Rec1.AI1 |
| $k_{16}$ | Dissoc.pA2.Rec1.AI1 |
| $k_{17}$ | AI1 diffusion across cell membrane |

**Table S7:** Rate constants for two-compartment model of the UPC module.
