# Supplementary material for: Modular Design of Artificial Tissue Homeostasis: Robust Control through Synthetic Cellular Heterogeneity
Source: PLoS Comput Biol. 2012 Jul 19;8(7):e1002579. doi: 10.1371/journal.pcbi.1002579 (PMC3400602; doi:10.1371/journal.pcbi.1002579)
Supplement: Table S8 — Top RS-HDMR identified throttle features and their corresponding RS-HDMR sensitivity indices, (see Figure 8 H ). (PDF) [file pcbi.1002579.s027.pdf]

S_i^T	**Phenotype/Rates**	**Feature Name**
0.55	R7 T to St. St.	Value at $A3 = 0.34$, $AI3 = 0.06$
0.16	Peak	Extent when image is thresholded at 90% max value
0.06	σ R7 T to St. St.	Bounding Box (corner of high $A3$, low $AI3$) when image is thresholded at 10% max value
0.06	No Switch Value	Pattern Matching
0.04	No Switch Value	Pattern Matching$_{Norm}$
0.03	H_{At}	
0.03	σ T to St. St.	Pattern Matching$_{Norm}$

Table S8: Top RS-HDMR identified throttle features and their corresponding RS-HDMR sensitivity indices, S_i^T (table supplements Figure 8H).
