# Supplementary material for: Modular Design of Artificial Tissue Homeostasis: Robust Control through Synthetic Cellular Heterogeneity
Source: PLoS Comput Biol. 2012 Jul 19;8(7):e1002579. doi: 10.1371/journal.pcbi.1002579 (PMC3400602; doi:10.1371/journal.pcbi.1002579)
Supplement: Table S9 — Summary of the advantages and disadvantages of Systems 1–4. (PDF) [file pcbi.1002579.s028.pdf]

	System 1	System 2	System 3	System 4
**Advantages**	• Simplicity<br>• Analytical solutions	• Intermediate simplicity<br>• Rapid feedback for reduced oscillations and improved homeostasis	• Oscillator generates population heterogeneity.<br>• Oscillator behavior and its ability to maintain homeostasis is well insulated from parameter values of other modules.<br>• Improves homeostasis when intercellular signaling is slow.	• Throttle generates population heterogeneity.<br>• Improves homeostasis when toggle switching times are slow.<br>• Works well for various intercellular signaling rates.<br>• Better overall performance relative to System 3 with intermediate molecular noise levels.
**Disadvantages**	• Poor population heterogeneity<br>• Undesired oscillations in β-cell population levels due to delayed feedback	• Poor population heterogeneity<br>• Highly dependent on reaction rates: requires rapid toggle switching and intercellular signaling.	• Reduced performance relative to System 4 with slow toggle switching and intercellular signaling.<br>• Requires significant molecular noise to operate well relative to System 4.	• Requires a third intercellular signal.<br>• Optimal performance requires parameter fine-tuning to match other modules.
