# Supplementary material for: Modular Design of Artificial Tissue Homeostasis: Robust Control through Synthetic Cellular Heterogeneity
Source: PLoS Comput Biol. 2012 Jul 19;8(7):e1002579. doi: 10.1371/journal.pcbi.1002579 (PMC3400602; doi:10.1371/journal.pcbi.1002579)
Supplement: Text S1 — Supporting text. Subsections include the following: (1) experimental proof of concept, (2) methods for the ODE modeling of Systems 1–2 and related analytical proofs, (3) methods for the Langevin modeling of Systems 1–2, (4) methods for the Gillespie modeling of Systems 2–3, (5) methods for results analyses of Systems 2–4, (6) additional results. (PDF) [file pcbi.1002579.s029.pdf]

Supporting text for
“Modular design of artificial tissue homeostasis: robust control through cellular heterogeneity”

Miles Miller[1†], Marc Hafner[1,2,3,4†,♯], Eduardo Sontag[5], Noah Davidsohn[1], Sairam Subramanian[6], Priscilla E. M. Purnick[7], Douglas Lauffenburger[1], and Ron Weiss[1,*]

1 Department of Biological Engineering, Massachusetts Institute of Technology, Cambridge, MA 02139, USA

2 School of Computer and Communication Sciences, Ecole Polytechnique Fédérale de Lausanne (EPFL), 1015 Lausanne, Switzerland

3 Department of Biochemistry, University of Zurich, 8057 Zurich, Switzerland

4 Swiss Institute of Bioinformatics, 1015 Lausanne, Switzerland

5 Department of Mathematics, Rutgers University, New Brunswick, NJ 08903, USA

6 Department of Chemical Engineering, Princeton University, Princeton, NJ 08544, USA

7 Department of Gene and Cell Medicine, Black Family Stem Cell Institute, Mount Sinai School of Medicine, New York, NY 10029, USA

† equal contribution

∗ E-mail: rweiss@mit.edu

Contents

1 Experimental proof of concept 2
- 1.1 Cell-cell communication . . . 2
- 1.2 Toggle switch . . . 2
- 1.3 Experimental methods . . . 2

2 Methods for the ODE modeling of Systems 1-2 and related analytical results 4
- 2.1 System 0: differentiation only . . . 4
- 2.2 Convergence and stability in Systems 1 and 2 . . . 4
- 2.3 Reduced two-population model . . . 5
- 2.4 Stability and convergence in the two-population model . . . 6
- 2.5 Robustness to the rate k_k . . . 11
- 2.6 Simulations . . . 12

3 Methods for the Langevin modeling of Systems 2-4 14
- 3.1 Quorum sensing module . . . 14
- 3.2 AND gate and toggle switch . . . 16
- 3.3 Cell fate . . . 16
- 3.4 System 3 – implementation of an oscillator . . . 17
- 3.5 System 4 – implementation of a throttle . . . 18
- 3.6 Spatial simulations . . . 18

4 Methods for the Gillespie modeling of Systems 2-3 20
- 4.1 Gene network design . . . 20

5 Methods for results analyses of Systems 2-4 23
- 5.1 RS-HDMR sensitivity analysis . . . 23
- 5.2 Langevin model analysis . . . 24
- 5.3 Analysis of a two-compartment ODE model of the UPC module . . . 25
- 5.4 Patterning and Neighbor Density Analysis . . . 28

6 Additional results 29
- 6.1 Detailed results for time-scale analysis . . . 29
- 6.2 Detailed results for the oscillator and throttle module analyses . . . 29
- 6.3 Population size when varying β-cell killing rate . . . 29

1 Experimental proof of concept

In recent years, synthetic biology efforts have produced a sizeable number of functional and characterized elements, ranging from repressors and activators to modules such as the toggle switch, oscillator, and cell communication systems. The Registry of Standard Biological Parts (http://partsregistry.org/) represents a collection of such elements [1, 2]. As a proof of concept, here we present experimental results for two critical components of the systems we aim to build. First, for cell-cell communications we engineered a mammalian receiver based on the LuxR protein that responds to 3-oxo-hexanoyl-homoserine lactone (3OC6HSL). Second, we employed two transcription factors, LacI and TetR, to create the toggle switch used in Systems 2-4 (Figures 3 and 4). The Weiss lab is currently developing two other modules needed for these systems: a mammalian 3OC6HSL sender based on LuxI, and a module to direct stem cell differentiation into insulin-producing pancreatic β-like cells. The differentiation module functions through stepwise expression of cell-fate regulators. *Gata4* expression in stem cells stimulates differentiation into endodermal cells, which activates an alpha-fetoprotein (AFP) promoter [3]. Preliminary results suggest that *ngn3* and *pdx1*, when fused to the AFP promoter, stimulate further differentiation into insulin-producing cells (data not shown).

1.1 Cell-cell communication

The mammalian receiver we built consists of a mammalian-optimized LuxR based signal transducer that binds 3OC6HSL and activates transcription from a novel mammalian optimized *lux* promoter (Figure S1A). We designed the signal transducer by fusing a p65 activation domain from the mammalian ReLa protein [4] to a helical linker H4 [5] and the N-terminus of a mammalian codon-optimized LuxRF, a hypersensitive LuxR mutant [6]. We also appended a nuclear localization signal (NLS) to the C-terminus of this protein. To test the redesigned receiver circuit (Figure S1A), HEK293FT cells were co-infected with a lentivirus constitutively expressing p65-H4-LuxRF/DsRed2 and a lentivirus with EGFP under PluxO7 control, demonstrating a highly functional mammalian 3OC6HSL receiver with an half maximal effective concentration (EC_{50}) of roughly 10μM (Figure S1B).

1.2 Toggle switch

Our toggle switch consists of two transcription factors, LacI and TetR (Figure S1C), that cross-repress each other. We fused a Krupple associated box (KRAB) domain to each of the LacI and TetR proteins to ensure efficient repression. The network design was based on earlier computational models [7] and an *E. coli* implementation [8]. Our experimental results indicate that the toggle switch state can be flipped with transient administration of IPTG and aTc. The system is able to maintain long term stability (>3 days), and the time required for the switch to reach 50% fluorescence is roughly 34 hours with the addition of aTc and 55 hours upon IPTG induction (Figure S1D).

1.3 Experimental methods

E. coli XL10-Gold cells were used to clone and propagate plasmids (Agilent; Santa Clara, CA). Cells were grown in LB broth (Difco, Detroit, MI) with 100μg/mL ampicillin (Sigma, St. Louis, MO) and 50 μg/mL kanamycin (Shelton Scientific, Shelton, CT) when appropriate. AHL 3-oxohexanoyl-homoserine lactone (3OC6HSL) was acquired from Sigma-Aldrich.

1.3.1 Mammalian cell culture

293-TetON (Clontech) cells were used to assay plasmids and viruses dependent on rtTA expression. NIH3T3 cells (ATCC) were used to assay viruses and determine their titer. Polybrene (Sigma) was used at a concentration of 10 μg/ml for infecting cells. All cells were grown at 37 °C and 5% CO2 in a sterile tissue culture incubator. Media for culturing 293FT/NIH3T3 cells was composed of DMEM (Hyclone), 10% Tet-approved Fetal Bovine

Serum (Clontech), 1% Penicillin-Streptomycin (Hyclone), and 0.1% Fungin (Invivogen) filtered through a 0.45 μ filter (Nalgene).

Lentivirus production and infection protocols were adapted from [9] using HEK293FT cells, packaging plasmids [10], and Superfect transfection reagent (Qiagen). Collected virus was concentrated either by ultrafiltration using Centricon Plus-70 100 kDa spin filters (Millipore) or by ultracentrifugation at 50000 g for 2.5 hours.

2 Methods for the ODE modeling of Systems 1-2 and related analytical results

In this section we discuss results related to analysis of the homeostasis system using ordinary differential equations (ODEs). We first describe the tissue homeostasis system using a four-population ODE model, but without any feedback control (termed "System 0"). We then show simulation results for the Systems 0, 1 (includes feedback) and 2 (includes toggle switch) with the four-population model and a corresponding two-population reduced model, which is equivalent to System 2. In the reduced system, we prove that equilibrium points exist under certain circumstances. We also show that the committed cell population remains robust to variations of the killing rate, k_k. The proofs are written in a general way and are valid for a broader scope than the present application.

2.1 System 0: differentiation only

The simplest tissue homeostasis system involves a mechanism that causes cells to differentiate, which we describe as the differentiation module. We model this system in terms of four cell types. Stem cells (population size is S) grow at a constant rate k_b and mature a constant rate k_{c1} into endodermic cells (E). Endodermic cells mature into pancreatic cells (P) at a rate k_{c2}. Finally, pancreatic cells differentiate at a rate k_d into β-cells (B), which then die at a constant rate k_k.

$$\begin{aligned}\frac{dS}{dt} &= k_b \cdot S - k_{c1} \cdot S \\ \frac{dE}{dt} &= k_{c1} \cdot S - k_{c2} \cdot E \\ \frac{dP}{dt} &= k_{c2} \cdot E - k_d \cdot P \\ \frac{dB}{dt} &= k_d \cdot P - k_k \cdot B \end{aligned} \tag{S1}$$

For this system, a non-zero equilibrium exists only if $k_b = k_{c1}$, for any sized equilibrium population $S_0 > 0$. Any deviation of $S_0 \cdot k_b/k_{c1}$ results in unabated proliferation or depletion of S. Moreover, S_0 and the equilibrium β-cell population ($B_0 = S_0 \cdot k_{c1}/k_k$) are sensitive to any deviation in k_{c1}/k_k.

We may also consider an external threshold on cell growth K_S, for example representative of nutrient limitations or contact inhibition:

$$\begin{aligned}\frac{dS}{dt} &= k_b \cdot S \frac{K_S}{K_S + S} - k_{c1} \cdot S \\ \frac{dE}{dt} &= k_{c1} \cdot S - k_{c2} \cdot E \\ \frac{dP}{dt} &= k_{c2} E - k_d \cdot P \\ \frac{dB}{dt} &= k_d \cdot P - k_k \cdot B \end{aligned} \tag{S2}$$

This system yields a non-zero stable equilibrium at $S_0 = \frac{k_b - k_{c1}}{k_{c1}} K_S$, and $B_0 = S_0 \cdot k_{c1}/k_k$, so long as $k_b > k_{c1}$. Even if the stem cell population may be controlled in this scenario, B nevertheless remains highly dependent on system parameters k_b, k_{c1}, and k_k. Such sensitive systems represent incomplete solutions to the problem of tissue homeostasis and are hardly ever observed *in vivo*; feedbacks ultimately remain critical components of a robust homeostasis system.

2.2 Convergence and stability in Systems 1 and 2

In System 1, the combination of a long delay (low values of k_{c1}, k_{c2} and k_d) and a nonlinear feedback (large n) induces undesirable oscillations. As discussed in the main text, reducing the delay in the feedback can

suppress the oscillations, but even if we engineer feedback within intermediate maturing populations (e.g. E), there realistically remains at least a two day delay. In System 2, including feedback through the toggle switch addresses this issue. We examined the difference between feedback control from either the β-cell population alone (System 1) or all committed cells together, i.e. endodermic, pancreatic and β-cells (System 2, see main text). Figure S2 demonstrates that including all committed cells in the feedback signal can further stabilize System 2 compared to System 1. To show the existence of a unique stable equilibrium point for System 2, we sampled all 625 combinations of five different initial values for each population ($S \in \{0.5, 1, 1.5, 2, 2.5\}$, $\{E, P, B\} \in \{0, 0.5, 1, 1.5, 2\}$) for 625 different parameter sets. All trajectories converged to the same equilibrium point for a given parameter set.

2.3 Reduced two-population model

We reduce the ODE model from a four-population to a two-population abstraction in order to simplify global stability analysis. We introduce the committed population as a variable $C = E + P + B$ and reduce the system to the two populations, S and C. Figure S2(third column) reveals consistent dynamics between the two- and four- population models under certain parameter sets. We can describe the system of four populations as the following:

$$\begin{aligned} \frac{dS}{dt} &= f_b(S) \cdot S - f_c(S, C) \cdot S \\ \frac{dE}{dt} &= f_c(S, C) \cdot S - k_{c2} \cdot E \\ \frac{dP}{dt} &= k_{c2} \cdot E - k_d \cdot P \\ \frac{dB}{dt} &= k_d \cdot P - k_k \cdot B \end{aligned} \tag{S3}$$

where $f_b(S)$ represents the control of stem cell division as a function of the number of stem cells and $f_c(S, C)$ represents the control of stem cell commitment as a function of the number of stem and committed cells. With $C = E + P + B$, the second equation of (S3) is reduced to:

$$\frac{dC}{dt} = \frac{dE}{dt} + \frac{dP}{dt} + \frac{dB}{dt} = f_c(S, C) \cdot S - k_k \cdot B \ . \tag{S4}$$

At steady state B, the β-cells population, can be expressed as the fraction of C. The following equations describe the partition of the committed cells for a given equilibrium point ($S_0, C_0 = E_0 + P_0 + B_0$):

$$\begin{aligned} E_0 &= \frac{k_d k_k}{k_{c2} k_d + k_{c2} k_k + k_d k_k} C_0 \\ P_0 &= \frac{k_k k_{c2}}{k_{c2} k_d + k_{c2} k_k + k_d k_k} C_0 \\ B_0 &= \frac{k_d k_{c2}}{k_{c2} k_d + k_{c2} k_k + k_d k_k} C_0 \end{aligned} \tag{S5}$$

Although the fractional composition of C with regards to E, P and β-cells may change dynamically (Figure S2), it remains a good approximation except for a short transient. Therefore, using the equilibrium populations (S5), the system (S3) can be written as

$$\begin{aligned} \frac{dS}{dt} &= f_b(S) \cdot S - f_c(S, C) \cdot S \\ \frac{dC}{dt} &= f_c(S, C) \cdot S - \overline{k_k} \cdot C \ , \end{aligned} \tag{S6}$$

where

$$\overline{k_k} = \frac{k_k k_d k_{c2}}{k_{c2} k_d + k_{c2} k_k + k_d k_k} \ . \tag{S7}$$

2.4 Stability and convergence in the two-population model

The following section proves the existence of equilibrium points in a general system with two populations and Hill-function feedbacks. With the relations in (S5), we can make a 1:1 correspondence between existing equilibrium points of the two- and four-population systems. Nevertheless, initial transient responses may differ between the models (see Figure S2).

In short, we prove that this system has a non-trivial stable equilibrium point when $k_b > k_d$. Moreover, at this equilibrium, we have that $S \geq K_S$ and $B \geq K_B$, provided that the parameters satisfy $\frac{k_d}{k_k} \geq 4\frac{K_B}{K_S}$. In the case when the condition $k_b > k_d$ fails, multiple non-trivial equilibria may exist; however, our system is monotone [11, 12], which insures global convergence to equilibrium even in that case. K_S and K_B control equilibrium population levels, and when $k_d > 4k_k$, $B = K_B$. Note that the analytical results suggest the necessity of having a nonlinear function for the feedback (Hill term), which could be biologically realized through cooperative binding of the signaling elements or a signal cascade.

2.4.1 Model and statements of results

We consider the following general system of two differential equations defined for $x = x(t) \geq 0$ and $y = y(t) \geq 0$:

$$\begin{aligned} \dot{x} &= f(x,y) &&= k_b[1-\theta_x(x)]x - k_d\theta_x(x)[1-\theta_y(y)]x \\ \dot{y} &= g(x,y) &&= k_d\theta_x(x)[1-\theta_y(y)]x - k_k y \end{aligned} \tag{S8}$$

where k_b, k_d, k_k are positive constants. In the reduced model that we are considering for tissue homeostasis, x is the stem cell population, y the committed cell population and the constants correspond respectively to the birth, differentiation and effective killing rates k_k. The continuously differentiable functions

$$\theta_x, \theta_y : [0,\infty) \to [0,1)$$

are assumed to satisfy:

$$\theta_x'(x) > 0 \text{ and } \theta_y'(y) > 0 \qquad \text{for all } x > 0,\, y > 0$$

and

$$\theta_x(1) = \theta_y(1) = \frac{1}{2}\,.$$

For the main conclusions, we will specialize to the normalized Hill functions:

$$\theta_x(x) = \frac{x^{n_x}}{1+x^{n_x}} \tag{S9}$$

and

$$\theta_y(y) = \frac{y^{n_y}}{1+y^{n_y}} \tag{S10}$$

where

$$n_x \geq 1\,, \quad n_y > 0\,. \tag{S11}$$

With these functions, and for large n_x and n_y, we have that $\theta_x(x) \approx 0$ if $x < 1$ and ≈ 1 if $x > 1$, and $\theta_y(y) \approx 0$ if $y < 1$ and ≈ 1 if $y > 1$. Thus, the first (growth) term in the definition of $\dot{x}$ in (S8) will be zero when the population x is larger than 1, while the second term, which represents the flux from the x to the y population, will be only nonzero if both $x > 1$ and $y < 1$. Intuitively, one would expect a homeostatic behaviour, which attempts to bring the value of y to a target of 1 while keeping the x population from extinction.

One could consider, more generally, functions θ_x and θ_y of the following form, with V_x, V_y, K_x, K_y not necessarily equal to 1:

$$\theta(s) = \frac{Vs^n}{K^n + s^n}\,,$$

and replace the terms $1-\theta(s)$ by $V-\theta(s)$. This more general situation corresponds to desired values of $x \approx K_x$ and $y \approx K_y$. We remark that the main conclusions also hold for this more general model. Indeed, (1) the

coefficients V_x and V_y can be absorbed into the constants k_b, k_d, k_k, and (2) rescaling x and y to, respectively, $K_x x$ and $K_y y$, we may take $K_x = K_y = 1$ without loss of generality, except that the parameter k_d in the equation for y has to be replaced by $k'_d = \frac{k_d K_S}{K_B}$. However, our results below only rely upon the algebraic form of the nullclines, the qualitative directions of the flow, and the location of the steady states. Such results remain invariant when multiplying the equation for y by the constant k_d/k'_d. Thus, we may assume $k_d = k'_d$, provided that we replace k_k by $\frac{k_k k_d}{k'_d} = \frac{k_k K_B}{K_S}$. Note, that for the same reasons, we could as well make $k_k = 1$, replacing k_b by k_b/k_k and k_d by k_d/k_k.

The equilibria are the points at which the x and y nullclines:

$$X = \{(x,y) \,|\, f(x,y) = 0\}\,, \quad Y = \{(x,y) \,|\, g(x,y) = 0\}$$

intersect. Note that there is always an equilibrium at $x = y = 0$. We are interested in nonzero equilibria.

The main results will be as follows; they are proved in the next section.

Lemma 2.1 The x nullcline X is the union of the line $x = 0$ and the graph of a strictly increasing and onto function

$$\psi \,:\, [\xi, \infty) \to [0, \infty)\,,$$

where

$$\xi \,:=\, \theta_x^{-1}\left(\frac{k_b}{k_b + k_d}\right)\,.$$

See Figure S3A; the arrows in the figure indicate the sign of the x-component of the vector field. Moreover:

- If θ_x is as in (S9) then $\xi \to 1$ as $n_x \to \infty$.
- $\xi \geq 1$ if and only if $k_b \geq k_d$.
- If θ_y is as in (S10) and $n_y > 1$, then ψ has a vertical tangent at ξ.
- If both θ_x is as in (S9) and θ_y is as in (S10), then for large x the function ψ has the asymptotic form:

$$y = \psi(x) \approx c_1 x^{\frac{n_x}{n_y}} \quad \text{with} \;\; c_1 = \left(\frac{k_d}{k_b}\right)^{\frac{1}{n_y}} \tag{S12}$$

Lemma 2.2 The y nullcline Y is the graph of a strictly increasing and onto function

$$\gamma \,:\, [0, \infty) \to [0, \infty)\,.$$

See Figure S3B; the arrows in the figure indicate the sign of the y-component of the vector field. Moreover:

- For all x:

  $$\gamma(x) \;\geq\; \min\left\{1, \frac{k_d}{2k_k}\theta_x(x)x\right\}$$

  and, in particular

  $$\gamma(x) \;\geq\; \min\left\{1, \frac{k_d}{4k_k}\right\} \qquad \text{for all } x \geq 1\,.$$

- If $k_b \geq k_d$, then

  $$\gamma(\xi) \;\geq\; \min\left\{1, \frac{k_d}{4k_k}\right\}\,.$$

- If both θ_x is as in (S9) and θ_y is as in (S10), then for large x the function γ has the asymptotic form:

$$y = \gamma(x) \approx c_2 x^{\frac{1}{n_y+1}}\,, \;\; \text{with} \;\; c_2 = \left(\frac{k_d}{k_k}\right)^{\frac{1}{n_y+1}}\,. \tag{S13}$$

These results suggest that the complete phase diagram is qualitatively as shown in Figure S3C. If there is a unique positive equilibrium, as in the figure, then the direction of the arrows shows that every trajectory starting from an initial condition with nonzero $x(0)$ must converge to that positive equilibrium.

However, the figure is misleading. Figure S3D shows a situation where more than one positive steady state exists. This example has θ_x as in (S9) and θ_y is in (S10), with $n_x = 2$, $n_y = 1$, and the following parameters: $k_b = 0.15$, $k_d = 4$, $k_k = 0.04$.

Thus, to prove that Figure S3C is indeed the correct picture, and global stability to a unique positive equilibrium holds, we need to impose some constraints on parameters.

Corollary 2.3 If the following condition is satisfied:

$$k_b \ \geq \ k_d \ \geq 4k_k \,, \tag{S14}$$

then every equilibrium point (x, y) different from $(0, 0)$ has the property that $x \geq 1$ and $y \geq 1$.

Corollary 2.4 If both θ_x is as in (S9) and θ_y is as in (S10), then there exists at least one positive equilibrium.

Corollary 2.5 In addition to the hypotheses of Corollary 2.4, suppose that $k_b \geq k_d$ and $n_x \geq 2$. Then, there is at most one positive equilibrium.

We summarize as follows.

Theorem 1 *Suppose that:*

- *θ_x is as in (S9) and θ_y is as in (S10),*
- *$n_x \geq 2$,*
- *$k_b \ \geq \ k_d$.*

Then, there is a unique positive equilibrium $(\bar{x}, \bar{y})$. All trajectories, except for those starting with $x(0) = 0$, converge to $(\bar{x}, \bar{y})$. Moreover, if also

- *$k_d \ \geq 4k_k$*

then $\bar{x} \geq 1$ and $\bar{y} \geq 1$. ∎

Remark. It is worth noting that, if θ_x is as in (S9) and θ_y is as in (S10), then as $n_x, n_y \to \infty$, the set Y takes the limiting form shown in Figure S3E.

To be precise, we show that, as $n \to \infty$, (1) for each fixed $x < 1$, $\gamma(x, n) \to 0$ and (2) for each fixed $x > 1$, $\gamma(x, n) \to 1$. To verify (1), we pick any $x < 1$. As $\theta_x(x) \to 0$ when $n_x \to \infty$, also $(k_d/k_k)\theta_x(x)x \to 0$; since $G^{-1}(0) = 0$, we conclude as claimed. To show (2), we pick $x > 1$, and pick y so that $G(y) = (k_d/k_k)\theta_x(x)x$. If $y > 1$, then $G(y) \to +\infty$ (because $\theta_y(y) \to 1$) as $n_y \to \infty$, so for large enough n_y, $G(y) > (k_d/k_k)\theta_x(x)x$, a contradiction. If instead $y < 1$, then $1 - \theta_y(y) \approx 1$, and thus (using that $k_d > k_k$, and thus also $(k_d/k_k)\theta_x(x)x \approx (k_d/k_k)x > 1$), as $n_x \to \infty$ we have that $y \approx G(y) \approx (k_d/k_k)x > 1$, contradicting the assumption $y < 1$. It follows that $y = 1$ in the limit, as claimed. This means that, for large Hill exponents, one may expect the value of y at nonzero steady states to be approximately 1. ∎

2.4.2 Proofs

Proof of Lemma 2.1

The set X is the union of the line $x = 0$ and the solution set of

$$k_b(1 - \theta_x(x)) - k_d\theta_x(x)[1 - \theta_y(y)] = 0 \tag{S15}$$

and we are interested in characterizing this latter solution set. We may rewrite the above equation as

$$1 - \theta_y(y) \;=\; \frac{k_b}{k_d}\frac{1 - \theta_x(x)}{\theta_x(x)} \;=\; \frac{k_b}{k_d}\left(\frac{1}{\theta_x(x)} - 1\right) . \tag{S16}$$

A solution $y = \psi(x)$ exists if and only if the right hand side is in the range $(0, 1]$, which amounts to saying (since

$$\frac{k_b}{k_d}\left(\frac{1}{\theta_x(x)} - 1\right) \;>\; 0$$

because $\theta_x(x) < 1$) that

$$\frac{k_b}{k_d}\left(\frac{1}{\theta_x(x)} - 1\right) \;\leq\; 1 .$$

This property is equivalent to $\frac{1}{\theta_x(x)} - 1 \leq \frac{k_d}{k_b}$, that is,

$$\theta_x(x) \;\geq\; \frac{k_b}{k_b + k_d} = \theta_x(\xi)$$

which is the same as asking $x \geq \xi$. Thus $y = \psi(x)$ is defined for $x \geq \xi$. As both the left and right-hand sides of (S16) are strictly decreasing functions of their arguments, it follows that ψ is increasing, and it is clearly differentiable by the same reasoning. Note that $\psi(\xi) = 0$, by definition of ξ, and that $\psi(x) \to \infty$ as $x \to \infty$, because $\theta_x(x) \to 1$ as $x \to \infty$, which implies that the right-hand side of (S16) converges to zero, and thus $\theta_y(y) \to 1$.

The direction of the vector field is clear from the fact that, for any fixed y, the expression

$$\frac{1}{\theta_x(x)} - 1 - \frac{k_d}{k_b}\,[1 - \theta_y(y)]$$

is positive when x is very small and negative when x is very large.

Suppose that θ_x is as in (S9), and use here a subscript n_x to indicate its dependence on n_x. Pick any $\varepsilon > 0$. There is uniform convergence $\theta_{x,n_x}(z) \to 0$ for $z \leq 1 - \varepsilon$ and $\theta_{x,n_x}(z) \to 1$ for $z \geq 1 + \varepsilon$ as $n_x \to \infty$. Therefore, for each fixed number $\eta \in (0, 1)$, it follows that $1 - \varepsilon < \theta_{x,n_x}^{-1}(\eta) < 1 + \varepsilon$ Since ε was arbitrary, this means that $\theta_{x,n_x}^{-1}(\eta) \to 1$ as $n_x \to \infty$. In particular, applied to $\eta = \xi$, we have that $\xi \to 1$ as $n_x \to \infty$.

Observe that, $k_b \geq k_d$ if and only if $\frac{k_b}{k_b+k_d} \geq \frac{1}{2} = \theta_x(1)$. Thus, since θ_x is an increasing function, $k_b \geq k_d$ if and only if $\xi = \theta_x^{-1}(\frac{k_b}{k_b+k_d}) \geq \theta_x^{-1}(\theta_x(1)) = 1$, as claimed.

We next show that if θ_y is as in (S10) and $n_y > 1$, then ψ has a vertical tangent at ξ. Taking implicit derivative with respect to x and then a limit as $x \searrow \xi$ and $y \searrow 0$ in (S16), we have that:

$$\psi'(x) \;\to\; \frac{1}{\theta_y'(0)}\,\frac{k_b}{k_d}\,\frac{\theta_x'(\xi)}{\theta_x(\xi)^2}$$

as $x \searrow \xi$, and therefore this limit is $+\infty$ if $\theta_y'(0) = 0$.

The asymptotic form of the nullcline, for large x, when both θ_x is as in (S9) and θ_y is as in (S10), is shown as follows. The equality in (S16) becomes:

$$\frac{1}{1 + y^{n_y}} \;=\; \frac{k_b}{k_d}\frac{1}{x^{n_x}}$$

which means that

$$\psi(x) \;=\; \left(\frac{k_d}{k_b}x^{n_x} - 1\right)^{\frac{1}{n_y}} \;\approx\; c_1 x^{\frac{n_x}{n_y}}$$

with $c_1 = \left(\frac{k_d}{k_b}\right)^{\frac{1}{n_y}}$.

This completes the proof of Lemma 2.1. ∎

Proof of Lemma 2.2

The y nullcline set Y consists of the solutions of

$$G(y) = \frac{y}{1-\theta_y(y)} = (k_d/k_k)\theta_x(x)x\,.$$

The function G is continuous and strictly increasing (because θ_y is strictly increasing), $G(0) = 0$, and $G(y) \to \infty$ as $y \to \infty$. Therefore G is invertible, and thus Y is the graph of the strictly increasing function

$$y = \gamma(x) = G^{-1}((k_d/k_k)\theta_x(x)x)$$

which clearly satisfies that $\gamma(0) = 0$. As $x \to \infty$, $\theta_x(x) \to 1$, so $\theta_x(x)x \to \infty$, which implies that $\gamma(x) \to \infty$ as well.

The direction of the vector field is clear from the fact that

$$g(x,y) \;=\; k_d\theta_x(x)[1-\theta_y(y)]x - k_k y \;>\; 0$$

if and only if

$$G(y) \;<\; (k_d/k_k)\theta_x(x)x$$

which is the same as $y < \gamma(x)$, and that the expression is < 0 if and only if $y > \gamma(x)$.

We claim that:

$$\gamma(x) < 1 \quad \text{if and only if} \quad \gamma(x) > \frac{k_d}{2k_k}\theta_x(x)x\,.$$

Indeed, suppose that $y = \gamma(x)$, that is $\frac{y}{1-\theta_y(y)} = (k_d/k_k)\theta_x(x)x$. Note that $y < 1$ is equivalent to $\theta_y(y) < 1/2$, which is the same as $\frac{y}{1-\theta_y(y)} < 2y$. Thus

$$y < 1 \quad \text{if and only if} \quad 2y > \frac{k_d}{k_k}\theta_x(x)x$$

as claimed. It follows that

$$\gamma(x) \;\geq\; \min\left\{1, \frac{k_d}{2k_k}\theta_x(x)x\right\}\,.$$

In particular, when $x \geq 1$, $\theta_x(x) \geq 1/2$, so $\theta_x(x)x \geq 1/2$ and therefore $\frac{k_d}{2k_k}\theta_x(x)x \geq \frac{k_d}{4k_k}$

If in addition $k_b \geq k_d$, then $\xi \geq 1$ by Lemma 2.1, and thus $\gamma(\xi) \geq \min\left\{1, \frac{k_d}{4k_k}\right\}$.

Finally, suppose that θ_x is as in (S9) and θ_y is as in (S10). We have that $y = \gamma(x)$ must satisfy:

$$y + y^{n_y+1} = \frac{k_d}{k_k}\frac{x^{n_x+1}}{1+x^{n_x}}$$

When $x \to \infty$, also $y \to \infty$, so $\frac{k_d}{k_k}\frac{x^{n_x+1}}{1+x^{n_x}} \approx \frac{k_d}{k_k}x$ and $y + y^{n_y+1} \approx y^{n_y+1}$. Therefore, $y \approx c_2 x^{\frac{1}{ry+1}}$ with $c_2 = \left(\frac{k_d}{k_k}\right)^{\frac{1}{ry+1}}$ as $x \to \infty$ (and so also $y \to \infty$). ∎

Proof of Corollary 2.3

When $k_b \geq k_d$, Lemma 2.1 insures that $\xi \geq 1$ and Lemma 2.2 insures that $\gamma(\xi) \geq \min\left\{1, \frac{k_d}{4k_k}\right\}$. Thus, if also $k_d \geq 4k_k$, it follows that $\gamma(\xi) \geq 1$. Given the forms of the nullclines for x and y, any equilibrium should have $x \geq \xi \geq 1$. Thus $y = \gamma(x) \geq \gamma(\xi) \geq 1$. ∎

Proof of Corollary 2.4

By Lemma 2.1, the function ψ that describes the nontrivial branch of the x nullcline is defined for $x \geq \xi > 0$, with $\psi(\xi) = 0$, and has the asymptotic form $\psi(x) \approx c_1 x^{\frac{n_x}{n_y}}$. By Lemma 2.2, the function γ that describes the y nullcline has $\gamma(0) = 0$ and has the asymptotic form $\gamma(x) \approx c_2 x^{\frac{1}{n_y+1}}$. To show a nonzero intersection between the graphs of these two functions, it suffices to know that $\psi(x) > \gamma(x)$ for some x. This is clear because, for some positive constant c:

$$\frac{\psi(x)}{\gamma(x)} \approx c\,\frac{x^{\frac{n_x}{n_y}}}{x^{\frac{1}{n_y+1}}} = c\,x^{\frac{n_x}{n_y}-\frac{1}{n_y+1}} \to \infty \quad \text{as } x \to \infty$$

since $\frac{n_x}{n_y} - \frac{1}{n_y+1} > 0$ (using $n_x \geq 1$ and $n_y > 0$). ∎

Proof of Corollary 2.5

We must show that there is only one intersection of the zero sets of

$$k_b(1 - \theta_x(x)) = k_d\theta_x(x)[1 - \theta_y(y)]$$

and

$$k_d\theta_x(x)[1 - \theta_y(y)]x = k_k y\,.$$

We can equally well replace the second equation by:

$$k_b(1 - \theta_x(x))x = k_k y\,.$$

Thus, it is enough to show that the graphs of these two functions:

$$\alpha(x) = \frac{k_b}{k_k}(1 - \theta_x(x))x$$

$$\beta(x) = \theta_y^{-1}\left(1 - \frac{k_b}{k_d}\left[\frac{1}{\theta_x(x)} - 1\right]\right)$$

(defined for $x \geq \xi$, where, as before, $\theta_x(\xi) = \frac{k_b}{k_b+k_d}$) intersect at only one point. Since β is a strictly increasing function defined for $x \geq \xi \geq 1$ (this last inequality uses that $k_b \geq k_d$), with $\beta(0) = 0$ and $\alpha(x) > 0$ for all x, it is sufficient to show that $\alpha'(x) \leq 0$ for $x \geq 1$. Indeed, $[1 - \theta_x(x)]x = \frac{x}{1+x^{n_x}}$, so $\alpha'(x)$ vanishes only at $x = \bar{x} = (n_x - 1)^{-1/n_x}$ and is negative for $x > \bar{x}$. Since $n_x \geq 2$, $n_x - 1 \geq 1$, from which it follows that $\bar{x} \leq 1$. Thus, as required, $\alpha'(x) \leq 0$ for $x \geq 1 \geq \bar{x}$. ∎

2.5 Robustness to the rate k_k

We present an informal argument to estimate the order of the dependence of the steady state on the degradation rate k_k of y, when all other parameters are kept constant. This is an important property of the homeostasis system as y, the committed cell population should be independent of external perturbations. We assume that θ_x is as in (S9) and θ_y is as in (S10) and that we are in the asymptotic regime. The nullclines have the forms in (S12)-(S13):

$$\psi(x) \approx c_1 x^{\frac{n_x}{n_y}} \quad \text{with } c_1 = \left(\frac{k_d}{k_b}\right)^{\frac{1}{n_y}}$$

$$\gamma(x) \approx c_2 x^{\frac{1}{n_y+1}}\,, \quad \text{with} \;\; c_2 = \left(\frac{k_d}{k_k}\right)^{\frac{1}{n_y+1}}\,.$$

At equilibrium, $\psi(x) = \gamma(x)$ gives that x has order ${k_k}^p$ and therefore $y = c_1 x^{\frac{n_x}{n_y}}$ has order ${k_k}^q$, where

$$p = \frac{n_y}{n_y - n_x n_y - n_x}\,, \quad q = \frac{n_x}{n_y - n_x n_y - n_x}\,.$$

If $n_x = n_y = n$, these simplify to $p = q = -1/n$. For example, with $n_x = n_y = 4$, we expect that x and y will grow like ${k_k}^{-1/4}$.

2.6 Simulations

We show next nullclines and simulations for these values:

$$k_b = 3,\; k_d = 2,\; n = 4,$$

and k_k varying by 3 orders of magnitude:

$$k_k = 0.001, 0.01, 0.1, 1\,.$$

Shown below are simulations for $x(t)$ and $y(t)$ (with initial states $x(0) = 0.3$, $y(0) = 0$), followed by the respective x and y nullclines, for values of k_k respectively as above. Observe the very weak dependence of the steady state values $\bar{x}$ and $\bar{y}$ on the rate k_k, consistently with their order being ${k_k}^{-1/4}$, since $0.001^{-1/4} \approx 5.6$, $0.01^{-1/4} \approx 3.16$, $0.1^{-1/4} \approx 1.78$, and $1^{-1/4} = 1$.

<u>$k_k = 0.001$**:**</u>

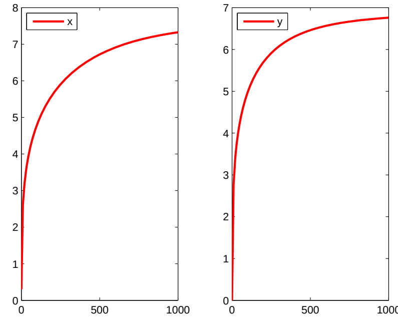

$x(t)$ and $y(t)$

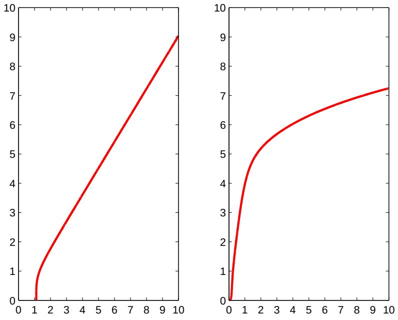

x and y nullclines

<u>$k_k = 0.01$**:**</u>

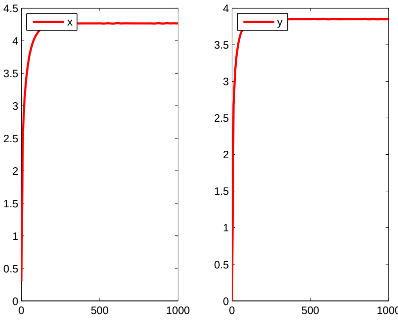

$x(t)$ and $y(t)$

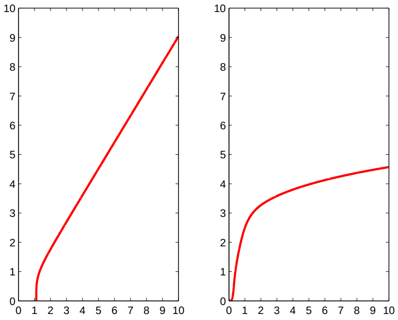

x and y nullclines

<u>$k_k = 0.1$**:**</u>

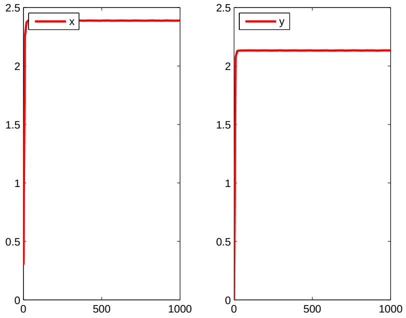

$x(t)$ and $y(t)$

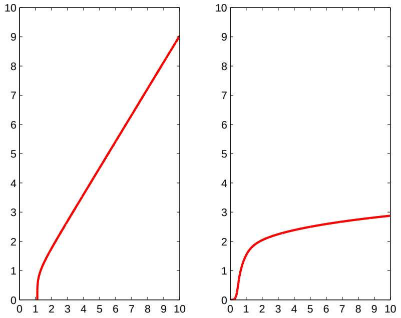

x and y nullclines

<u>$k_k = 1$**:**</u>

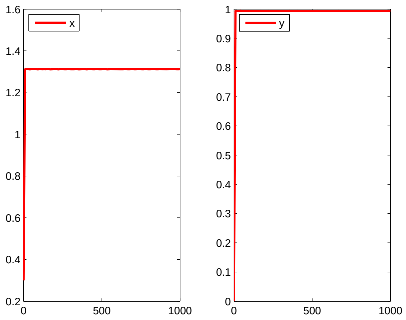

$x(t)$ and $y(t)$

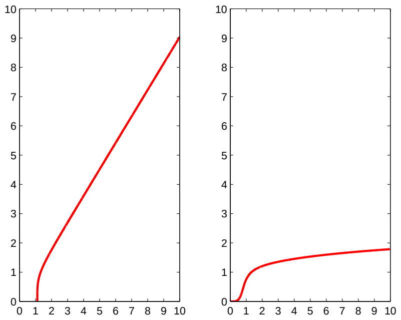

x and y nullclines

3 Methods for the Langevin modeling of Systems 2-4

We perform chemical Langevin simulations [13] of the various system designs (Figures 3D-F, 4) and use these implementations to perform various analyses (Figures 5, 7 and 8). We add modulated white noise to each reaction at each integration step, with noise amplitude controlled by the parameter Ω. We refer to Ω as 'cell volume', because it relates concentration to molecular count. For example, a volume of 200 denotes that a particular molecule's concentration of 1 (which is the average value for most of the active components in our models) corresponds to a total of 200 molecules per cell. All the equations in the following are written as ODE to lighten the notation, but are simulated as chemical Langevin equations [15]. For example, for an ODE system written as

$$\frac{d\,\mathbf{X}}{dt} = \sum_{j=1}^{m} \boldsymbol{\nu}_j r_j(\mathbf{X}) \ ,$$

where $\mathbf{X}$ is the concentration of the different species, $\boldsymbol{\nu}_j$ the stoichiometric vector corresponding to the j-th column of the stoichiometric matrix and r_j the rate function of the j-th reaction. The increment of $\mathbf{X}$ for a time interval τ using the corresponding chemical Langevin equation will be

$$\mathbf{X}(t+\tau) = \mathbf{X}(t) + \sum_{j=1}^{m} \boldsymbol{\nu}_j r_j(\mathbf{X})\,\tau + \sum_{j=1}^{m} \boldsymbol{\nu}_j \sqrt{\frac{r_j(\mathbf{X})}{\Omega}\,\tau}\, N_j \ ,$$

where $N_j \sim \mathcal{N}(0,1)$ is a normal random variable with mean 0 and variance 1. Note that for the calculus of $r_j(\mathbf{X})$, we used a multiple state procedure as described in [14] for a higher precision.

We aim to optimize the systems such that the number of committed cells remains constant. We define the objective function as the signal to noise ratio S/N of the fraction of committed cells ρ_c in a simulation of duration T:

$$S/N = \frac{\overline{\rho_c}}{\sqrt{1/T \int_0^T \left(\rho_c(t) - \overline{\rho_c}\right)^2 dt}} \qquad \text{where} \quad \overline{\rho_c} = 1/T \int_0^T \rho_c(t)dt \tag{S17}$$

Note that the fraction of committed cells is similar in all systems due to the quorum sensing module (see Sec. 3.1) and therefore, the S/N value is not biased by large difference in $\overline{\rho_c}$ between different systems. S/N measurement begins after simulations have been allowed to somewhat equilibrate (generally after 500 hours of simulation).

The following sections detail the equations for Systems 2 to 4. Table S1 summarizes the parameter values for the three systems. We approximate the evolution of each component in each model (Figures 3-4) to follow a Hill kinetic with a coefficient of $n = 4$. k_p^α and H_α denote the maximum rate and half-rate constant, respectively, for the component α. Degradation follows mass-action kinetics (rate k_d^α) for all components. Finally, we describe diffusion as a linear function (rate k_{diff}) of the difference between internal and external (AI) concentrations. We constrain the maximum number of cells in the simulation (N_{max}) to be less than 150.

3.1 Quorum sensing module

In Systems 2-4, uncommitted cells signal through $AI1$. The toggle switch, comprised of $R6$ and $R7$ cross-inhibition, regulates $I1$ expression. $I1$ subsequently controls $AI1$ production. $R7$, which is produced only in committed cells, inhibits $AI1$ production. In contrast, $R6$, which is produced only in uncommitted cells, inhibits the signal for committed cells ($AI2$). We approximate $AI1$ and $AI2$ as being directly dependent upon $R6$ and

$R7$, and we describe the concentration of these components according to the following equations:

$$\frac{d\ AI1}{dt} = k_p^{AI1}\frac{H_{AI1}^n}{H_{AI1}^n + R7^n} - k_d^{AI1}AI1 + k_{\text{diff}}(AI1_{out} - AI1) \quad \text{(S18)}$$

$$\frac{d\ AI2}{dt} = k_p^{AI2}\frac{H_{AI2}^n}{H_{AI2}^n + R6^n} - k_d^{AI2}AI2 + k_{\text{diff}}(AI2_{out} - AI2) \quad \text{(S19)}$$

The following equations describe the homogeneous extracellular AI concentration:

$$\frac{d\ AI1_{out}}{dt} = -\sum_{Cells}\frac{k_{\text{diff}}}{N_{max}}(AI1_{out} - AI1_{Cells}) - k_d^{AI1}AI1_{out} \quad \text{(S20)}$$

$$\frac{d\ AI2_{out}}{dt} = -\sum_{Cells}\frac{k_{\text{diff}}}{N_{max}}(AI2_{out} - AI2_{Cells}) - k_d^{AI2}AI2_{out} \quad \text{(S21)}$$

The equilibrium concentrations in uncommitted cells of $AI1$ and $AI2$, $\overline{AI1}$ and resp. $\overline{AI2}$, for given population sizes ρ_u and ρ_c can be evaluated assuming that the production rate in eq. (S18) and (S19) is either zero or maximal ($k_p^{AI\alpha}$) depending on the state of each cell. If the size of both cell populations is expressed as a fraction of N_{max}: $\rho_u = \frac{N_u}{N_{max}}$ for uncommitted cells and $\rho_c = \frac{N_c}{N_{max}}$ for the committed cells, $\overline{AI1}$ and $\overline{AI2}$ can be written as:

$$\overline{AI1}(\rho_u,\rho_c) = \frac{k_p^{AI1}}{k_d^{AI1}}\frac{(k_d^{AI1}+k_{\text{diff}})(k_d^{AI1}+\rho_u k_{\text{diff}}) + \rho_c k_{\text{diff}} k_d^{AI1}}{(k_{\text{diff}}+k_d^{AI1})(k_{\text{diff}}+k_d^{AI1}+\rho_u k_{\text{diff}}+\rho_c k_{\text{diff}})} \quad \text{(S22)}$$

$$\overline{AI2}(\rho_u,\rho_c) = \frac{k_p^{AI2}}{k_d^{AI2}}\frac{\rho_c k_{\text{diff}}^2}{(k_{\text{diff}}+k_d^{AI2})(k_{\text{diff}}+k_d^{AI2}+\rho_u k_{\text{diff}}+\rho_c k_{\text{diff}})} \quad \text{(S23)}$$

The production of the component $A1$ (or for System 4, the repressor $R1$) is controlled by the receptor $Rec1$ to which $AI1$ binds. Similarly, $AI2$ binds to $Rec2$ and activates the production of the repressor $R2$. In this model, we simplify the expressions of $A1/R1$ and $R2$ as depending directly on the concentration of $AI1$ or $AI2$ following a Hill-type equation. In all systems, the half-rate constants for the production terms of $A1/R1$ and $R2$ within the population control modules, is adjusted to trigger the cell-decision processes for a threshold of the fraction of uncommitted cells ρ_u around 0.45 and of committed cells ρ_c around 0.4. It means that the half-rates are equal to $\overline{AI1}(\rho_u = 0.45, \rho_c = 0.4)$ for $A1/R1$ production and $\overline{AI2}(\rho_u = 0.45, \rho_c = 0.4)$ for $R2$ production as defined above. Therefore,

$$\frac{d\ A1}{dt} = k_p^{A1}\frac{AI1^n}{H_{A1}^n + AI1^n} - k_d^{A1}A1 \quad \text{with} \quad H_{R1} = \overline{AI1}(0.45, 0.4) \quad \text{for Systems 2 and 3} \quad \text{(S24)}$$

$$\frac{d\ R1}{dt} = k_p^{R1}\frac{H_{R1}^n}{H_{R1}^n + AI1^n} - k_d^{R1}R1 \quad \text{with} \quad H_{R1} = \overline{AI1}(0.45, 0.4) \quad \text{for System 4} \quad \text{(S25)}$$

$$\frac{d\ R2}{dt} = k_p^{R2}\frac{H_{R2}^n}{H_{R2}^n + AI2^n} - k_d^{R2}R2 \quad \text{with} \quad H_{R2} = \overline{AI2}(0.45, 0.4) \quad \text{(S26)}$$

3.2 AND gate and toggle switch

The AND gate integrates the information from the two quorum sensing modules (and the oscillator or the throttle in Systems 3 and 4, respectively). The AND gate in Systems 3 and 4 include an activator $A3$, and System 3 also includes an additional repressor ($R4$). $R5$ serves as the output and interacts with downstream modules (e.g., the toggle switch in Systems 2-4). The System 2 AND gate only contains $R5$, which we describe by the following equation:

$$\frac{d\ R5}{dt} = k_p^{R5} \frac{A1^n}{H_{R5-1}^n + A1^n} \frac{H_{R5-2}^n}{H_{R5-2}^n + R2^n} - k_d^{R5} R5 \tag{S27}$$

In System 3, the AND gate includes two additional elements ($A3$ and $R4$):

$$\frac{d\ A3}{dt} = k_p^{A3} \frac{A1^n}{H_{A3-1}^n + A1^n} \frac{H_{A3-4}^n}{H_{A3-4}^n + R4^n} - k_d^{A3} A3 \tag{S28}$$

$$\frac{d\ R4}{dt} = k_p^{R4} \frac{H_{R4}^n}{H_{R4}^n + Ro2^n} - k_d^{R4} R4 \tag{S29}$$

$$\frac{d\ R5}{dt} = k_p^{R5} \frac{A3^n}{H_{R5-3}^n + A3^n} \frac{H_{R5-2}^n}{H_{R5-2}^n + R2^n} - k_d^{R5} R5 \tag{S30}$$

In System 4, the throttle acts on $R5$ through the signaling molecule $AI3$. Furthermore, System 4 includes $A3$:

$$\frac{d\ A3}{dt} = k_p^{A3} \frac{H_{A3-1}^n}{H_{A3-1}^n + R1^n} \frac{H_{A3-2}^n}{H_{A3-2}^n + R2^n} - k_d^{A3} A3 \tag{S31}$$

$$\frac{d\ R5}{dt} = k_p^{R5} \frac{A3^n}{H_{R5-3}^n + A3^n} \frac{H_{R5-t}^n}{H_{R5-t}^n + AI3^n} - k_d^{R5} R5 \tag{S32}$$

The toggle switch consists of the two repressors, $R6$ and $R7$, which inhibit each other. Cells initially have the toggle in the uncommitted state, with high $R6$ and low $R7$. $R5$ controls toggle switching by inhibiting $R6$. The equations for $R6$ and $R7$ are the following:

$$\frac{d\ R6}{dt} = k_p^{R6} \frac{H_{R6-5}^n}{H_{R6-5}^n + R5^n} \frac{H_{R6-7}^n}{H_{R6-7}^n + R7^n} - k_d^{R6} R6 \tag{S33}$$

$$\frac{d\ R7}{dt} = k_p^{R7} \frac{H_{R7}^n}{H_{R7}^n + R6^n} - k_d^{R7} R7 \tag{S34}$$

The equation for $R7$ differs for System 4, and is discussed below.

3.3 Cell fate

We describe uncommitted cells as potentially proliferative and immortal. In contrast, committed cells cannot grow but rather die. We model the control of proliferation in uncommitted cells using growth arrest factor

(GAF), which is regulated by quorum sensing activity. We model differentiation as potentiated by the expression of a transcription factor such as $GATA4$. Once above a certain threshold, $GATA4$ initiates slow cell death as approximated by a kinetic model described below.

3.3.1 Division process

GAF is controlled by $A1/R1$ (quorum sensing of the uncommitted cells):

$$\begin{aligned} \frac{d\ GAF}{dt} &= k_p^{GAF}\frac{A1^n}{H_{GAF}^n + A1^n} \\ &\quad -k_d^{GAF}GAF \qquad \text{for Systems 2 and 3} \qquad \text{(S35)} \\ \frac{d\ GAF}{dt} &= k_p^{GAF}\frac{H_{GAF}^n}{H_{GAF}^n + R1^n} \\ &\quad -k_d^{GAF}GAF \qquad \text{for System 4} \qquad \text{(S36)} \end{aligned}$$

The cell grows if GAF lies below a threshold th_{GAF}. To model growth, we use an integrator for the division depending on GAF level,

$$\frac{d\ Div}{dt} = k_b\Theta(th_{GAF} - GAF) - \frac{k_b}{3}\Theta(GAF - th_{GAF})$$

with $Div = 0$ at the time at cell division and Θ represents the Heaviside function ($\Theta(x) = 0$ if $x < 0$ and $\Theta(x) = 1$ if $x \geq 0$). k_b denotes the division rate. We choose $k_b = \frac{1}{96}h^{-1}$, such that the average time for division (in absence of GAF), is set to $96h$. Division occurs when $Div(t) \geq 1$. The two daughter cells inherit all concentrations of the mother cell's components except Div, which is reset to zero.

3.3.2 Commitment process

$R6$ inhibits $GATA$, and $GATA$ production begins when the toggle switches to low $R6$:

$$\begin{aligned} \frac{d\ GATA}{dt} &= k_p^{GATA}\frac{H_{GATA}^n}{H_{GATA}^n + R6^n} \\ &\quad -k_d^{GATA}GATA \qquad \text{(S37)} \end{aligned}$$

When $GATA$ concentration reaches a threshold, the cell becomes differentiated and no longer proliferates. We describe the lifetime of committed cells by the following:

$$\frac{d\ Death}{dt} = k_k$$

with $Death = 0$ at the time of differentiation and k_k denotes the death rate. A cell dies when $Death$ reaches 1. We choose $k_k = \frac{1}{200}h^{-1}$, such that the average time for the death of a β-cell is set to $200h$.

3.4 System 3 – implementation of an oscillator

To break symmetry between individual cells in the population, oscillations are introduced through a relaxation oscillator with a design similar to previous experimental studies in prokaryotic and eukaryotic cells [16, 17, 18, 19, 20, 21]. The simplest possible oscillator is made of a component Ao that activates itself (autopositive feedback) and regulates the expression of a repressor Ro that inhibits Ao. Two successive components integrate oscillator dynamics with the AND gate. A second repressor $Ro2$ acts on $R4$, which in turn represses commitment. With proper parameter values, this system generates short intervals of low $R4$, with an irregular latency where $R4$

concentration remains high. The equations of the oscillator are as follows:

$$\frac{d\ Ao}{dt} = k_p^{Ao}\left(k_0^{Ao} + \frac{Aosc^n}{H_{Ao-A}^n + Ao^n}\right)\frac{H_{Ao-R}^n}{H_{Ao-R}^n + Ro^n} - k_d^{Ao} Aosc \tag{S38}$$

$$\frac{d\ Ro}{dt} = k_p^{Ro}\frac{Ao^n}{H_{Ro}^n + Ao^n} - k_d^{Ro} Ro \tag{S39}$$

$$\frac{d\ Ro2}{dt} = k_p^{Ro2}\frac{Ao^n}{H_{Ro2}^n + Ao^n} - k_d^{Ro2} Ro2 \tag{S40}$$

Linking the oscillator and the quorum sensing module to the toggle switch occurs in two steps as described above. The element $A3$ is controlled by both $A1$ and $R4$, therefore $A3$ is produced only when $A1$ is high (enough uncommitted cells) and $R4$ is low.

3.5 System 4 – implementation of a throttle

We implement a throttle design involving a third quorum sensing molecule, $AI3$, as another mechanism to inhibit the simultaneous commitment of cells in a population. We design the activator At to control the production of $AI3$, such that $AI3$ is produced transiently when the toggle is switching. $AI3$ diffuses through the membrane and gives rise to an external concentration $AI3_{out}$ that enters other cells. The throttle equations are as follows:

$$\frac{d\ At}{dt} = k_p^{At}\frac{H_{At-6}^n}{H_{At-6}^n + R6^n} - k_d^{At} At \tag{S41}$$

$$\frac{d\ AI3}{dt} = k_p^{AI3}\frac{At^n}{H_{AI3-t}^n + At^n}\frac{H_{AI3-7}^n}{H_{AI3-7}^n + R7^n} - k_d^{AI3} AI3 + k_{\text{diff}}(AI3_{out} - AI3) \tag{S42}$$

$$\frac{d\ AI3_{out}}{dt} = -\sum_{Cells}\frac{k_{\text{diff}}}{N_{max}}(AI3_{out} - AI3_{Cells}) - k_d^{AI3} AI3_{out} \tag{S43}$$

The remaining elements are similar to System 2, except for two key differences: first, the addition of $A3$ (see Eq. S31 and S32), and second, the control of $R7$ by At instead of $R6$:

$$\frac{d\ R7}{dt} = k_p^{R7}\frac{At^n}{H_{R7}^n + At^n} - k_d^{R7} R7 \tag{S44}$$

3.6 Spatial simulations

We implement spatially resolved multicellular simulations using the Langevin model to analyze spatiotemporal commitment patterns (discussed further in Supplementary Text 5.4). We represent the spatial distribution of individual cells in a manner similar to that employed in the Gillespie-simulations (discussed in the following section). The extracellular volume of the system is divided into a 6x5x5 grid with $N_{max} = 150$ boxes, each with the same volume as a cell. Each cell occupies one box on the grid. Diffusion can occur between the cell and its box or between the boxes. As diffusion is physically faster than any other process in the cell, we simulate

it as an ODE without the Langevin noise term. Diffusion occurs from one edge to edge (periodic boundary conditions) in order to mimic a larger system and avoid border effects. A dividing cell can push neighboring cells into the nearest empty box; therefore, daughter cells are adjacent at time of division.

4 Methods for the Gillespie modeling of Systems 2-3

In addition to the Langevin model, we implement more mechanistically detailed models of Systems 2 and 3 that do not make quasi-steady-state assumptions for many of the network components. We use these models primarily for analysis of hysteresis in the UPC module (see section *Clustered sensitivity analysis* in the main text), but also use the Gillespie models to confirm several results observed in the Langevin simulations (including patterning analysis). We use a standard rate-equation approach and monitor the spatiotemporal evolution of cells, proteins, and signaling molecules with a Gibson-modified Gillespie algorithm. We describe the full tissue homeostasis system as a set of discrete stochastic reactions occurring in cells using a previously described multicellular spatiotemporal simulation environment [22, 23]. The simulation platform tracks the temporal evolution of intracellular reactions within individual cells that grow on a 2D grid, as well as the spatiotemporal evolution of the cells themselves and extracellular signaling molecules that diffuse among them. We model cell growth as a stochastic buildup of a species "Volume" that triggers cell division upon reaching a threshold. We model growth inhibition as the binding of GAF to a "Volume" precursor, thereby inhibiting "Volume" accumulation. "Volume" levels divide with cell division, and newly created cells form adjacent to their parents on the 2D grid. If needed, neighboring cells are "pushed" to adjacent grid positions in order to make room for newly divided cells.

4.1 Gene network design

The overall designs of Systems 2 and 3 are generally similar to the Langevin implementations in network topology (Figure 3-4) but contain more details about the receptor and the UPC model (Figure S4).

We model repressors and activators binding to a gene's promoter, thus changing transcription and translation rates. Repressors such as TetR-KRAB fusion protein and activators such as VP16-AraC fusion protein fit this model and have been previously implemented in mammalian synthetic genetic networks [24]. Our model of engineered cell-cell communication in a mammalian system is based on bacterial two-gene QS systems, such as *rhlI/rhlR* in *Pseudomonas (P.) aeruginosa* [25] and *luxI/luxR* in *Vibrio (V.) fischeri* [26]. QS systems have previously been used in synthetic gene networks to engineer cell-cell communication in both bacteria [27] and mammalian cells [28]. Generally, one protein (LuxI or RhlI, modeled generically as I1 and I2) catalyzes synthesis of a freely diffusing small molecule, known as an autoinducer (AI), specific to a particular QS system (modeled here as $AI1$ and $AI2$). A receptor protein (LuxR or RhlR, modeled generically as $Rec1$ and $Rec2$) binds the appropriate intracellular autoinducer. The resulting complex acts as an activating transcription factor similar to activators described above, and is engineered to reach high concentration when population density reaches a "QS threshold." Other artificial signaling pathways, for example those previously engineered in yeast [29], may also be implemented. As with the Langevin models, the proposed circuit design can be described in terms of six key modules: "Uncommitted Population Control" (UPC), the "Oscillator", the "Committed Population," the "AND" gate module, the "Toggle Switch" module, and the "Differentiation" module (see Figure 4A). Model reactions and rates are listed in Table S3.

The following section discusses the genes that comprise these modules and qualitatively discusses system dynamics. Each of the fourteen individual genes comprising the genetic modules belong to one of five categories: quorum sensing (QS) genes, repressors, activators, growth arrest factor, and cell-fate regulators. As discussed in the main part of the paper, repressors and activators are referred to generically as (Ro, $Ro2$,...) and (Ao, $A3$,...). QS molecular species adapted to a mammalian host from gram-negative bacterial communication are referred to as LuxR homologues ($Rec1$ and $Rec2$), LuxI homologues ($I1$ and $I2$), and cognate autoinducers ($AI1$ and $AI2$).

4.1.1 The UPC Module

In the UPC module, population control of uncommitted cells is controlled through a cell-cell communication system involving two proteins, $I1$ and $Rec1$. I1 catalyzes synthesis of $AI1$, which diffuses freely across cell membranes and acts as an intercellular biochemical signal. $AI1$ binds $Rec1$, a receptor protein. The resultant

complex is an activating transcription factor ($Rec1.AI1$) that can bind specifically engineered promoters of various genes [30]. In our system, $Rec1.AI1$ binds the promoter of $I1$, causing activated transcription of that gene. The positive feedback in $I1$ expression produces nonlinear response to increasing $AI1$, ultimately leading to a step-like function in $Rec1/I1$ expression as population density increases. Within the UPC module, $Rec1.AI1$ binds the promoter for growth arrest factor (GAF) and activates its transcription when uncommitted population density is above the QS threshold. Once cells commit to differentiation via switching of the toggle switch (discussed later), expression of $I1$ is inhibited by $R7$. This repression allows $I1$ expression, and consequently $AI1$ concentration, to be reflective only of uncommitted population density.

4.1.2 The Committed Population

With the switch to commitment and to high $R7$ expression, gene $I2$ is no longer repressed by $R6$. $I2$ catalyzes synthesis of $AI2$, which binds $Rec2$ to form the complex $Rec2.AI2$. $Rec2.AI2$ activates both $I2$ and $Rec2$ expression through a double-positive feedback mechanism. As with the $Rec1/I1$ QS system used in the population control module, positive feedback creates a sharp gain in $AI2$ production as the population density of committed cells increases.

4.1.3 Symmetry Breaking Oscillator

To break symmetry between individual cells in the population, oscillations are introduced through a relaxation oscillator with a design similar to previous experimental studies in prokaryotic and eukaryotic cells [16, 17, 18, 19, 20, 21]. *Ao* activates expression of itself and two repressors, *Ro* and *Ro*2. *Ro* in turn represses *Ao* expression. *Ro* and *Ro*2 are activated as *Ao* levels increase. *Ro* subsequently represses further expression of *Ao* and, as *Ao* levels decrease, *Ro* expression is inactivated. As *Ro* levels fall to a sufficiently low level (due to inactive transcription), *Ao* again is freely expressed. *Ro* is modeled as a slowly degrading protein to maximize oscillation period, and *Ro*2 is designed as a relatively fast decaying protein to minimize periods of high *Ro*2.

4.1.4 The AND Gate

The AND gate module regulates the commitment of individual cells to differentiate. $Rec1.AI1$ interfaces with the commitment module through binding the $A3$ promoter and activating $A3$ expression. $A3$ expression is high only when the uncommitted population is high ($Rec1.AI1$ is bound to the $A3$ promoter at high levels) and the oscillator peaks (*Ao* is high). *Ro*2 output from the oscillator (high when *Ao* is high) represses $R4$, which in turn represses $A3$. Even when bound by $Rec1.AI1$, $A3$ expression is repressed when $R4$ is bound. $A3$ activates expression of the repressor $R5$. $R5$ subsequently interacts directly with the toggle switch. The "committed population" module interfaces with the AND module through $Rec2.AI2$ activation of *Ro*2. *Ro*2 represses $R5$ expression regardless of whether $A3$ is bound to the $R5$ promoter. *Ro*2 is high when the population of committed cells is high, thereby repressing further commitment. $R5$ expression is activated only when (1) uncommitted population density is high, (2) the oscillator is high, and (3) committed population density is low.

4.1.5 The Toggle Switch

The toggle switch defines whether an individual cell is uncommitted or committed. The bi-stable toggle switch consists of two mutually inhibitory repressors: $R6$ and $R7$. High expression of one repressor inhibits expression of the other. The switch is initially set to high $R6$ levels in uncommitted cells. This can be accomplished in engineered systems through the introduction of an inducer that inactivates $R7$. For example, if $R7$ was LacI, transient addition of IPTG would stably set the toggle switch to high $R6$. When the AND module output is high, $R5$ represses $R6$ expression, thus allowing $R7$ to be expressed. $R7$ then represses $R6$, stabilizing the toggle switch in the high $R7$ state. To increase the bi-stability of the switch, multiple repressor binding sites are incorporated into each of the promoters of the switch. The transition from high $R6$ to high $R7$ expression in the toggle switch signifies commitment to differentiation. $R7$ represses $I1$ expression in the UPC module, causing repression of $AI1$ expression.

4.1.6 The Differentiation Module

With the switch to commitment, subsequently low $R6$ levels allow differentiation to occur through unrepressed expression of cell fate regulators. For differentiation into pancreatic β-cells, for instance, the unrepressed cell fate regulator could be an endoderm-directing cell fate regulator such as Gata4. In order to describe Gata4 as inducing differentiation into endoderm cells, cells are modeled as having a one way toggle switch involving the factors that sustain either the undifferentiated or differentiated state. As a cell-fate regulator, Gata4 is modeled as inducing the switch from the undifferentiated to differentiated state. Because differentiated cells exhibit significantly lower growth rates and higher death rates as compared to stem cells, differentiated cells are modeled as accumulating killer protein ("E") and being unable to grow. When killer protein levels reach a given threshold, the cell dies.

Differentiation may be successfully directed if guided by stepwise expression of various cell-fate regulators at critical points in the differentiation pathway. Promoters have been identified which are only active in certain cell types. For example, the Alpha-FetoProtein promoter (pAFP) is only active in cells that have differentiated into endoderm cells. Such promoters could be used for the sequential expression of key transcription factors at specific points along the differentiation pathway. As described in this circuit, the pancreas specific cell-fate regulator genes *pdx1* and *ngn3* are fused downstream of pAFP. Thus once the cell reaches the endoderm stage, *pdx1* and *ngn3* expression is activated, and differentiation is further directed into a β-cell fate.

5 Methods for results analyses of Systems 2-4

5.1 RS-HDMR sensitivity analysis

RS-HDMR is a tool to deduce non-linear interactions between a set of inputs and an output [31]. In this work, we use RS-HDMR in multiple distinct applications. RS-HDMR describes the independent and cooperative effects of n parameters $\mathbf{x} = (x_1, x_2, ...x_n)$ on an output, $y = f(\mathbf{x})$, in terms of a hierarchy of RS-HDMR component functions:

$$f(\mathbf{x}) = f_0 + \sum_{i=1}^{n} f_i(x_i) + \sum_{1 \leq i < j \leq n} f_{ij}(x_i, x_j) + ... + f_{12...n}(x_1, x_2, ...x_n) \tag{S45}$$

Here f_0 represents the mean value of $f(\mathbf{x})$ over the sample space, the first-order component function $f_i(x_i)$ describes the generally non-linear independent contribution of the ith input variable to the output, the second-order component function $f_{ij}(x_i, x_j)$ describes the pairwise cooperative contribution of x_i and x_j, and further terms describe higher order cooperative contributions. In this work, we generally consider first-, second-, and third-order RS-HDMR component functions. We approximate RS-HDMR component functions as weighted orthonormal basis functions, which take the following form:

$$f_i(x_i) \approx \sum_{r=1}^{k} \alpha_r^i \varphi_r^i(x_i) \tag{S46}$$

where k is an integer (generally ≤ 3 for most applications), $\{\alpha\}$ are constant weighting coefficients to be determined, and the basis functions $\{\varphi\}$ are optimized from the distribution of sample data points to follow conditions of orthogonality [31]. Basis functions are approximated here as non-linear polynomials, where

$$\varphi_1^i(x_i) = a_1 x_i + a_0 \qquad \varphi_2^i(x_i) = b_2 x_i^2 + b_1 x_i + b_0 \qquad \varphi_3^i(x_i) = c_3 x_i^3 + c_2 x_i^2 + c_1 x_i + c_0 \tag{S47}$$

The coefficients a_0,a_1,b_0,...c_3 are calculated using Monte Carlo integration under constraints of orthogonality, such that when integrated over all data points,

$$\int \varphi_r(x)dx \approx 0 \ \ \forall\, r \qquad \int \varphi_r^2(x)dx \approx 1 \ \ \forall\, r \qquad \int \varphi_p(x)\varphi_q(x)dx \approx 0 \ \ (p \neq q) \tag{S48}$$

Optimal basis functions are weighted by coefficients (α_r^i), which are calculated from least-squares regression. Only inputs and their respective component functions measured as significant by the statistical F-test were included in RS-HDMR expansions [32]. The resultant expansion in Eq. S45 serves both as a predictive model of network response due to its parametric interactions and as a statistical representation of the underlying system.

The relative strength of response to parametric changes can be quantitatively determined through sensitivity analysis based on the respective RS-HDMR component functions. A global sensitivity analysis may be calculated from the RS-HDMR expansion through a decomposition of the total variance σ^2 of an output species, $f(x)$, into hierarchical contributions from the individual RS-HDMR component functions. For each RS-HDMR expansion, the total sensitivity/variance σ of the output $f(\mathbf{x})$ is decomposed into hierarchical contributions $(\sigma_i, \sigma_{i,j}, \ldots)$ from the individual RS-HDMR component functions of the remaining input variables:

$$1 = \sum_{i=1}^{n} S_i + \sum_{1 \leq i < j \leq n} S_{ij} + ... + S_\epsilon \tag{S49}$$

In Eq. (51), $S_i = \sigma_i^2/\sigma^2$ is defined as the sensitivity index of the corresponding RS-HDMR component function, $f_i(x_i)$. $S_{ij} = \sigma_{i,j}^2/\sigma^2$ is the sensitivity index of the corresponding second-order component function, $f_{ij}(x_i, x_j)$. S_ϵ is the sensitivity index of the residual variation of the model. The collection of sensitivity indices S_i,

$\sum_{j\neq i}^{n} S_{ij}$, $\sum_{j\neq i}^{n}\sum_{k\neq i,j}^{n} S_{ijk}$ corresponding to first, second, and third order component functions of the input variable x_i can then be summed into an index $S_i^T(i = 1, 2...n)$, describing both independent and higher-order effects of x_i on an output. The magnitudes of $S_i^T(i = 1, 2, ..., n)$ can be used to quantify the relative interaction strength between the outputs and the inputs.

5.2 Langevin model analysis

5.2.1 Time-scale optimization

Time-scale optimization involves multiplying rate constants by a scalar TS parameter while preserving the ratio of closely related rates (see Table S1). We scale the rate k_p (production rate), k_d (degradation rate) and k_{diff} (diffusion rate) of all components in the quorum signaling module by a factor $TS_{QS} \in [\frac{1}{3}, 3]$. We perform analogous scaling for the quorum sensing module ($TS_{QM} \in [\frac{1}{3}, 3]$). For the commitment module, we independently analyze three individual components. $TS_{R5} \in [\frac{1}{3}, 3]$ scales k_p^{R5} and k_d^{R5}. $TS_{R6} \in [\frac{1}{3}, 3]$ and $TS_{R7} \in [\frac{1}{3}, 3]$ denote analogously lumped parameters. For System 4, we include an additional parameter for the dynamics of the component At ($TS_{At} \in [\frac{1}{3}, 3]$). For Systems 2 & 3, we analyze five total time-scale parameters. We analyze six time-scale parameters in System 4 (see Table S2). We randomly sample the time-scale space from a log-uniform distribution over one order of magnitude for each parameter, ultimately generating 360 independent parameter sets (Figure S5). For each time-scale set, we average the observed S/N over eight simulations. We perform RS-HDMR analysis to map the input-output relationships between the time-scale parameters (inputs) and their corresponding S/N, the output (Figure 5C-F). We excluded 30% of the dataset to cross-validate RS-HDMR inference and used the remaining 70% of the data as the training set. RS-HDMR inference results indicated consistent fitting accuracy between the test and training sets.

5.2.2 Robustness to variations of molecular noise amplitude

To test the influence of stochastic fluctuations in the models, we changed the volume from 40 to 1400 as shown in Figures 5A and S6. For each value of Ω, 24 simulations are performed and the average S/N value is recorded. Error bars in these plots indicate standard deviation of the results for the 24 simulations.

5.2.3 Robustness to variation of the killing rate k_k

To test the influence of the killing rate in the models, we change the value of k_k from 45 to 550 hours (corresponding to a ratio k_b/k_k of 0.5 to 6) as shown in Figures 5B and S6. For each value of k_k, 24 simulations are performed and we record the average S/N value and the average committed population density. Error bars in these plots indicate standard deviation of the results for the 24 simulations.

5.2.4 Module optimization

We randomly and independently modified twelve parameters involving the oscillator in System 3 (Figure 7A), and nine parameters involving the throttle in System 4 (Figure 7G), to understand the impact of parameter variation on module properties and, ultimately overall S/N. We randomly sampled the parameter space from a log-uniform distribution, one order of magnitude around the nominal values for each parameter (see Table S1). We generated 2000 independent parameter sets for the oscillator and roughly 6000 parameter sets for the throttle. For each parameter set, we simulated system behavior with the Langevin models and recorded the observed S/N value (average of 16 independent simulations). We also performed simulations where the module is isolated from the system and focus on different phenotypes of the module. For System 3, we isolate the oscillatory module (Figure 7A) and record the properties for the $R4$ component (output of the module) as described in Figure 8A and Table S4. For System 4, we isolate the throttle (Figure 7G) by analyzing the toggle response ($AI3$ and $R7$ concentrations) to independently (exogenously) modulated inputs of $A3$ (signal from the QS modules) and $AI3$ (signal from adjacent cells), as shown in Figure 8F. For the isolated throttle, produced

$AI3$ is kept separate from the input of $AI3$ and therefore does not act on $R5$. We focus on changes in $R7$ and $AI3$ concentrations in response to exogenously controlled combinations of $A3$ and $AI3$ as inputs (see Table S5). For the oscillator, each tested parameter set yields a single value for each phenotype. In contrast, we test the isolated throttle with combinations of different $A3$ and $AI3$ inputs. Therefore, for each throttle parameter set, we obtain a 2-dimensional grid for each phenotype with 11 sampled values of $A3$ $(0.07, 0.16, \ldots, 0.97)$ and 18 sampled values for $AI3$ $(0.06, 0.12, \ldots, 1.08)$. These input values were chosen based on $A3$ and $AI3$ concentrations observed in the full system simulations.

We performed several types of analysis on both Systems 3 and 4 to understand the relationships between the module rate constants, the module phenotypes, and corresponding S/N of the full system (Figures 7, 8 and S7-S14). We perform RS-HDMR analysis using either rate constants, module phenotypes, or both as inputs to describe the system output, S/N. Because throttle phenotypes are described as a function of two inputs ($A3$ and $AI3$), we describe the two-input functions ("images") of each phenotype (see Figures S13-S14) as a set of features (see Table S6). For features extracted using `regionprops()`, images were thresholded at various levels, digitized accordingly, and then analyzed. `regionprops()` calculates properties such as "Centroid," and "Filled Area" for the region of an the image falling above a particular threshold; we used both relative thresholds, such as $> 90\%$ max value, and absolute thresholds for this analysis. For features extracted using `graycoprops`, images were first converted to gray-level co-occurrence matrices. Overall, we extracted roughly 10,000 features from the original 16 phenotype images. We performed partial least squares regression (PLSR, `plsregress()`, Matlab, Natick, MA) using the 10,000 features as inputs, and ranked features by their variable importance in the projection (VIP) [33]. The 20 most significant variables were then analyzed by RS-HDMR. Table S4 shows the most significant features identified by RS-HDMR. Even with the reduced number of features, RS-HDMR inference performs with roughly equal or better accuracy compared to partial least squares regression in all cases tested.

We used Bayesian network analysis to produce graphical representations of the conditional probabilistic dependencies of the module rate-constants and phenotypes on each other and on overall S/N. Directed graph structures produced by Bayesian network inference consist of "nodes", which in this application are the module phenotypes and S/N, and "edges", which represent conditional probabilistic relationships between the nodes. Bayesian network inference was performed as described previously [34]. Briefly, we derived consensus directed graph structures from exact Bayesian network model averaging over all directed acyclic graph (DAG) structures having at most four parents per node [35, 36]. Consensus networks for Systems 3 and 4 (Figure 9) only include those edges with a score > 0.8 and > 0.3, respectively, where an edge score of 0.8, for example, denotes that 80% of the derived Bayesian networks tested over all iterations of optimization include that edge. Although our implementation of Bayesian inference cannot capture cyclical feedback, bi-directional edges can be observed with model averaging when using a significance threshold below 0.5. The bi-directional edges reported in System 4 (Figure 9B) arise from observation of both edge directions above the 0.3 threshold. For System 3, we ranked phenotypes and rate-constants by their VIP score after performing PLSR as referred to above, and analyzed the top 15 most significant rate-constants and phenotypes using Bayesian inference. For System 4, we analyzed the 9 most significant module phenotypes (as determined by their VIP), along with all 9 module rate-constants. For both systems, we only considered DAGs with directed edges from module phenotypes to the "S/N" node (i.e., we defined module phenotypes as strictly upstream of S/N), and we specified rate-constants as strictly upstream of both the module phenotypes and S/N. From these constraints, we consequently inferred directionality from the remaining edges. We iteratively removed nodes that were not upstream of S/N in the consensus network, and recalculated the consensus network structure. As a result, the final network structures are comprised of S/N and only those nodes upstream of S/N at the chosen significance threshold. Prior to network inference, nodes were discretized using two, three, and four-level k-means clustering with squared Euclidean distance metric and 50 iterations. Bayesian inference results across the three types of k-means clustering were then averaged. Software used for Bayesian network inference has been previously described [35].

5.3 Analysis of a two-compartment ODE model of the UPC module

We implement the full model (Systems 2 and 3) as a system of discrete stochastic reactions using an established multicellular, spatiotemporal simulation platform [22, 23] (see section 4). Because computational costs of

running this platform are high, we can turn to a simpler simulation framework for high-throughput sampling and optimization of rate-constants within individual subnetworks of the full system model. We employ a deterministic, two-compartment ordinary differential equation (ODE) model of the UPC module for efficient genetic algorithm (GA) optimization and sensitivity analysis of a portion of the full system stochastic model. (See the following page for specific equations and a table of rate constants.) Intracellular reactions of the ODE system use identical rate constants to those in the stochastic model, with the standard correction for dimerization reactions [37]. We approximate individual cells within the population as identical to reduce the complexity of the ODE system. We model diffusion as occurring between a variable population density and a homogeneous extracellular volume, thus neglecting the effect of spatial signaling gradients. Using k_{diff} as the rate of diffusion across a cell membrane, k_c as the rate of AI decay, and ρ_p as the population density of cells in the culture, concentration of AI outside of the cell ($[AI_{out}]$) is described by the following equation:

$$\frac{d\,[AI_{out}]}{dt} = \rho_p k_{diff}\left([AI_{in}] - [AI_{out}]\right) - k_c\,[AI_{out}] \tag{S50}$$

where $[AI_{in}]$ represents the concentration of AI within the cells. As with the stochastic simulator, cell density and concentration of species within cells are approximated as uniform. In the ODE model, we also approximate the concentration of extracellular AI as homogeneous. We describe diffusion in and out of individual cells as independent of population density (at least explicitly, by exclusion of ρ_p), such that

$$\frac{d\,[AI_{in}]}{dt} = \sum_i f_{in}(\mathbf{x}) + k_{diff}\left([AI_{out}] - [AI_{in}]\right) - k_c\,[AI_{in}] \tag{S51}$$

where $\sum_i f_{in}(\mathbf{x})$ represents the sum of all $i = 12$ intracellular reactions described on the following page and in Table S6.

$$
\begin{aligned}
\frac{d\,[pI1]}{dt} &= k_2\,[pI1.Rec1.AI1] + k_3\,[pI1.Rec1.AI1] - k_1\,[pI1]\,[Rec1.AI1] \\
\frac{d\,[pRec1]}{dt} &= 0.0 \\
\frac{d\,[LuxI]}{dt} &= k_0\,[pI1] + k_4\,[pI1.Rec1.AI1] - k_{10}\,[LuxI] \\
\frac{d\,[Rec1]}{dt} &= k_9\,[pRec1] - k_{11}\,[Rec1] - k_7\,[Rec1]\,[AI1_{in}] + k_8\,[Rec1.AI1] \\
\frac{d\,[AI1_{in}]}{dt} &= -k_7\,[Rec1]\,[AI1_{in}] + k_8\,[Rec1.AI1] + k_5\,[LuxI] \\
&\quad -k_6\,[AI1_{in}] + k_{17}([AI1_{out}] - [AI1_{in}]) \\
\frac{d\,[Rec1.AI1]}{dt} &= k_7\,[Rec1]\,[AI1_{in}] - k_8\,[Rec1.AI1] - k_3\,[Rec1.AI1] \\
&\quad +k_2\,[pI1.Rec1.AI1] - k_1\,[pI1]\,[Rec1.AI1] \\
&\quad -k_{15}\,[pA2]\,[Rec1.AI1] + k_{16}\,[pA2.Rec1.AI1] \\
\frac{d\,[pI1.Rec1.AI1]}{dt} &= -k_2\,[pI1.Rec1.AI1] + k_1\,[pI1]\,[Rec1.AI1] - k_3\,[pI1.Rec1.AI1] \\
\frac{d\,[pA2]}{dt} &= -k_{15}\,[pA2]\,[Rec1.AI1] + k_{16}\,[pA2.Rec1.AI1] + k_3\,[pA2.Rec1.AI1] \\
\frac{d\,[pA2.Rec1.AI1]}{dt} &= k_{15}\,[pA2]\,[Rec1.AI1] - k_{16}\,[pA2.Rec1.AI1] - k_3\,[pA2.Rec1.AI1] \\
\frac{d\,[A2]}{dt} &= k_{12}\,[pA2] + k_{13}\,[pA2.Rec1.AI1] - k_{14}\,[A2] \\
\frac{d\rho_p}{dt} &= k_{34} \\
\frac{d\,[AI1_{out}]}{dt} &= k_{17}\rho([AI1_{in}] - [AI1_{out}]) - k_6\,[AI1_{out}]
\end{aligned}
$$

5.3.1 Genetic algorithm

For the GA optimization, parameter vectors in initial generations consist of random points within biologically reasonable ranges of parameter space. To calculate the "forward QS response" in the ODE model, initial $\rho_p = 0$ and ρ_p increases at rate k_{34} that is much slower than other reactions in the system, such that module output, defined as $[pA2.Rec1.AI1]$, maintains quasi-steady-state. The "reverse QS response" in the ODE model is similarly calculated, but using the initial value $\rho_p = 1$, and having the population decrease at rate $d\rho_p/dt = -k_{34}$. The cost function for the GA defines the forward and reverse responses of $[pA2.Rec1.AI1]$ as a function of ρ_p to be a least-squares fit to a three component step function, described in the main text. Over 1000 generations (100 individual parameter vectors to a generation), system behavior evolves from a relatively flat response to a more optimal digital-like step function response.

5.3.2 RS-HDMR analysis of hysteresis

We use RS-HDMR to understand the impact of parametric variation on system performance in the two-compartment model of the UPC module. Input-output relationships are defined as the effects of parametric variation on hysteresis of the UPC module response to fluctuations in population density. More specifically, we define hysteresis as the difference between the forward and reverse response values of population density (ρ_p), where the UPC module's output ($[pA2.Rec1.AI1]$) is 50% of maximum, or (max output + min output)/2. We focus on absolute levels of hysteresis rather than normalizing to the average population density threshold, be-

cause we focus on systems with similar average thresholds and consider absolute changes in population density to be a relevant optimization feature of our system in the context of its biomedical application.

We performed RS-HDMR sensitivity analysis on datasets describing neighborhoods of parameter space around optimal parameter vectors obtained from GA runs. We generated two sets of 75 GA optimizations: the first set considered both the forward and reverse QS responses to changing population density, and the second set only considered forward response. Random sampling around each optimal parameter vector was from a normal distribution $N(\mu, \sigma)$ where μ is the optimized parameter's value and $\sigma = \mu/20$. Empirical evidence suggested that significantly broader sampling resulted in too many parameter sets that did not yield QS behavior. Sample size of the training set was 2000, and the resultant model was tested on unsampled points for validation purposes. In this application, we only considered the first-order RS-HDMR component functions in order to perform efficient high-throughput analyses of local parameter "neighborhoods."

5.4 Patterning and Neighbor Density Analysis

We analyzed distances between pairs of committed and uncommitted cells to identify patterning between the two cell-types. For a given committed or uncommitted reference cell, the ratio of committed to uncommitted neighbors at a given distance was calculated for all distances. We define $p(c)_{i,d,t}$ as the observed fraction of committed cell neighbors at distance d for the ith cell at time t. The normalized score $Z(c)_{i,d,t}$ for that observation is then described by

$$Z(c)_{i,d,t} = \frac{p(c)_{i,d,t} - \mu_t}{\sigma_{i,t}} \tag{S52}$$

where μ_t represents the overall fraction of committed cells at time t and $\sigma_{i,t}$ describes the standard deviation of the observed probability given by the standard form:

$$\sigma_{i,t} = \sqrt{\frac{\mu_t(1-\mu_t)}{n_{i,t}}} \tag{S53}$$

where $n_{i,t}$ is the number of total neighbors observed for the ith cell at time t. Normalized Z-scores are combined into an average Z-score, $\overline{Z(c)_d}$, for each distance value,

$$\overline{Z(c)_d} = \sum_i \sum_t Z(c)_{i,d,t}/\sqrt{N_d} \tag{S54}$$

where N_d is the total number of sample Z-scores $Z(c)_{i,d,t}$ for each distance. In Figures S18 and S19, the "Z-score" metric refers to $\overline{Z(c)_d}$.

6 Additional results

6.1 Detailed results for time-scale analysis

With the results of the time-scale analysis (see the section *Intermodular time-scale* in main text), we selected individually optimized values for Systems 2–4. The optimized parameter values below are an average of the 10% top performing parameter sets tested.

	TS_{QS}	TS_{QM}	TS_{R5}	TS_{R6}	TS_{R7}	TS_{At}
System 2	2.31	1.14	1.29	1.67	1.24	–
System 3	2.14	0.91	1.08	1.84	1.11	–
System 4	1.95	0.88	1.38	1.54	0.72	1.01

Such parameter optimization increases system performance and robustness to variations in the killing rate k_k and the cell volume Ω (Figure S6). On average, the optimization affords a gain of 5 units for the S/N value in all systems for all conditions. The major qualitative difference is the ability of the time-scale optimized System 4 to function even with low molecular noise ($\Omega > 1000$) in a comparable way to System 3. Results can be compared to Figure 5A-B in the main text.

6.2 Detailed results for the oscillator and throttle module analyses

We employ RS-HDMR to predictively model the relationship between module rate-constants, phenotypes, and overall system performance (see Figures 7 and 8). RS-HDMR infers and predicts precise S/N values with little accuracy when only the module rate-constants are employed as predictive variables (Figure S10A,C). $R^2 \approx 0.5$ for the analysis of both Systems 3 (Figure 8E) and 4 (Figure 8I). This relatively poor fit arises from a highly uneven distribution of observed S/N values (>50% of parameter sets have S/N<2) and what are likely to be significant higher (greater than third) order RS-HDMR component functions, which we do not account for in this application. Nonetheless, RS-HDMR can classify system performance as 'good' or 'bad' based on the rate-constants alone with an area under the ROC-curve (AUROC) of 0.97 (we define 'good' performers as parameter sets with observed S/N $>$ 15 and 'bad' ones as parameter sets with S/N $<$ 2). When module phenotypes are used to classify system performance (Figure S10B,D) in the same manner, RS-HDMR predicts with AUROC>0.98 for both Systems 3 (Figure 8E) and 4 (Figure 8I). Prediction with both the rate-constants and module phenotypes marginally improves this accuracy to an AUROC>0.99 for both systems. This trend in accuracy mirrors the R^2 values reported Figure 8E,I, where inference using module phenotypes generally out-performs inference using rate-constants alone. To compare RS-HDMR classification accuracy with another algorithm, we also implemented SVM classification using MATLAB (R2009a, The MathWorks, Natick, MA), with a two-norm soft-margin SVM classifier and linear kernel. For both Systems 3 and 4, RS-HDMR outperforms SVM in classification accuracy in the three scenarios (rate-constants only, phenotypes only, and rate-constants and phenotypes combined).

6.3 Population size when varying β-cell killing rate

In the previous section describing the ODE model (section 2), we found three important results concerning the sensitivity of the population to variations of the β-cell killing rate (k_k). First, the population of uncommitted cells is well controlled and remains constant even for low ratio of division rate over killing rate (k_b/k_k). Second, for high ratio, the population of committed cells follows a power law with an exponent $1/n$ where n is the Hill coefficient in the feedback function. Third, for low ratios, on the contrary, the population decreases linearly with the killing rate. It means that the population of committed cells follows a power law with exponent 1 when plotted against the ratio of division over killing rates.

The results of simulations with the ODE model with two populations (see section 2.5) are consistent with these theoretical results (Figure S17). For an ODE model with a Hill coefficient $n = 16$, the uncommitted

population is very robust to variations of the killing rate. The committed population is also robust (exponent of 0.07, close the theoretical value $1/n = 0.0625$) for high ratio, but follows exactly a linear dependence (exponent of 1.00) for high killing rate.

But more interestingly, the results of the stochastic simulations with the Langevin model are qualitatively similar (Figure S17). If the three systems show small differences for low ratio k_b/k_k, the fits of System 2 – which is the closest to the ODE model and have power laws with exponents 0.06 and 1.14 for respectively high and low ratio. These values are very close to both the theoretical analysis and the ODE simulations of the simplified model.

References

[1] Canton B, Labno A, Endy D (2008) Refinement and standardization of synthetic biological parts and devices. Nat Biotechnol 26: 787–793.

[2] Shetty R, Endy D, Knight Jr T (2008) Engineering BioBrick vectors from BioBrick parts. J Biol Eng 2: 5.

[3] Fujikura J, Yamato E, Yonemura S, Hosoda K, Masui S, et al. (2002) Differentiation of embryonic stem cells is induced by GATA factors. Genes Dev 16: 784-789.

[4] Schmitz ML, Baeuerle PA (1991) The p65 subunit is responsible for the strong transcription activating potential of nf-kappa b. EMBO J 10: 3805-3817.

[5] Arai R, Ueda H, Kitayama A, Kamiya N, Nagamune T (2001) Design of the linkers which effectively separate domains of a bifunctional fusion protein. Protein Eng 14: 529-532.

[6] Collins CH, Arnold FH, Leadbetter JR (2005) Directed evolution of vibrio fischeri luxr for increased sensitivity to a broad spectrum of acyl-homoserine lactones. Mol Microbiol 55: 712-723.

[7] Weiss R, Homsy GE, Knight Jr TF (1999). Toward in vivo digital circuits. DIMACS Workshop on Evolution as Computation.

[8] Gradner TS, Cantor CR, Collins JJ (2000) Construction of a genetic toggle switch in escherichia coli. Nature 403: 339-342.

[9] Coleman JE, Huentelman MJ, Kasparov S, Metcalfe BL, Paton JFR, et al. (2003) Efficient large-scale production and concentration of hiv-1-based lentiviral vectors for use in vivo. Physiol Genomics 12: 221-228.

[10] Miyoshi H, Blomer U, Takahashi M, Gage FH, Verma IM (1998) Development of a self-inactivating lentivirus vector. J Virol 72: 8150-8157.

[11] Smith H (1995) Monotone Dynamical Systems. Providence, RI: AMS.

[12] Angeli D, Sontag E (2004) Multistability in monotone input/output systems. Syst Control Lett 51: 185–202.

[13] Gillespie DT (2007) Stochastic simulation of chemical kinetics. Annu Rev Phys Chem 58: 35-55.

[14] Burrage K, Burrage PM (1996) High strong order explicit Runge-Kutta methods for stochastic ordinary differential equations. Appl Numer Math 22: 81 - 101.

[15] Gillespie DT (2000) The chemical langevin equation. J Chem Phys 113: 297-306.

[16] Hasty J, Isaacs F, Dolnik M, McMillen D, Collins J (2001) Designer gene networks: Towards fundamental cellular control. Chaos 11: 207.

[17] Hasty J, McMillen D, Collins J (2002) Engineered gene circuits. Nature 420: 224–230.

[18] McMillen D, Kopell N, Hasty J, Collins J (2002) Synchronizing genetic relaxation oscillators by intercell signaling. Proc Natl Acad Sci USA 99: 679.

[19] Stricker J, Cookson S, Bennett M, Mather W, Tsimring L, et al. (2008) A fast, robust and tunable synthetic gene oscillator. Nature 456: 516–519.

[20] Swinburne I, Miguez D, Landgraf D, Silver P (2008) Intron length increases oscillatory periods of gene expression in animal cells. Genes Dev 22: 2342–2346.

[21] Tigges M, Marquez-Lago T, Stelling J, Fussenegger M (2009) A tunable synthetic mammalian oscillator. Nature 457: 309–312.

[22] Hsu A, Vijayan V, Fomundam L, Gerchman Y, Basu S, et al. (2005) Dynamic Control in a Coordinated Multi-Cellular Maze Solving System (I). In: American Control Conference. volume 6, pp. 4399-4404.

[23] Basu S, Mehreja R, Thiberge S, Chen M, Weiss R (2004) Spatiotemporal control of gene expression with pulse-generating networks. Proc Natl Acad Sci USA 101: 6355-6360.

[24] Greber D, Fussenegger M (2007) Mammalian synthetic biology: Engineering of sophisticated gene networks. J Biotechnol 130: 329-345.

[25] Brint J, Ohman D (1995) Synthesis of multiple exoproducts in Pseudomonas aeruginosa is under the control of RhlR-RhlI, another set of regulators in strain PAO1 with homology to the autoinducer-responsive LuxR-LuxI family. J Bacteriol 177: 7155.

[26] Engebrecht J, Nealson K, Silverman M (1983) Bacterial bioluminescence: isolation and genetic analysis of functions from Vibrio fischeri. Cell 32: 773–81.

[27] You L, Cox III R, Weiss R (2004) Programmed population control by cell- cell communication and regulated killing. Nature 428: 868–871.

[28] Weber W, Schoenmakers R, Spielmann M, El-Baba M, Folcher M, et al. (2003) Streptomyces-derived quorum-sensing systems engineered for adjustable transgene expression in mammalian cells and mice. Nucleic Acids Res 31: e71.

[29] Chen M, Weiss R (2005) Artificial cell-cell communication in yeast Saccharomyces cerevisiae using signaling elements from Arabidopsis thaliana. Nat Biotechnol 23: 1551–1555.

[30] Brenner K, Karig D, Weiss R, Arnold F (2007) Engineered bidirectional communication mediates a consensus in a microbial biofilm consortium. Proc Natl Acad Sci USA 104: 17300.

[31] Li G, Hu J, Wang SW, Georgopoulos PG, Schoendorf J, et al. (2006) Random sampling-high dimensional model representation (rs-hdmr) and orthogonality of its different order component functions. J Phys Chem A 110: 2474-2485.

[32] Chatterjee S, Price B (1977) Regression analysis by example. Wiley New York.

[33] Chong I, Jun C (2005) Performance of some variable selection methods when multicollinearity is present. Chemometr Intell Lab 78: 103–112.

[34] Ciaccio M, Wagner J, Chuu C, Lauffenburger D, Jones R (2010) Systems analysis of EGF receptor signaling dynamics with microwestern arrays. Nat Methods 7: 148–155.

[35] Eaton D, Murphy K (2007) Exact Bayesian structure learning from uncertain interventions. In: AI & Statistics. Citeseer, volume 2, pp. 107–114.

[36] Koivisto M, Sood K (2004) Exact Bayesian structure discovery in Bayesian networks. J Mach Learn Res 5: 573.

[37] Gillespie D (1977) Exact stochastic simulation of coupled chemical reactions. J Chem Phys 81: 2340–2361.
